# Diene Substitution Patterns in the Conformation‐Activity Relationships of GEX1A

**DOI:** 10.1002/cmdc.70309

**Published:** 2026-05-22

**Authors:** Christian A. Umaña, Matthew C. Rhodes, Jeffrey L. Henry, Duoming Ma, Taylor R. Quinn, Richard E. Taylor

**Affiliations:** ^1^ Department of Chemistry and Biochemistry and the Warren Center for Drug Discovery University of Notre Dame Notre Dame Indiana USA

**Keywords:** cancer, conformation, GEX1A, polyketide, splicing

## Abstract

GEX1A (herboxidiene) is a polyketide natural product that possesses potent biological activities for a number of indications. GEX1A binds the splicing factor 3B subunit 1 (SF3B1), inhibiting the splicing of pre‐mRNA. Moreover, we have reported that GEX1A reverses cholesterol accumulation in Niemann–Pick Type C mutant fibroblasts. Several groups have investigated the structure–activity relationships of this polyketide, mostly focusing on the tetrahydropyran core and the distal, oxygenated section of the side chain. Herein, we present the design, synthesis, and conformational studies of a series of analogues to explore substitution on the diene section of GEX1A. We employed computational and high‐field NMR studies to describe the analogues’ conformational landscape and their relative biological activities to describe their conformational‐activity profile. We found that modifications on the diene section brought significant changes to both the molecule's conformation and biological activity.

## Graphical abstract

Synthetic analogues of GEX1A were designed and synthesised in an effort to explore the significance of diene substitution on the conformational preferences and expand our understanding of the three‐dimensional pharmacophore for this biologically active natural product. Chemical synthesis provided access to unique structural analogues of the natural product GEX1A (herboxidiene) with a focus on the methyl substitution pattern within the core diene unit. Computational modelling coupled with NMR data provided an understanding of the solution conformation for each GEX1A congener. The results of this study demonstrate that during the evolution of polyketide structures, olefin substitution contributes to both the molecule's overall conformational profile as well as discrete structural interactions with its protein‐binding partner.

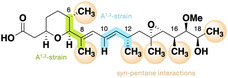

## 1 Introduction

**FIGURE 1:**
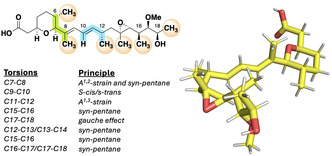
The conformation of GEX1A in the solid state. The observed conformation is an additive result of classic molecular conformational features.

The structural architecture of polyketide natural products encompasses an array of chemical diversity that contributes to their fascinating biological activities. While polyketides such as erythromycin, pikromycin, amphotericin, rifamycin, bafilomycin and many others have been extensively studied from a medicinal chemistry perspective, the systematic and exhaustive structural modification of their polyketide skeletons remains relatively underexplored. Studies that investigate the structure–activity relationships (SARs) of polyketide natural products rarely consider the relationship between the structure's preferred solution conformation, its protein‐bound conformation, and its biological activity. This fact is almost certainly due to a misconception about their presumed flexibility. In polyketide natural products, enzymatic machinery integrates unique structural features (e.g. stereogenic centres, geometry‐defined olefins, hydrogen bonding hydroxyl groups and ester linkages, as well as both small rings, macrocycles, and heterocycles) that serve to restrict torsional rotation, promoting preferred conformations. Thus, even minor substitution or stereochemical inversion may significantly affect and alter the binding affinity and biological potency. Therefore, conformational considerations must be incorporated into strategic design principles [1, 2, 3, 4, 5]. GEX1A (herboxidiene; **1**, Figure 1), a linear type‐I polyketide natural product, was originally isolated by Edmunds and co‐workers from the soil bacterium *Streptomyces chromofuscus* in 1992 [6]. Later, GEX1A **1** was found to exhibit potent cytotoxic effects across a range of cancer cell lines [7]. Mizukami and coworkers later uncovered GEX1A's mechanism of action to affect pre‐mRNA splicing through binding to SAP155, a key component of splicing factor 3B subunit 1 (SF3b1) [8]. Modulation of pre‐mRNA splicing of numerous important cell cycle regulators is believed to be responsible for the cytotoxic activity of **1** [7]. In addition to the intriguing antiproliferative activity, our laboratory disclosed GEX1A's potential as a lead for the treatment of Niemann–Pick Type C disease by the reversal of the cholesterol accumulation phenotype observed in NPC1 mutant fibroblasts [9]. More recently, we found that GEX1A sensitised FLT3‐ITD + leukemic cells to apoptosis by inducing aberrant splicing and repressing the expression of FLT3‐ITD. After finding that GE1XA had excellent in vitro pharmacological properties, we found that intraperitoneal injection of GEX1A significantly improved the survival of leukemic mice compared to the vehicle control group [10, 11].

For many years, we have been interested in the role of methyl‐substituted alkenes in polyketide natural products, including the epothilones, the pelorusides, dactylolide, and the zampanolides. We recently reported on the conformational role of the C17‐methyl group in dactylolide and zampanolide and its effect on the cytotoxicity of these macrocycles [12]. The presence of the methyl group increases the population of the bioactive conformation by adding critical A^1,3^‐strain and *syn*‐pentane torsional restrictions. Subsequent studies from our lab showed that increased flexibility, in general, does not necessarily lead to decreased cytotoxicity [13]. As another example, Seiple and coworkers noted the structural significance of the C12‐methyl group in the streptogramin antibiotics [14]. This type of conformation‐activity relationship study has yet to be reported for GEX1A but provides impetus to explore the bioactive conformation of these natural products and inform the design of unique analogues.

The solid‐state conformation (Figure 1) appears to be controlled by classic molecular conformational features including the minimisation of allylic strain about the C7‐C8 (A^1,2^ and A^1,3^), C11‐C12(A^1,3^), and C15‐C16(*pseudo‐*A^1,3^) bonds, *syn*‐pentane interactions between methyl groups at C6 & C11, C12 & C14, C14 & C16, and C16 & C18, and a stabilising gauche effect of the C17, C18‐diol monomethyl ether. The Floreancig group probed the role of the C12‐methyl group in the formation of a potentially biologically significant conformational ‘turn’ observed in the solid state [15]. The C12‐desmethyl derivative was more than an order of magnitude less active than GEX1A. As is clear from the solid‐state conformation, C12‐methyl initiates the turn through a minimisation of the A^1,3^‐strain across the C11‐C12 torsion and the first of a series of 1,3‐*syn‐*pentane interactions with the methyl group at C14.

Intrigued by Floreancig's findings and their relationship to our long‐standing interest in the conformation‐activity relationships of polyketide natural products [1, 2, 3, 4, 5], we sought to expand our understanding of the GEX1A conformation and pharmacophore with a focus on the diene region of **1**. The paradigm includes two important and complementary components: 1. molecular modelling coupled with high‐field NMR analysis used to gain an understanding of the conformation preferences for specific rotatable bonds found within a target polyketide [16], and 2. the design of analogues which alter the molecule's conformation profile via minimal substitution [12, 17, 18, 19]. Subsequent biological evaluation of the designed analogue then allows for an improved understanding of the structural and conformational constraints of binding and resulting biological activity.

## 2 Results and Discussion

**FIGURE 2:**
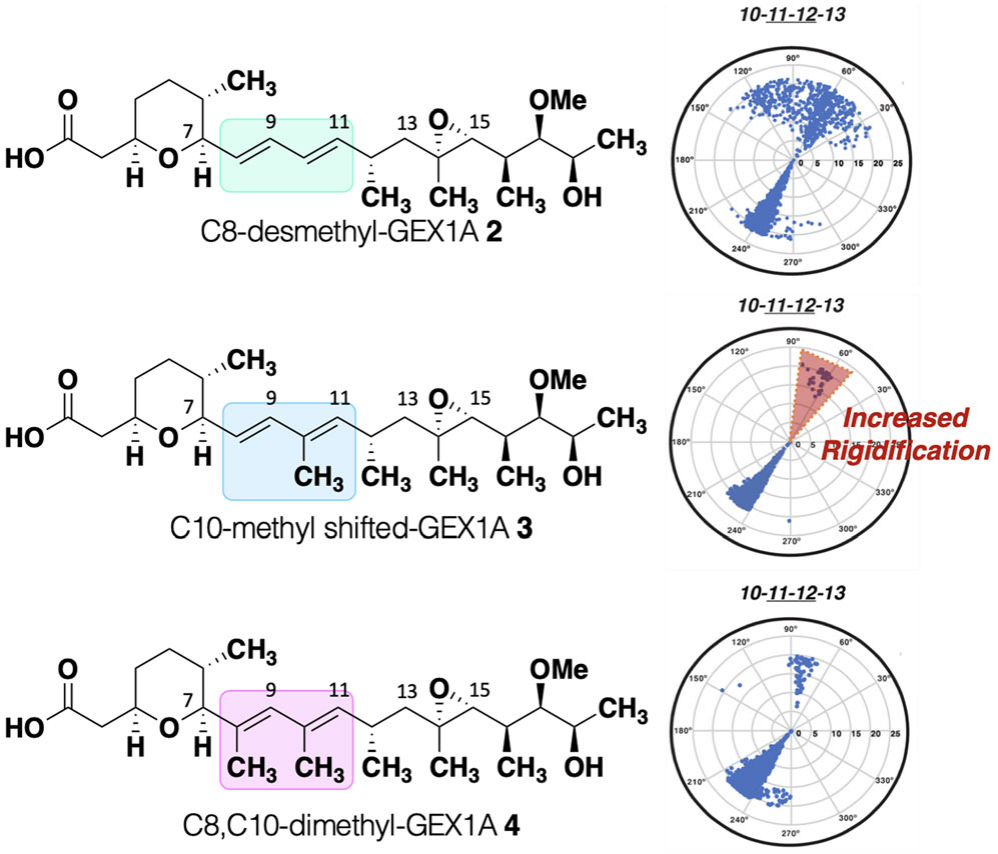
Proposed GEX1A analogues **2**, **3**, and **4**. Dihedral angle distribution plots for the analogues from computational modelling. A complete description of the polar plots can be found in the Supporting Information.

We hypothesised that by incrementing the rigidity of the ‘turn’ conformer, the activity of **1** may increase since a greater percentage of the conformational potential energy surface would have geometries related to the active conformation. We proposed the synthesis of three new analogues, C8‐desmethyl GEX1A **2**, C10‐methyl shifted GEX1A **3**, and C8, C10‐dimethyl GEX1A **4**. Desmethyl analogue **2** will provide insight into the effect of the C8‐methyl in the diene conformation, while analogues **3** and **4** will explore the effect of additional A^1,3^‐strain about the C11‐C12 torsion (Figure 2). We first conducted a comparative conformational search on each structure using a 50,000‐step Monte Carlo search employing the MM3* force field [20] in conjunction with the GB/SA solvation model for water. Structures within 4.7 kcal/mol of the global minimum were saved, and the duplicated conformers were eliminated to yield a potential energy surface of conformational families [21]. Analysis of the results using polar coordinate maps for each torsion around C12 revealed that a higher degree of torsional rigidity is observed for analogues **3** and **4**; meanwhile, analogue **2** displayed more torsional flexibility, as fully expected, particularly at the C7‐C8 torsion, resulting from the loss of A^1,3^‐strain‐based interactions.

**SCHEME 1:**
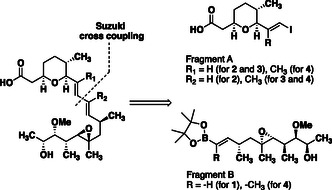
Retrosynthetic analysis for structural analogues of GEX1A. Details of the synthetic routes to each fragment are included in the Supporting Information.

Our synthetic route to the proposed conformational analogues **2, 3, 4** utilised a unique combination of transformations but built upon a foundation of several previous total syntheses of **1** [14, 22, 23, 24, 25, 26, 27, 28]. We selected a Suzuki cross‐coupling of fragments A and B (Scheme 1). The chosen strategy provided the advantage of a convergent synthesis and enabled late‐stage derivatisation of both fragments to provide access to the desired olefin substitution patterns.

**SCHEME 2:**
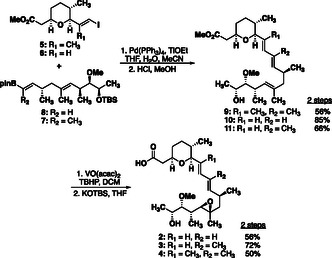
Assembly of fragments A and B for the synthesis of GEX1A analogues. Details of the synthetic routes for each analogue are included in the Supporting Information.

The synthetic routes to compounds **2**, **3** and **4** were accomplished under the identical conditions beginning with the Suzuki cross‐coupling (Scheme 2). Not surprisingly, the efficiency of the coupling was dependent upon the olefin substitution pattern. Traditionally, this reaction is performed in the presence of inorganic bases such as K_2_CO_3_ or Cs_2_CO_3_, but low yields were obtained (<10%). However, we found that the use of thallium ethoxide enabled fair to good yields (56–85%) at ambient temperatures in each case [29]. Purification by flash‐chromatography proved to be challenging for these compounds, and thus the crude materials were subjected to acidic methanol to deprotect the C18‐TBS ether in good yield. The presence of the deprotected alcohol provided better conditions for the purification of the compounds as the polarity increased column retention time, enabling separation from many less‐polar impurities. The epoxidation step was achieved with Kocienski's conditions [30] to afford the methyl esters with moderate yield (∼50–72%) as the reactions were not allowed to reach completion to avoid the overoxidation. Saponification with KOTBS in THF provided the corresponding carboxylic acids in quantitative yield.

**FIGURE 3:**
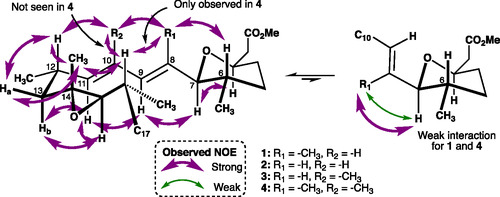
Selected NOEs for GEX1A and conformation analogues.

With each analogue in hand, we were in a position to study their conformational preferences through a combination of solution NMR studies built upon prior computational analysis. Two‐dimensional NMR experiments were performed on the methyl ester derivatives of compounds **2**, **3**, and **4** in MeOD‐d_4_. ROESY experiments were acquired in MeOD‐d_4_ at 800 MHz with mixing times of 100, 250, and 500 ms, enabling discrimination between strong and weak NOE correlations. Within the C10‐C13 section (Figure 3), strong NOE cross‐peaks showed high resemblance between all the analogues, indicating that the compounds’ solution conformation did not significantly deviate from the classical GEX1A ‘turn’ conformation (vide supra) observed in the solid‐state and in solution.

In contrast, conformational differences were observed with regard to the flexibility of the C7‐C8 bond. A strong NOE cross‐peak was observed between H7 and H8 (for compounds **2** and **3**), while GEX1A showed only a weak spatial interaction [31]. This observation can be explained by the modulation of the A^1,2^‐strain, in general, and the elimination of a rigidifying *syn*‐pentane between C6 and C8 methyl groups, specifically, allowing a higher degree of rotation of the C7‐C8 allylic torsion. For the C8‐C11 diene section, strong cross‐peaks between H8‐H10 (for **2**) and H8‐C10Me (for **3**) were seen along with a strong NOE correlation between H9 and H11, suggesting the expected *s‐trans* configuration about the diene. No cross‐peaks were seen for C8Me and C10Me in **4**, although the absence of an NOE correlation between methyl groups is not definitive. It has been reported that methyl groups tend to leak magnetisation by raising their spinning rate, disturbing the cross‐relaxation and lowering the quality of the methyl–methyl interactions [32]. Our computational calculations predicted that the diene methyl substituents in **4** would adopt a non‐planar conformation [33], evidenced by the interaction of C8Me and H16, not seen in **2** and **3**. Interestingly, no cross‐peak was observed between C10Me and H12 in **4**, in contrast to GEX1A, **2**, and **3**. The combination of these two observations is suggestive that the non‐planar diene of **4** exhibits specific axial chirality with R1 rotated towards H16 and R2 rotated away.

In addition to the NOE data, the conformational preferences can be inferred from vicinal coupling constants. For example, the torsional rigidity of the C11 and C12 bond could be inferred from their coupling constant value between H11 and H12 (Table 1). Theoretical calculations for an antiperiplanar alignment of these protons provided 11.2 Hz [34]. Deviations from this maximum value would require the inclusion of conformers with alternative relative orientations.

**TABLE 1:** Proton coupling constants observed in MeOH‐*d*_4_ for selected protons within GEX1A and methyl ester analogues.

**Vicinal Coupling** a	GEX1A	2	3	4
H7, H8	NA	7.4	7.9	NA
H11, H12	9.0	8.6	10.0	9.9
H12, H13a	4.3	4.4	4.0	4.4
H12, H13b	10.9	10.8	11.1	11.3

a Coupling constant (^3^ *J*) measured in Hz.

The H11–H12 coupling constant for the natural product, GEX1A, is 9.0 Hz, suggesting some flexibility about the torsion, with an anti‐periplanar orientation being predominant. The coupling constant increased in analogues **3** and **4**, denoting that these structures have more rigidity around the C11‐C12 torsion than GEX1A. In addition, the H7–H8 coupling constant observed for analogues **2** and **3** suggests that methyl substitution at C9 only slightly rigidifies the conformational flexibility of the C7‐C8 torsion observed in the unsubstituted analogue **2**. Overall, the introduction of a C10 methyl group, in analogues **3** and **4**, does increase the rigidity of the C11‐C12 torsion due to increased A^1,3^‐strain and the minimisation of *syn*‐pentane interactions between the methyl groups at C10 and C12.

We next sought to correlate changes in conformational preferences of GEX1A and conformational analogues with their respective biological activity. For simplicity, we focused on a growth inhibition (GI_50_) assay were tested in cervical (HeLa), and acute myeloid leukaemia (Molm‐13) cell lines (Table 2). Unfortunately, all three analogues demonstrated a loss in potency relative to GEX1A, although compounds **3** and **4** were more significant than **2**. We find the loss of activity quite intriguing as this occurs despite relatively minor structural changes and the fact that each of these analogues continues to populate the putative bound conformation.

**TABLE 2:** Growth inhibition (GI_50_) of GEX1A and conformational analogues.

**GI**_**50**_	HeLa, μM	Molm‐13, μM
GEX1A **1** a	0.013	0.004
**2** b	6.94	4.17
**3** b	‐‐	461
**4** b	1790	107

a Sourced through isolation via fermentation of *S.* *chromofuscus*.

b Accessed through total synthesis.

The elimination of the C8 methyl group in analogues **2** and **3** certainly creates a flatter potential energy surface and a resulting increase in conformational flexibility about the C6‐C7 torsion. Interestingly, this seems to correlate with the fact that Webb has observed the structural importance of the C6‐methyl group, as a C6‐desmethyl GEX1A derivative exhibited a tenfold reduction in activity [35]. Both of these changes affect the A^1,2^‐strain controlling rotation about the C7‐C8 torsion. However, as we have recently shown in our study of *linear*‐zampanolide, increased flexibility does not fully correlate to decreased biological activity [13]. Though the C6‐ and C8‐methyl groups may play a more significant role in binding than simply conformationally‐interacting steric elements.

**FIGURE 4:**
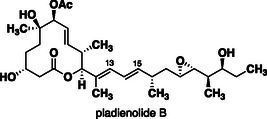
Structure of pladienolides B **12**.

While the co‐crystal structure of GEX1A and its binding ribonuclear protein, SF3b, has not been reported (For a useful discussion of computer modelling of GEX1A to the binding site of the X‐ray crystal structure of pladienolide to SF3B1 see) [36], the cryo‐EM structures for pladienolide B **12** with the PHF5A‐SF3B1 substructure have been studied [37, 38]. Molecular dynamics simulations calculated with the cryo‐EM data from E7107 predicted that GEX1A could fit within the same binding pocket, with a planar diene moiety as an essential motif due to its tight binding in a narrow slit at the PHF5A–SF3B1 interface, with a critical interaction with tyrosine‐36 via π‐stacking [39]. While even small structural differences can lead to major changes in binding orientations of small molecules within protein binding sites, a similar binding mode is worth considering, especially considering the bound conformation of pladienolide B sidechain mimics the solution conformation of GEX1A. Interestingly, the C12‐methyl group of pladienolide B maintains significant hydrophobic contacts within the binding site of SF3B1 (Figure 4). This corresponds to the C8‐position in GEX1A. Thus, the elimination of these interactions could account for significant loss of activity observed for the C8‐desmethyl analogues **2** and **3** and thus may be related to favourable hydrophobic interactions. Analogues **3** and **4,** which contain an additional methyl group at C10, may have introduced new steric clashes within the protein interface despite increasing the population of the putative bound conformation. However, we cannot rule out the fact that the newly introduced C10‐methyl group in analogue **4** may influence the planarity of the diene. A scan of the Cambridge crystallographic database includes several structures containing planar 1,3‐dimethyl‐1,3‐butadiene units.

## 3 Conclusion

Chemical synthesis provided access to unique structural analogues of the natural product GEX1A (herboxidiene) through manipulation of the methyl substitution pattern of the core diene unit. Introduction or removal of methyl groups on the diene section modulated both the solution conformation as well as the biological activity of the compounds, and further the interesting discussion of the ‘magic methyl’ effect [40]. In the case of conformational analogues **2** and **3**, removal of the C8‐methyl group not only increased conformational flexibility about the C7‐C8 allylic torsion, but, in addition, likely eliminated key binding interactions with the protein target SF3B1. As designed, the addition of a new methyl substituent at C10 increased the population of the likely bound conformation in solution but may have introduced unfavourable steric interactions with its protein binding partners. The work complements previously published SARs on GEX1A, which have not explored modifications to the diene at the current level of detail. Moreover, this effort furthers our laboratory's efforts to study conformation‐activity relationships in complex natural product medicinal chemistry. The results of this study demonstrate that during the evolution of polyketide structures, olefin substitution contributes to both the molecule's overall conformational profile as well as discrete structural interactions with its protein‐binding partner.

## Funding

This study was supported by National Institutes of Health (GM147637, T32GM075762).

## Conflicts of Interest

The authors declare no conflicts of interest.

## Supporting information

Supplementary Material

## Data Availability

The data that support the findings of this study are available on request from the corresponding author. The data are not publicly available due to privacy or ethical restrictions.
