# Supplementary material for: Diene Substitution Patterns in the Conformation‐Activity Relationships of GEX1A
Source: ChemMedChem. 2026 May 22;21(10):e70309. doi: 10.1002/cmdc.70309 (PMC13206506; doi:10.1002/cmdc.70309)

Supporting Information
©Wiley-VCH 2021
69451 Weinheim, Germany

Diene Substitution Patterns in the Conformation-Activity Relationships of GEX1A

Christian A. Umaña[a], Matthew C. Rhodes[a], Jeffrey L. Henry[a], Duoming Ma[a], Taylor R. Quinn [a], and Richard E. Taylor*[a]

[a] C. A. Umaña, Dr. J. L. Henry, Dr. M. C. Rhodes, D. Ma, T. R. Quinn, Prof. Dr. R. E. Taylor
Department of Chemistry and Biochemistry and the Warren Center for Drug Discovery
University of Notre Dame
Notre Dame, IN 46556-5670, USA
E-mail: rtaylor@nd.edu

Abstract: GEX1A (herboxidiene) is a polyketide natural product that possess potent biological activities for a number of indications. GEX1A binds the splicing factor 3B subunit 1 (SF3B1), inhibiting the splicing of pre-mRNA. Moreover, we have reported that GEX1A reverses cholesterol accumulation in Niemann-Pick Type C mutant fibroblasts. Several groups have investigated the structure-activity relationships (SAR) of this polyketide, mostly focusing in the tetrahydropyran core and the distant section of the side chain. Herein, we present that design, synthesis, conformational studies of a series of analogs to explore substitution on the diene section of GEX1A. We employed computational and high-field NMR studies to describe the analogs' conformational landscape, and their relative biological activities to describe their conformational-activity profile. We found that modifications on the diene section brought significant changes to both the molecule's conformation and biological activity.

Table of Contents

Dihedral Angle Distribution Polar Plots *2*

Experimental Procedures and Compound Characterization *4*

Growth Inhibition Assay *14*

References *15*

^{1}H and ^{13}C spectra *16*

Dihedral Angle Distribution Polar Plots

Figure 1 shows the basic idea behind the dihedral polar plots, which offer an elegant graphical method to analyze the data produced by the computational model search.[1] In a set of four atoms in which the 2-3 bond can rotate, a **Φ** angle (dihedral) between the 1-2 and 3-4 planes can be used to describe the conformational preferences of the 2-3 bond. The conformational search generates single conformers that are represented by a blue dot and which are plotted relative to the lowest conformation energy at the origin; the potential energy surface created by the model is limited to 20 kJ/mol (Represented by concentric circles in 5kJ/mol steps). For each conformer, the dihedral angles for each rotatable bond are extracted and represented in the polar plot for each specific angle.

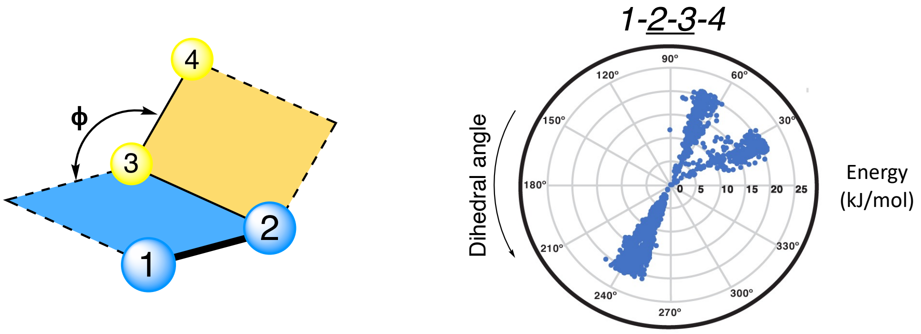

Figure 1. Polar Coordinate Map Representation of Computation Modelling Data.

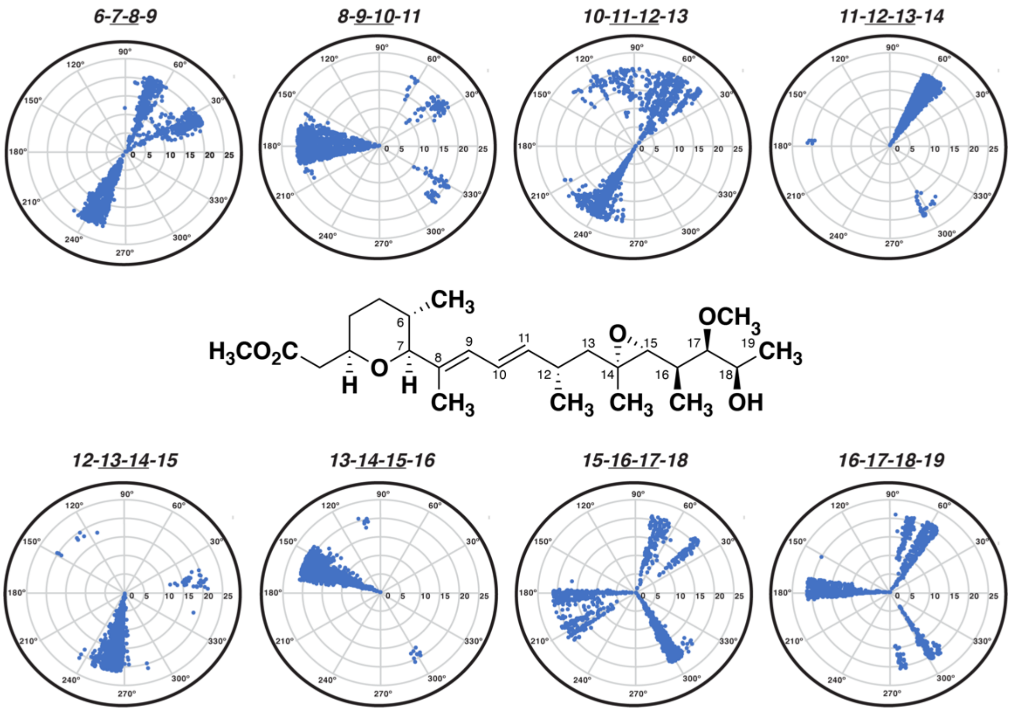

Figure 2. Dihedral angle distribution for GEX1A.

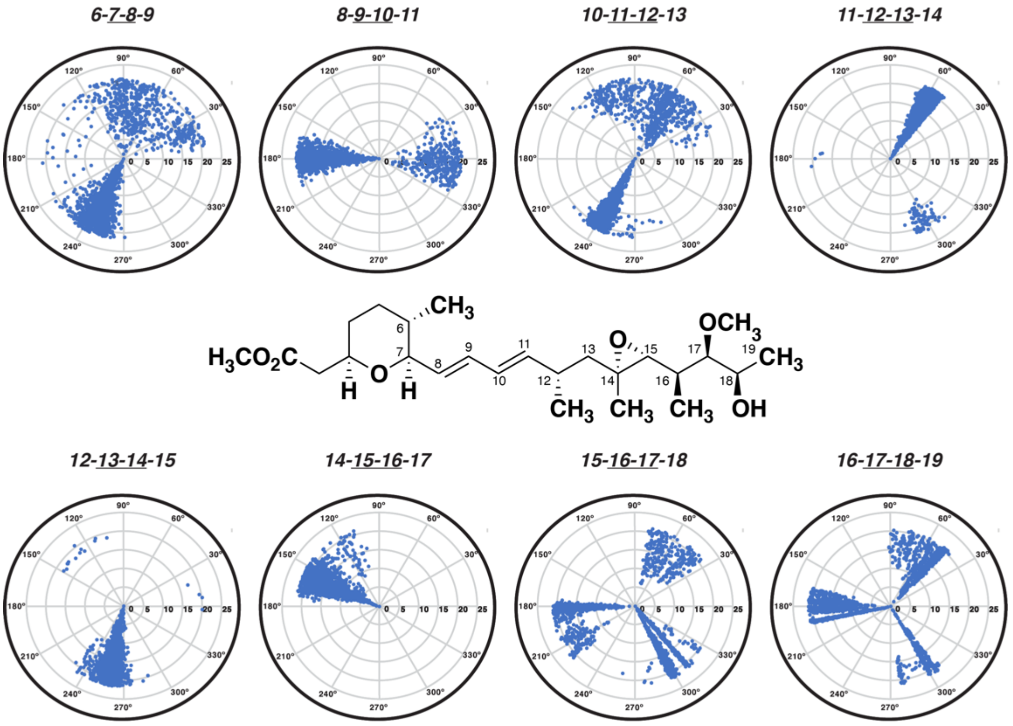

Figure 3. Dihedral angle distribution for C8-desmethyl GEX1A.

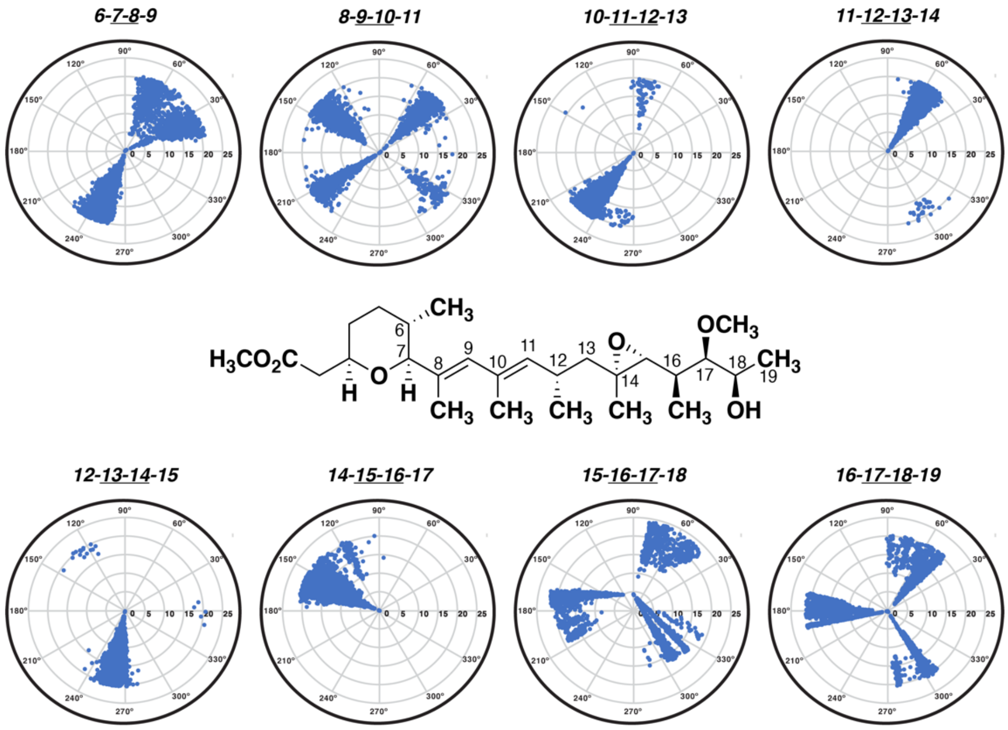

Figure 4. Dihedral angle distribution for C8,C10-dimethyl GEX1A.

Experimental Procedures and Compound Characterization

Unless otherwise noted, all materials were used as received from a commercial supplier without further purification. All anhydrous reactions were performed using oven-dried or flame-dried glassware, which was then cooled under vacuum and purged with argon gas. Tetrahydrofuran (THF), dichloromethane (DCM), toluene (PhMe), and diethyl ether (Et_2O) were filtered through activated alumina under argon. Triethylamine (TEA) and N,N-diisopropylethylamine (*i*-Pr_2NEt) were distilled over CaH_2 and stored over KOH pellets. 4 Å molecular sieves were oven-dried overnight and then cooled under high vacuum prior to use. All reactions were monitored by Silicycle analytical thin layer chromatography (TLC) plates (AL SIL G/UV, glass back) and analyzed with 254 nm UV light and/or anisaldehyde – sulfuric acid or potassium permanganate treatment. Silica gel for column chromatography was purchased from Silicycle (Silica Gel 60, 230-400 mesh). Unless otherwise noted, all ^{1}H and ^{13}C NMR spectra were recorded in $CDCl_3$ or MeOD d4 using either Bruker AVANCE III HD 500 operating at 500.13 MHz for ^{1}H and 125.77 MHz for ^{13}C, or Bruker AVANCE II 800 operating at 800.18 MHz for ^{1}H and 125.77 MHz for ^{13}C. Chemical shifts (δ) were reported in ppm relative to residual $CHCl_3$ as an internal reference (^{1}H: 7.26 ppm, ^{13}C: 77.16 ppm). Coupling constants (*J*) were reported in Hertz (Hz). Peak multiplicity is indicated as follows: s (singlet), d (doublet), t (triplet), q (quartet), p (pentet), b (broad), and m (multiplet). Mass spectra (HRMS-ESI) were obtained at the Department of Chemistry and Biochemistry, University of Notre Dame using either a JEOL AX505HA or JEOL JMS-GC mass spectrometer.

Fragment A

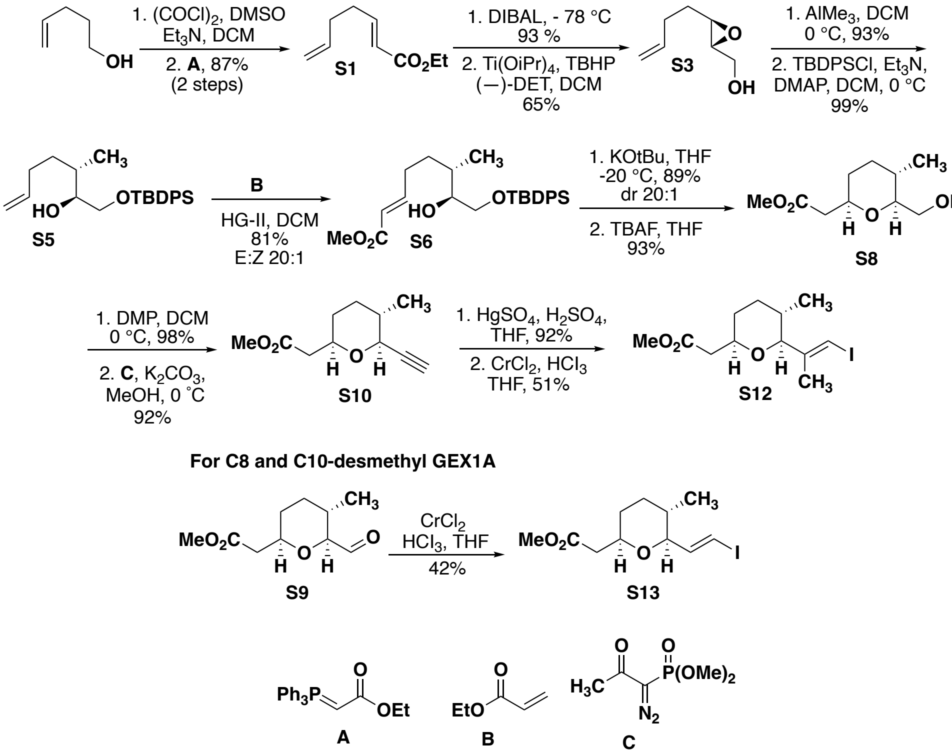

Scheme 1. Synthetic sequence for GEX1A (Fragment A).

ethyl (E)-hepta-2,6-dienoate (S1): Prepared according to reported procedures. [1,2] Dimethyl sulfoxide (21.0 mL, 293 mmol, 2.8 eq) was added in one portion to a solution of oxalyl chloride (13.7 mL, 157 mmol, 1.5 eq) in DCM (350 mL) under an argon atmosphere at -78 °C and stirred for 10 min. Then pent-4-en-1-ol (9.0 g, 105 mmol, 1.0 eq) in DCM (50 mL) was added dropwise and stirred for 15min. Triethylamine (81.5 mL, 585 mmol, 5.6 eq) was added dropwise to the solution, stirred for 30 min at –78°C, and then warmed to room temperature. Then (ethoxycarbonylmethylidene)triphenylphosphorane (55.7 g, 160 mmol, 1.5 eq) in DCM (110 mL) was added over 30 min. After stirring for 2h at room temperature, the reaction mixture was quenched with water (200 mL), the phases were separated, and the aqueous layer was extracted with DCM (3×50 mL). The combined organic layers were washed with brine (75 mL), dried over Na_2SO_4, filtered, and concentrated under reduced pressure to give a light-yellow oil. The remaining oil was dissolved in pentanes and placed at -20 ˚C for 2 h to crash out any remaining triphenylphosphine oxide. The pentane solution of this material was filtered through a short pad of silica gel and washed with ethyl acetate/hexane (1:9 v/v) solution to afford, after concentration of the filtrate, the ester **S1** (14.09 g, 87%) as a light-yellow oil.
^{1}H NMR (500 MHz, $CDCl_3$): δ 6.95 (dt, *J* = 15.6, 6.8 Hz, 1H), 5.83 (dt, *J* = 15.6, 1.6 Hz, 1H), 5.83 – 5.75 (m, 1H), 5.05 (dq, *J* = 17.1, 1.6 Hz, 1H), 5.00 (ddt, *J* = 10.2, 1.8, 1.2 Hz, 1H), 4.18 (q, *J* = 7.1 Hz, 2H), 2.34 – 2.26 (m, 2H), 2.24-2.18 (m, 2H), 1.28 (t, *J* = 7.1 Hz, 3H); **^{13}C NMR** (125 MHz, $CDCl_3$): δ 166.8, 148.4, 137.2, 121.8, 115.7, 60.3, 32.2, 31.6, 14.4; **HRMS-ESI** (m/z): calcd. for $C_9H_{15}O_2$ [M + H]+: 155.1067, found 155.1058; **TLC**: R_f = 0.70 with Hex:EtOAc (4:1). p-anisaldehyde (dark spot). Isolated as a clear oil.

ethyl (E)-hepta-2,6-dien-1-ol (S2): Prepared according to reported procedures. [1,2] DIBAL (172 mL, 172 mmol, 3.0 eq, 1.0 M in hexanes) was added dropwise to a solution of (E)-ethyl 2,6-heptadienoate **S1** (8.81 g, 57.1 mmol, 1.0 eq) in Et_2O (290 mL, 0.2 M) at -78 °C. The mixture was warmed to room temperature overnight. Next, the reaction was cooled to 0 °C, and a saturated solution of Rochelle's salt (200 mL) was added dropwise. The resulting mixture was warmed to room temperature and stirred until two distinct layers were observed. The phases were separated, and the aqueous layer was extracted with DCM (3×50 mL). The combined organic layers were washed with brine (100 mL), dried over Na_2SO_4, filtered, and concentrated under reduced pressure to give a light-yellow oil. Purification via flash chromatography (10% EtOAc/Hexanes to 30% EtOAc/Hexanes) afforded allylic alcohol **S2** (4.58 g, 40.8 mmol) in 93% yield as a colorless oil.
^{1}H NMR (500 MHz, $CDCl_3$): δ 5.87 – 5.75 (m, 1H), 5.74 – 5.61 (m, 2H), 5.02 (dt, *J* = 16.9, 1.7 Hz, 1H), 4.97 (dt, *J* = 10.0, 1.8 Hz, 1H), 4.08 (m, 2H), 2.14 (m, 4H); **^{13}C NMR** (125 MHz, $CDCl_3$): δ 138.2, 132.5, 129.5, 115.0, 63.9, 63.8, 33.4, 31.7; **HRMS-ESI** (m/z): calcd. for $C_7H_{12}NaO$ [M + Na]+: 135.0780, found 155.0776; **TLC**: R_f = 0.48 with Hex:EtOAc (1:1). p-anisaldehyde (blue spot). Isolated as a clear oil.

((2R,3R)-3-(but-3-en-1-yl)oxiran-2-yl)methanol (S3): Prepared according to reported procedures. [1,2] Diethyl D-(–)-tartrate (0.41 mL, 2.4 mmol, 0.15 eq) was added to a suspension of freshly activated powdered 4 Å molecular sieves (2.2 g) in dry DCM (150 mL) and cooled to –40 °C (dry ice in MeOH/H_2O (2:3)). Then $Ti(OiPr)_4$ (0.55 mL, 1.8 mmol, 0.11 eq) was added, followed by slow addition of t-butyl hydroperoxide (5.9 mL, 32.2 mmol, 2.0 eq, 5.5 M in nonane). The resulting mixture was stirred at –40 °C for 30 min then a solution of ethyl (E)-hepta-2,6-dien-1-ol **S2** (1.81 g, 51.7 mmol, 1.0 eq) in DCM (10 mL) was slowly added over 10 min. The reaction was stirred for 3h at –20 °C, then warmed to 0 °C and treated with water (80 ml). The mixture was warmed to room temperature and treated with aqueous 30% NaOH (6.0 mL, saturated with NaCl) for 1 h. The mixture was filtered through a small pad of Celite, the phases were separated, and the aqueous layer was extracted with DCM (3×30 mL). The combined organic layers were washed with brine (50 mL), dried over Na_2SO_4, filtered, and concentrated under reduced pressure to give a light-yellow oil. Purification via flash chromatography (10% EtOAc/Hexanes to 30% EtOAc/Hexanes) afforded the epoxy-alcohol **S3** (1.35 g, 10.5 mmol) in 65% yield as a colorless oil.
[α]$_D^{20}$ = =32.7 (c 1.78, $CHCl_3$); **^{1}H NMR** (500 MHz, $CDCl_3$): δ 5.83 (ddt, *J* = 16.9, 10.2, 6.6 Hz, 1H), 5.06 (dq, *J* = 17.1, 1.7 Hz, 1H), 5.03 – 4.97 (m, 1H), 3.91 (dt, *J* = 12.5, 3.3 Hz, 1H), 3.62 (dq, *J* = 12.4, 5.0, 4.3 Hz, 1H), 2.98 (td, *J* = 5.7, 2.3 Hz, 1H), 2.94 (dt, *J* = 4.3, 2.5 Hz, 1H), 2.30 – 2.13 (m, 2H), 1.76 (d, *J* = 7.3 Hz, 1H), 1.68 (td, *J* = 7.5, 5.7 Hz, 2H); **^{13}C NMR** (125 MHz, $CDCl_3$): δ 137.6, 115.5, 61.8, 58.6, 55.5, 31.0, 30.3; **HRMS-ESI** (m/z): calcd. for $C_7H_{12}NaO_2$ [M + Na]+: 151.0730, found 151.0748; **TLC**: R_f = 0.32 with Hex:EtOAc (1:1). p-anisaldehyde (pink spot). Isolated as a clear oil.

(2S,3S)-3-methylhept-6-ene-1,2-diol (S4): Prepared according to reported procedures. [1,2] Trimethylaluminium (9.5 mL, 19.1 mmol, 3.0 eq, 2.0 M in hexanes) was added dropwise to a stirred solution of epoxide **S3** (0.816 g, 6.36 mmol, 1.0 eq) in DCM (9 mL) at 0 °C under an argon atmosphere. After the addition was complete, the reaction mixture was stirred at 23 °C overnight, chilled to 0 °C, and quenched carefully with saturated NH_4Cl (1 mL). Once the bubbling ceased, a saturated solution of Rochelle's salt (50 mL) was added, warmed to room temperature, and stirred until two distinct layers were observed. The mixture was filtered through a small pad of Celite, the phases were separated, and the aqueous layer was extracted with DCM (3×20 mL). The combined organic layers were washed with brine (50 mL), dried over Na_2SO_4, filtered, and concentrated under reduced pressure to give a light-yellow oil. Purification via flash chromatography (10% EtOAc/Hexanes to 50% EtOAc/Hexanes) afforded the diol **S4** (0.854 g, 5.92 mmol) in 93% yield as a colorless oil.
[α]$_D^{20}$ = = -5.3 (c 0.75, $CHCl_3$); **^{1}H NMR** (500 MHz, $CDCl_3$): δ 5.87 – 5.74 (m, 1H), 5.02 (dq, *J* = 16.9, 1.8 Hz, 1H), 4.95 (dddt, *J* = 10.2, 3.4, 2.2, 1.2 Hz, 1H), 3.76 – 3.66 (m, 1H), 3.57 – 3.46 (m, 2H), 2.73 (s, 0H), 2.26 – 2.06 (m, 2H), 2.06 – 1.91 (m, 1H), 1.79 – 1.53 (m, 2H), 1.32 – 1.15 (m, 1H), 0.89 (ddd, *J* = 7.0, 3.2, 1.8 Hz, 3H); **^{13}C NMR** (125 MHz, $CDCl_3$): δ 138.9, 114.7, 76.3, 64.8, 35.7, 31.7, 31.2, 15.2; **HRMS-ESI** (m/z): calcd. for $C_8H_{16}NaO_2$ [M + Na]+: 167.1042, found 167.1036; **TLC**: R_f = 0.14 with Hex:EtOAc (1:1). p-anisaldehyde (dark spot). Isolated as a clear oil.

(2S,3S)-1-((tert-butyldiphenylsilyl)oxy)-3-methylhept-6-en-2-ol (S5): Prepared according to reported procedures. [1,2] Triethylamine (1.94 ml, 13.9 mmol, 2.2 eq) was added to a stirred solution of the diol **S4** (0.908 g, 6.30 mmol, 1.0 eq), TBDPSCl (2.08 g, 7.56 mmol, 1.2 eq) and DMAP (38 mg, 0.31 mmol, 0.05 eq) in DCM (32 mL) at 0 °C under an argon atmosphere. The reaction was warmed to room temperature and stirred overnight. The reaction was quenched with water (20 mL), the phases were separated, and the aqueous layer was extracted with DCM (3×20 mL). The combined organic layers were washed with brine (50 mL), dried over Na_2SO_4, filtered, and concentrated under reduced pressure to give a light-yellow oil. Purification via flash chromatography (Hexanes to 20% EtOAc/Hexanes) afforded the product **S5** (2.42 g, 6.34 mmol) in 99% yield as a colorless oil.
[α]$_D^{20}$ = +5.5 (c 1.18, $CHCl_3$); **^{1}H NMR** (500 MHz, $CDCl_3$): δ 7.77 – 7.62 (m, 4H), 7.50 – 7.33 (m, 6H), 5.79 (ddt, *J* = 16.9, 10.2, 6.6 Hz, 1H), 4.99 (dq, *J* = 17.1, 1.7 Hz, 1H), 4.93 (ddt, *J* = 10.2, 2.3, 1.2 Hz, 1H), 3.73 (dd, *J* = 10.0, 3.1 Hz, 1H), 3.57 (dd, *J* = 9.9, 7.9 Hz, 1H), 3.51 (ddd, *J* = 8.0, 6.5, 3.1 Hz, 1H), 2.58 (s, 1H), 2.13 (ddddd, *J* = 13.1, 8.0, 6.7, 4.8, 3.3 Hz, 1H), 2.04 – 1.89 (m, 1H), 1.63 (ddtd, *J* = 15.4, 13.0, 6.6, 3.8 Hz, 2H), 1.25 – 1.17 (m, 1H), 1.07 (s, 9H), 0.79 (d, *J* = 6.8 Hz, 3H); **^{13}C NMR** (125 MHz, $CDCl_3$): δ 139.1, 135.7, 135.7, 134.9, 133.3, 133.3, 130.0, 130.0, 129.8, 127.9, 127.9, 114.4, 75.7, 66.1, 35.2, 31.7, 31.3, 27.0, 26.7, 19.4, 15.2; **HRMS-ESI** (m/z): calcd. for $C_{24}H_{34}NaO_2Si$ [M + Na]+: 405.2220, found 405.2219; **TLC**: R_f = 0.77 with Hex:EtOAc (1:1). p-anisaldehyde (green spot). Isolated as a clear oil.

methyl (6S,7S,E)-8-((tert-butyldiphenylsilyl)oxy)-7-hydroxy-6-methyloct-2-enoate (S6): Prepared according to reported procedures. [3,4] To a solution of olefin **S5** (0.218 g, 0.57 mmol, 1.0 eq) and freshly distilled methyl acrylate (0.62 mL, 6.87 mmol, 12 eq) in degassed DCM (0.35 mL, 1.6 M), Hoveyda-Grubbs' catalyst 2nd generation (19 mg, 0.03 mmol, 0.05 eq) was added in one portion under an argon atmosphere. The solution was refluxed for 12 h, cooled to room temperature, and then imidazole (50 mg) was added and stirred for 30 min to crash out the excess catalyst. Water (20 mL) was added, the phases were separated, and the aqueous layer was extracted with DCM (3×10 mL). The combined organic layers were washed with brine (30 mL), dried over Na_2SO_4, filtered, and concentrated under reduced pressure to give a dark oil. Purification via flash chromatography (5% EtOAc/Hexanes to 80% EtOAc/Hexanes) afforded the product **S6** (202 mg, 0.46 mmol, *E:Z* ratio > 20:1) in 81% yield as a colorless oil.
[α]$_D^{20}$ = -2.4 (c 1.18, $CHCl_3$); **^{1}H NMR** (500 MHz, $CDCl_3$): δ 7.71 – 7.62 (m, 4H), 7.48 – 7.36 (m, 6H), 6.95 (dt, *J* = 15.6, 6.9 Hz, 1H), 5.81 (dt, *J* = 15.7, 1.6 Hz, 1H), 3.72 (s, 3H), 3.71 – 3.69 (m, 1H), 3.55 (dd, *J* = 10.1, 7.8 Hz, 1H), 3.47 (ddd, *J* = 8.0, 7.0, 3.2 Hz, 1H), 2.74 – 2.44 (m, 1H), 2.27 (ddddd, *J* = 15.2, 10.1, 6.9, 5.2, 1.7 Hz, 1H), 2.17 – 2.07 (m, 1H), 1.71 (dddd, *J* = 13.7, 10.2, 6.5, 3.9 Hz, 1H), 1.59 (dtd, *J* = 9.0, 6.8, 3.9 Hz, 1H), 1.34 – 1.20 (m, 1H), 1.06 (s, 9H), 0.77 (d, *J* = 6.8 Hz, 3H); **^{13}C NMR** (125 MHz, $CDCl_3$): δ 167.3, 149.7, 135.7, 135.7, 133.2, 133.2, 130.0, 130.0, 128.0, 121.0, 75.6, 66.1, 51.5, 35.2, 30.8, 29.7, 27.0, 19.4, 15.2; **HRMS-ESI** (m/z): calcd. for $C_{26}H_{36}NaO_4Si$ [M + Na]+: 463.2275, found 463.2269; **TLC**: R_f = 0.45 with Hex:EtOAc (4:1). p-anisaldehyde (blue spot). Isolated as a clear oil.

methyl 2-((2R,5S,6S)-6-(((tert-butyldiphenylsilyl)oxy)methyl)-5-methyltetrahydro-2H-pyran-2-yl)acetate (S7): The methyl ester **S6** (1.972 g, 4.48 mmol, 1.0 eq) was dissolved in THF (45 mL) and potassium tert-butoxide (0.28 mL, 0.45 mmol, 0.1 eq, 1.6 M in THF) was added dropwise over 15 minutes at 0 °C. The reaction was stirred for 30 min at 0 °C and quenched slowly with saturated NH_4Cl. The solution was warmed to room temperature, the phases were separated, and the aqueous layer was extracted with Et_2O (3×20 mL). The combined organic layers were washed with brine (30 mL), dried over Na_2SO_4, filtered, and concentrated under reduced pressure to give a dark oil. Purification via flash chromatography (10% Et_2O/Hexanes) afforded the product **S7** (1.76 g, 3.99 mmol, dr > 20:1) in 89% yield as a colorless oil.
$[\alpha]_D^{20}$ = +6.1 (c 2.67, $CHCl_3$); **^{1}H NMR** (500 MHz, $CDCl_3$): δ 7.76 – 7.68 (m, 4H), 7.43 – 7.36 (m, 6H), 3.79 (dd, *J* = 11.2, 2.4 Hz, 1H), 3.79 – 3.74 (m, 1H), 3.73 (dd, *J* = 11.3, 5.0 Hz, 1H), 3.07 (ddd, *J* = 9.9, 4.9, 2.3 Hz, 1H), 2.59 (dd, *J* = 15.1, 7.6 Hz, 1H), 2.42 (dd, *J* = 15.1, 5.5 Hz, 1H), 1.79 (dq, *J* = 13.0, 3.5 Hz, 1H), 1.69 – 1.57 (m, 2H), 1.34 (tdd, *J* = 12.8, 11.0, 3.7 Hz, 1H), 1.27 – 1.19 (m, 1H), 1.05 (s, 9H), 0.81 (d, *J* = 6.6 Hz, 3H); **^{13}C NMR** (125 MHz, $CDCl_3$): δ 172.1, 135.9, 135.8, 134.9, 134.2, 134.1, 129.8, 129.6, 127.9, 127.7, 127.6, 84.4, 74.2, 65.4, 51.7, 41.6, 32.7, 31.8, 31.1, 26.9, 19.5, 17.7; **HRMS-ESI** (m/z): calcd. for $C_{26}H_{37}O_4Si$ [M + H]+: 441.2455, found 441.2442; **TLC**: R_f = 0.43 with Hex:EtOAc (9:1). p-anisaldehyde (blue spot). Isolated as a clear oil.

methyl 2-((2R,5S,6S)-6-(hydroxymethyl)-5-methyltetrahydro-2H-pyran-2-yl)acetate (S8): Prepared according to reported procedures. [5] Silyl ether **S7** (4.42 g, 10.0 mmol, 1.0 eq) was dissolved in THF (50 mL) and TBAF (6.33 g, 20 mmol, 2.0 eq) was added in one portion and the reaction stirred at rt for 4 h. Water was added and the aqueous phase was extracted with Et_2O. The organic fractions were dried with Na_2SO_4, filtered, and concentrated. The crude product was purified by flash chromatography on silica gel eluting side products with 5% EtOAc:Hexanes then flushing with 30% EtOAc:Hexanes to afford the product **S8** (1.88 g, 9.3 mmol) in 93% as a clear oil.
$[\alpha]_D^{20}$ = +7.9 (c 1.0, $CHCl_3$); **^{1}H NMR** (500 MHz, $CDCl_3$): δ 3.80 (dddd, *J* = 11.1, 7.7, 5.3, 2.1 Hz, 1H), 3.74 (dd, *J* = 11.4, 2.8 Hz, 1H), 3.69 (s, 3H), 3.13 (ddd, *J* = 9.9, 7.1, 2.8 Hz, 1H), 2.57 (dd, *J* = 15.2, 7.8 Hz, 1H), 2.44 (dd, *J* = 15.2, 5.3 Hz, 1H), 2.18 (s, 1H), 1.85 – 1.78 (m, 1H), 1.73 – 1.66 (m, 1H), 1.46 (dddd, *J* = 18.0, 13.2, 6.6, 3.9 Hz, 1H), 1.35 (tdd, *J* = 12.2, 10.8, 3.4 Hz, 1H), 1.26 (tdd, *J* = 13.1, 11.4, 3.4 Hz, 1H), 0.84 (d, *J* = 6.6 Hz, 3H); **^{13}C NMR** (125 MHz, $CDCl_3$): δ 171.8, 83.6, 74.1, 63.9, 51.8, 41.32, 32.3, 31.7, 31.3, 17.2; **HRMS-ESI** (m/z): calcd. for $C_{10}H_{19}O_4$ [M + H]+: 203.1278, found 203.1276; **TLC**: R_f = 0.25 with Hex:EtOAc (7:3). p-anisaldehyde (blue spot). Isolated as a clear oil.

methyl 2-((2R,5S,6S)-6-formyl-5-methyltetrahydro-2H-pyran-2-yl)acetate (S9): o-Iodoxy-benzoic acid (IBX) (2.21 g, 7.91 mmol, 2.0 eq) was added in one portion to a solution of the alcohol **S8** (800 mg, 3.96 mmol, 1.0 eq) in DMSO (20 mL) and the reaction stirred for 2 h. The reaction was then diluted with H_2O (30 mL) and filtered to remove the white precipitate, rinsing with ethyl acetate (30 mL x 2). The layers were separated, and the aqueous layer was extracted with ethyl acetate (3 x 10 mL). The combined organic layers were then dried over Na_2SO_4, filtered, and concentrated in vacuo. Purification via flash chromatography (10% Et_2O/Hexanes) afforded the product **S9** (781 mg, 3.90 mmol) in 98% yield as a colorless oil.
$[\alpha]_D^{20}$ = -19.9 (c 1.0, $CHCl_3$); **^{1}H NMR** (500 MHz, $CDCl_3$): δ 9.54 (s, 1H), 3.85 (dddd, *J* = 11.1, 7.6, 5.4, 2.1 Hz, 1H), 3.71 (s, 3H), 2.64 (dd, *J* = 15.5, 7.6 Hz, 1H), 2.48 (dd, *J* = 15.4, 5.4 Hz, 1H), 1.93 (dq, *J* = 13.1, 3.3 Hz, 2H), 1.76 – 1.69 (m, 2H), 1.61 (dddd, *J* = 12.8, 11.4, 6.6, 4.0 Hz, 1H), 1.41 (tdd, *J* = 12.1, 10.7, 3.2 Hz, 2H), 1.37 – 1.28 (m, 2H), 0.94 (d, *J* = 6.6 Hz, 3H); **^{13}C NMR** (125 MHz, $CDCl_3$): δ 200.7, 171.5, 86.7, 73.6, 51.8, 41.0, 32.1, 30.8, 30.6, 16.4; **HRMS-ESI** (m/z): calcd. for $C_{10}H_{16}NaO_4$ [M + Na]+: 203.0941, found 203.0941; **TLC**: R_f = 0.45 with Hex:EtOAc (7:3). p-anisaldehyde (blue spot). Isolated as a clear oil.

methyl 2-((2*R*,5*S*,6*S*)-6-ethynyl-5-methyltetrahydro-2*H*-pyran-2-yl)acetate (S10): K_2CO_3 (124 mg, 0.90 mmol, 3.0 eq) was added to a stirred solution of aldehyde **S9** (60 mg, 0.30 mmol, 1.0 eq) and Ohira-Bestmann reagent (144 mg, 0.75 mmol, 2.5 eq) in MeOH (1.5 mL, 0.2 M) at room temperature and the flask was wrapped in aluminum foil. The reaction was stirred for 4h (Followed by TLC), then diluted with Et_2O (5 mL), and quenched with Na_2CO_3 sat. (10 mL) and extracted with Et_2O (3 x 10 mL). The combined organic layers were then dried over Na_2SO_4, filtered, and concentrated in vacuo. Purification via flash chromatography (5% EtOAc/Hexanes) afforded the product **S10** (54 mg, 0.28 mmol) in 92% yield as a colorless oil.
$[\alpha]_D^{20}$ = -6.8 (c 1.7, $CHCl_3$); **^{1}H NMR** (500 MHz, $CDCl_3$): δ 3.79 – 3.75 (m, 1H), 3.75 – 3.72 (m, 1H), 3.67 (d, *J* = 1.0 Hz, 3H), 2.66 (ddd, *J* = 15.6, 6.8, 1.1 Hz, 1H), 2.47 – 2.46 (m, 1H), 2.41 (ddd, *J* = 15.7, 6.4, 0.9 Hz, 1H), 1.89 – 1.81 (m, 1H), 1.73 – 1.66 (m, 1H), 1.66 – 1.58 (m, 1H), 1.42 – 1.29 (m, 1H), 1.27 – 1.16 (m, 1H), 0.97 (dd, *J* = 6.7, 0.9 Hz, 3H); **^{13}C NMR** (125 MHz, $CDCl_3$): δ 171.6, 82.4, 74.6, 74.4, 73.4, 51.8, 41.0, 36.1, 31.8, 31.1, 17.8; **HRMS-ESI** (m/z): calcd. for $C_{11}H_{16}NaO_3$ [M + Na]+: 219.0992, found 219.0996; **TLC**: R_f = 0.43 with Hex:EtOAc (4:1). p-anisaldehyde (black spot). Isolated as a clear oil.

methyl 2-((2*R*,5*S*,6*S*)-6-acetyl-5-methyltetrahydro-2*H*-pyran-2-yl)acetate (S11): A solution of $HgSO_4$ (16 mg, 0.06 mmol, 0.2 eq) in aqueous H_2SO_4 (0.4 mL, 3 M) was added to a solution of the alkyne **S10** (54 mg, 0.28 mmol, 1.0 eq) in THF (1.4 mL) at room temperature and stirred for 2 h. The mixture was diluted with Et_2O (5 mL) and carefully neutralized with Na_2CO_3 (solid) until bubbling ceased. Water (10 mL) was added and extracted with Et_2O (3 x 10 mL). The combined organic layers were dried over Na_2SO_4, filtered, and concentrated in vacuo. Purification via flash chromatography (3% EtOAc/Hexanes) afforded the product **S11** (32 mg, 0.15 mmol) in 53% yield as a colorless oil.
$[\alpha]_D^{20}$ = -89.6 (c 0.7, $CHCl_3$); **^{1}H NMR** (500 MHz, $CDCl_3$): δ 3.81 (dddd, *J* = 11.2, 7.6, 5.3, 2.2 Hz, 1H), 3.70 (s, 4H), 3.44 (d, *J* = 10.3 Hz, 1H), 2.60 (dd, *J* = 15.1, 7.7 Hz, 1H), 2.48 (dd, *J* = 15.2, 5.3 Hz, 1H), 2.17 (s, 3H), 1.96 – 1.84 (m, 1H), 1.77 – 1.69 (m, 1H), 1.56 (dddt, *J* = 13.1, 7.6, 6.5, 3.3 Hz, 1H), 1.43 (tdd, *J* = 13.0, 11.1, 3.8 Hz, 1H), 1.30 (tdd, *J* = 13.4, 11.7, 5.6 Hz, 1H), 0.85 (d, *J* = 6.6 Hz,

3H); ^{13}C **NMR** (125 MHz, $CDCl_3$): δ 208.0, 171.7, 89.2, 73.9, 51.8, 41.3, 32.4, 32.0, 31.2, 26.0, 17.0; **HRMS-ESI** (m/z): calcd. for $C_{11}H_{19}O_4$ [M + H]+: 215.1278, found 215.1282; **TLC**: R_f = 0.62 with Hex:EtOAc (1:1). p-anisaldehyde (black spot). Isolated as a clear oil.

methyl 2-((2*R*,5*S*,6*S*)-6-((*E*)-1-iodoprop-1-en-2-yl)-5-methyltetrahydro-2*H*-pyran-2-yl)acetate (S12): A solution of ketone **S11** (50 mg, 0.24 mmol, 1.0 eq) and iodoform (280 mg, 0.71 mmol, 3.0 eq) in a THF/dioxane mix (4:1, 1 mL) was added via cannula to a rapidly stirred slurry of chromium (II) chloride (295 mg, 2.40 mmol, 10.0 eq) in a THF/dioxane mix (4:1, 1.5 mL) at 0 ˚C. The reaction was stirred for 1.5 h, then warmed to room temperature, poured into water (10 mL), and extracted with Et_2O (1 x 10 mL). The combined organic layers were then dried over Na_2SO_4, filtered, and concentrated in vacuo. Purification via flash chromatography (3% EtOAc/Hexanes) afforded the product **S12** (42 mg, 0.12 mmol) in 51% yield as a colorless oil.
[α]$_D^{20}$ = +9.9 (c 1.2, $CHCl_3$); ^{1}H **NMR** (500 MHz, $CDCl_3$): δ 6.22 (dt, *J* = 1.5, 0.7 Hz, 1H), 3.80 (dtd, *J* = 11.1, 6.4, 2.2 Hz, 1H), 3.69 (s, 3H), 3.53 (d, *J* = 9.8 Hz, 1H), 2.59 (dd, *J* = 15.3, 6.6 Hz, 1H), 2.42 (dd, *J* = 15.3, 6.3 Hz, 1H), 1.91 – 1.82 (m, 1H), 1.81 (d, *J* = 1.1 Hz, 3H), 1.73 – 1.68 (m, 1H), 1.59 – 1.48 (m, 1H), 1.37 (tdd, *J* = 12.8, 11.1, 3.6 Hz, 1H), 1.27 (ddd, *J* = 13.1, 11.5, 3.7 Hz, 1H), 0.72 (d, *J* = 6.6 Hz, 3H); ^{13}C **NMR** (125 MHz, $CDCl_3$): δ 171.7, 147.1, 89.0, 80.3, 74.1, 51.6, 41.1, 32.6, 32.2, 31.5, 19.5, 17.5; **HRMS-ESI** (m/z): calcd. for $C_{12}H_{20}IO_3$ [M + H]+: 339.0452, found 339.0450; **TLC**: R_f = 0.52 with Hex:EtOAc (4:1). p-anisaldehyde (black spot). Isolated as a clear oil.

methyl 2-((2R,5S,6S)-6-((E)-2-iodovinyl)-5-methyltetrahydro-2H-pyran-2-yl)acetate (S13): A solution of aldehyde **S9** (100 mg, 0.50 mmol, 1.0 eq) and iodoform (433 mg, 1.1 mmol, 2.2 eq) in a THF/dioxane mix (4:1, 2 mL) was added via cannula to a rapidly stirred slurry of chromium (II) chloride (369 mg, 3.0 mmol, 6.0 eq) in a THF/dioxane mix (4:1, 6 mL) at 0 ˚C. The reaction was stirred for 1.5 h, then warmed to room temperature, poured into water (30 mL), and extracted with ether (1 x 30 mL). The combined organic layers were then dried over Na_2SO_4, filtered, and concentrated in vacuo. Purification via flash chromatography (3% EtOAc/Hexanes) afforded the product **S13** (73 mg, 0.21 mmol) in 42% yield as a colorless oil.
[α]$_D^{20}$ = -19.9 (c 1.0, $CHCl_3$); ^{1}H **NMR** (500 MHz, $CDCl_3$): δ 6.53 (dd, *J* = 14.4, 6.7 Hz, 1H), 6.32 (dd, *J* = 14.4, 1.1 Hz, 1H), 3.77 (dddd, *J* = 11.1, 7.7, 5.8, 2.1 Hz, 1H), 3.68 (s, 3H), 3.41 (ddd, *J* = 9.8, 6.8, 1.1 Hz, 1H), 2.57 (dd, *J* = 15.3, 7.3 Hz, 1H), 2.40 (dd, *J* = 15.2, 5.9 Hz, 1H), 1.82 (dq, *J* = 13.2, 3.4 Hz, 1H), 1.70 – 1.63 (m, 1H), 1.40 – 1.29 (m, 2H), 1.28 – 1.20 (m, 1H), 0.82 (d, *J* = 6.6 Hz, 3H); ^{13}C **NMR** (125 MHz, $CDCl_3$): δ 171.8, 145.2, 86.0, 79.1, 74.2, 51.8, 41.3, 35.0, 32.3, 31.5, 17.6; **HRMS-ESI** (m/z): calcd. for $C_{11}H_{18}IO_3$ [M + H]+: 325.0295, found 325.0299; **TLC**: R_f = 0.33 with Hex:EtOAc (4:1). p-anisaldehyde (blue spot). Isolated as a clear oil.

Fragment B

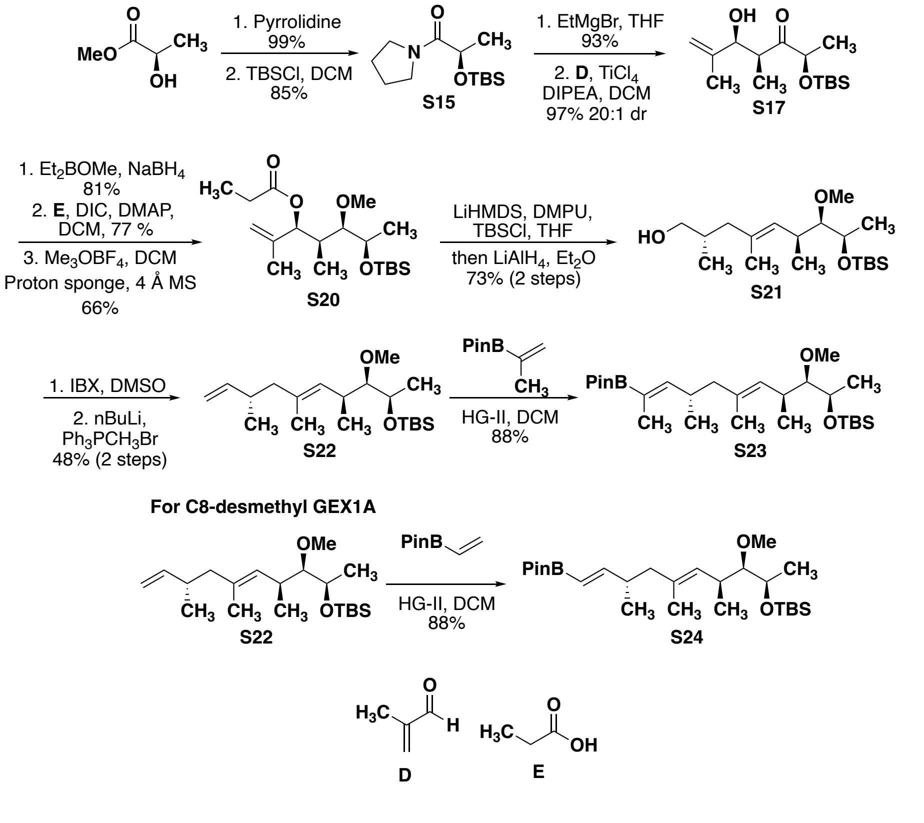

Scheme 2. Synthetic sequence for GEX1A (Fragment B).

(*R*)-2-hydroxy-1-(pyrrolidin-1-yl)propan-1-one (S14): Methyl-(R)-(+)-lactate (19.3 mL, 170 mmol, 1.0 eq) was added to pyrrolidine (15.5 mL, 190 mmol, 1.1 eq) at 0 ˚C. The mixture was stirred for 15 min at 0 ˚C and for 3 days at rt. After the removal of the volatiles under reduced pressure to afford pure amide **S14** as a brown oil. Used without further purification.
$[\alpha]_D^{20}$ = +51.7 (c 1.0, $CHCl_3$); **^{1}H NMR** (500 MHz, $CDCl_3$): δ 4.28 (p, *J* = 6.3 Hz, 1H), 3.56 (dt, *J* = 11.9, 7.4 Hz, 1H), 3.47 (dtd, *J* = 10.8, 5.6, 2.1 Hz, 2H), 3.35 – 3.28 (m, 1H), 2.03 – 1.93 (m, 1H), 1.93 – 1.82 (m, 1H), 1.33 (d, *J* = 6.6 Hz, 1H); **^{13}C NMR** (125 MHz, $CDCl_3$): δ 173.5, 65.5, 46.3, 46.0, 26.1, 26.1, 23.9, 23.9, 20.7, 20.6; **TLC**: R_f = 0.24 with EtOAc. $KMnO_4$ (yellow spot). Isolated as a clear oil.

(*R*)-2-((*tert*-butyldimethylsilyl)oxy)-1-(pyrrolidin-1-yl)propan-1-one (S15): From S15 to S22, prepared according to reported procedures. [6] Amide **S14** (24.3 g, 170 mmol, 1.0 eq) was added dropwise to a solution of imidazole (23.1 g, 340 mmol, 2.0 eq) in DCM (200 ml) at 0 °C. Subsequently, TBSCl (38.4 g, 255 mmol) in DCM (150 mL) was added slowly and the reaction mixture was stirred at room temperature overnight. The mixture was then diluted with H_2O (200 ml) and extracted with DCM (3 x 100 mL). The organic extracts were combined, dried over Na_2SO_4, filtered, and concentrated to obtain a yellow oil. Purification via flash chromatography (0 to 30% EtOAc/Hexanes) afforded the product **S15** (37.2 g, 145 mmol) in 85% yield as a colorless oil.
$[\alpha]_D^{20}$ = +19.5 (c 1.0, $CHCl_3$); **^{1}H NMR** (500 MHz, $CDCl_3$): δ 4.48 – 4.42 (m, 1H), 3.66 – 3.59 (m, 1H), 3.59 – 3.53 (m, 1H), 3.47 (ddt, *J* = 7.2, 5.1, 2.7 Hz, 2H), 1.94 – 1.86 (m, 2H), 1.85 – 1.75 (m, 2H), 1.35 (d, *J* = 6.7 Hz, 3H), 0.88 (s, 9H), 0.07 (s, 6H); **^{13}C NMR** (125 MHz, $CDCl_3$): δ 172.1, 70.7, 46.4, 46.2, 26.5, 25.8, 23.6, 20.7, 18.2, -4.8, -4.9; **TLC**: R_f = 0.50 with Hex:EtOAc (1:1). $KMnO_4$ (yellow spot). Isolated as a clear oil.

(*R*)-2-((*tert*-butyldimethylsilyl)oxy)pentan-3-one (S16): Amide **S15** (16.78 g, 65.2 mmol, 1.0 eq) was dissolved in dry THF (217 mL, 0.3 M) and cooled to -30 °C. Ethylmagnesium bromide (28 mL, 72.0 mmol, 1.1 eq, 2.6 M in Et_2O) was slowly added over 30 minutes. The mixture was allowed to warm to room temperature and stirred for 1h. The reaction was terminated by the addition of NH_4Cl saturated solution and extracted with Et_2O (3 x 30 mL). The combined organic layers were then dried over Na_2SO_4, filtered, and concentrated in vacuo. Purification via flash chromatography (10% Et_2O/Pentanes) afforded the product **S16** (13.1 g, 60.6 mmol) in 93% yield as a colorless oil. Caution: The product is volatile! Don't leave it under reduced pressure for long periods of time.
$[\alpha]_D^{20}$ = +8.2 (c 1.0, $CHCl_3$); **^{1}H NMR** (500 MHz, $CDCl_3$): δ 4.15 (q, *J* = 6.8 Hz, 1H), 2.64 (dq, *J* = 18.7, 7.3 Hz, 1H), 2.56 (dq, *J* = 18.7, 7.3 Hz, 1H), 1.27 (d, *J* = 6.8 Hz, 3H), 1.03 (t, *J* = 7.3 Hz, 3H), 0.91 (s, 9H), 0.07 (s, 6H); **^{13}C NMR** (125 MHz, $CDCl_3$): δ 215.1, 74.8, 30.2, 25.7, 21.0, 18.1, 7.3, -4.7, -5.1; **TLC**: R_f = 0.75 with Hex:EtOAc (1:1). p-anisaldehyde (black spot). Isolated as a clear oil.

(2*R*,4*S*,5*S*)-2-((*tert*-butyldimethylsilyl)oxy)-5-hydroxy-4,6-dimethylhept-6-en-3-one (S17): Neat $TiCl_4$ (3.34 mL, 30.5 mmol, 1.1 eq) was added to the ketone **S15** (6.00 g, 27.7 mmol, 1.0 eq) solution in DCM (80 mL, 0.3 M) at -78 °C. The resulting solution was stirred for 5 min and was then treated with freshly distilled DIPEA (5.31 mL, 30.5 mmol, 1.1 eq) by dropwise addition. The reaction mixture was stirred for 30 min at -78 °C followed by the dropwise addition of freshly distilled methacrolein (3.44 mL, 41.6 mmol, 1.5 eq). The reaction mixture was then stirred for 30 min at -78 °C and then allowed to warm to room temperature before adding a saturated solution of Rochelle's salt (100 ml) and extracted with DCM (1 x 50 mL) once the two layers were clearly separated. The combined organic layers were dried over Na_2SO_4, filtered, and concentrated in vacuo. Purification via flash chromatography (25% EtOAc/Hexanes) afforded the product **S17** (7.63 g, 26.6 mmol) in 97% yield as a colorless oil.
$[\alpha]_D^{20}$ = -5.1 (c 1.0, $CHCl_3$); **^{1}H NMR** (500 MHz, $CDCl_3$): δ 5.12 (dp, *J* = 2.4, 0.8 Hz, 1H), 4.96 (dtt, *J* = 2.4, 1.6, 0.8 Hz, 1H), 4.34 (dtd, *J* = 2.8, 1.4, 0.7 Hz, 1H), 4.26 – 4.20 (m, 1H), 3.32 (tddd, *J* = 7.2, 6.3, 2.7, 0.6 Hz, 1H), 1.68 (dq, *J* = 1.5, 0.7 Hz, 3H), 1.35 (dd, *J* = 6.9, 0.6 Hz, 3H), 1.07 (dd, *J* = 7.2, 0.7 Hz, 3H), 0.92 (d, *J* = 0.8 Hz, 9H), 0.11 – 0.08 (m, 6H); **^{13}C NMR** (125 MHz, $CDCl_3$): δ 219.5, 143.3, 111.9, 74.8, 73.4, 41.9, 25.8, 21.5, 19.8, 18.2, 9.7, -4.5, -4.9; **HRMS-ESI** (m/z): calcd. for $C_{15}H_{30}NaO_3Si$ [M + Na]+: 309.1856, found 309.1850; **TLC**: R_f = 0.68 with Hex:EtOAc (1:1). p-anisaldehyde (brown spot). Isolated as a clear oil.

3*S*,4*S*,5*R*,6*R*)-6-((*tert*-butyldimethylsilyl)oxy)-2,4-dimethylhept-1-ene-3,5-diol (S18): Et_2BOMe (20.9 mL, 20.9 mmol, 1.2 eq, 1.0 M in THF) was added to a solution of keto-alcohol **S17** (5.00 g, 17.4 mmol, 1.0 eq) in THF (65 mL) and MeOH (17 mL) at -40 °C under Ar. This mixture was stirred at -40 °C for 20 min. $NaBH_4$ (10.5 mL, 20.9 mmol, 1.2 eq, 2.0 M in THF) was slowly added, then the reaction mixture was stirred and warmed to room temperature for 12 h. Quenched with glacial AcOH (6 mL) and vigorously stirred at room temperature. The mixture was partitioned with EtOAc and H_2O, the aqueous layer was extracted with EtOAc (3 x 30), and the combined organic extracts were washed with 1 M NaOH and dried over Na_2SO_4. The volatiles were removed in vacuo and the resulting oil was treated with 33% w/v H_2O_2 (15 mL) in 1 M NaOAc in 9:1 MeOH/H_2O (100 mL) for 1h at rt. The solution was extracted with EtOAc (3 x 30 mL), and the combined organic extracts were dried over Na_2SO_4, filtered, and concentrated in vacuo. Purification via flash chromatography (5% Et_2O/Hexanes) afforded the product **S18** (3.02 g, 10.5 mmol) in 60% yield as a colorless oil, that solidified upon cooling.
$[\alpha]_D^{20}$ = -8.1 (c 1.0, $CHCl_3$); **^{1}H NMR** (500 MHz, $CDCl_3$): δ 5.10 (ddt, *J* = 2.2, 1.5, 0.8 Hz, 1H), 4.93 (dp, *J* = 2.8, 1.5 Hz, 1H), 4.25 – 4.22 (m, 1H), 3.77 (dq, *J* = 7.8, 6.1 Hz, 1H), 3.50 (dd, *J* = 7.8, 2.4 Hz, 1H), 1.70 – 1.67 (m, 3H), 1.13 (d, *J* = 6.1 Hz, 3H), 0.91 (s, 9H), 0.84 (d, *J* = 7.1 Hz, 3H), 0.11 (d, *J* = 1.4 Hz, 5H); **^{13}C NMR** (125 MHz, $CDCl_3$): δ 144.9, 111.3, 80.6, 79.0, 70.5, 35.9, 26.0, 20.0, 19.7, 18.2, 5.4, -3.9, -4.6; **HRMS-ESI** (m/z): calcd. for $C_{15}H_{32}NaO_3Si$ [M + Na]+: 311.2013, found 311.2020; **TLC**: R_f = 0.45 with Hex:Et_2O (3:2). p-anisaldehyde (blue spot). Isolated as a clear oil.

(3*S*,4*R*,5*R*,6*R*)-6-((*tert*-butyldimethylsilyl)oxy)-5-hydroxy-2,4-dimethylhept-1-en-3-yl propionate (S19): DIC (2.32 mL, 15.0 mmol, 1.2 eq) was added over 10 min to a solution of diol **S18** (3.60 g, 12.5 mmol, 1.0 eq), DMAP (24 mg, 0.2 mmol, 0.02 eq) and propionic

acid (1.03 mL, 13.8 mmol, 1.1 eq) in DCM (125 mL, 0.1 M) at 0 °C under Ar. The mixture was stirred overnight and filtered through Celite with DCM (3 x 30 mL). Silica gel was added, and the mixture was concentrated under reduced pressure. Purification via flash chromatography (5% Et_2O/Hexanes) afforded the product **S19** (3.30 g, 9.58 mmol) in 76% yield as a colorless oil. Starting material (1.36 g) was recovered.
$[\alpha]_D^{20}$ = -5.7 (c 1.0, $CHCl_3$); **^{1}H NMR** (500 MHz, $CDCl_3$): δ 5.26 (d, *J* = 7.5 Hz, 1H), 5.02 (dt, *J* = 1.5, 0.8 Hz, 1H), 4.97 (t, *J* = 1.6 Hz, 1H), 3.81 (p, *J* = 6.1 Hz, 1H), 3.18 (dd, *J* = 6.2, 3.9 Hz, 1H), 2.34 (qd, *J* = 7.6, 1.1 Hz, 2H), 1.90 – 1.81 (m, 1H), 1.70 (t, *J* = 1.1 Hz, 3H), 1.15 (t, *J* = 7.6 Hz, 3H), 1.12 (d, *J* = 6.1 Hz, 3H), 0.93 (d, *J* = 6.8 Hz, 3H), 0.90 (s, 9H), 0.10 (d, *J* = 0.7 Hz, 6H); **^{13}C NMR** (125 MHz, $CDCl_3$): δ 173.7, 141.9, 114.5, 79.0, 75.8, 69.8, 36.6, 28.0, 26.0, 26.0, 20.4, 18.6, 18.2, 9.3, 9.3, -3.9, -4.6; **HRMS-ESI** (m/z): calcd. for $C_{18}H_{36}NaO_4Si$ [M + Na]+: 367.2275, found 367.2252; **TLC**: R_f = 0.27 with Hex:Et_2O (9:1). p-anisaldehyde (brown spot). Isolated as a clear oil.

(3*S*,4*S*,5*R*,6*R*)-6-((*tert*-butyldimethylsilyl)oxy)-5-methoxy-2,4-dimethylhept-1-en-3-yl propionate (S20): A hydroxyl ester **S19** (3.30 g, 9.56 mmol, 1.0 eq) solution in DCM (96 mL) was treated with 4 Å molecular sieves (3.65 g), proton-sponge (6.56 g, 30.6 mmol, 3.2 eq) and Me_3OBF_4 (4.24 g, 28.7 mmol, 3.0 eq). The reaction mixture was allowed to stir at room temperature for 3 h and then treated with a second portion of 4 Å molecular sieves (3.65 g), proton-sponge (6.56 g, 30.6 mmol, 3.2 eq), and Me_3OBF_4 (4.24 g, 28.7 mmol, 3.0 eq). The reaction was stirred for 3 h, filtered through Celite, eluted with DCM (3x100 ml), and the filtrates were concentrated under vacuum. The residue was dissolved in Et_2O (70 mL), washed with 1 M HCl (3×30 mL), brine (30 mL), dried over Na_2SO_4, and concentrated under vacuum. Purification via flash chromatography (2% Et_2O/Hexanes) afforded the product **S20** (2.39 g, 6.94 mmol) in 70% yield as a colorless oil.
$[\alpha]_D^{20}$ = -18.8 (c 1.0, $CHCl_3$); **^{1}H NMR** (500 MHz, $CDCl_3$): δ 5.25 (dd, *J* = 8.0, 0.9 Hz, 1H), 5.02 (dq, *J* = 1.6, 0.8 Hz, 1H), 5.00 (q, *J* = 1.6 Hz, 1H), 3.95 – 3.86 (m, 1H), 3.43 (d, *J* = 1.1 Hz, 3H), 2.94 – 2.89 (m, 1H), 2.38 – 2.30 (m, 3H), 2.02 (dddd, *J* = 8.0, 6.9, 2.6, 1.1 Hz, 1H), 1.70 (dd, *J* = 1.5, 0.9 Hz, 4H), 1.14 (t, *J* = 7.6 Hz, 2H), 1.10 (d, *J* = 6.3 Hz, 1H), 0.90 (s, 6H), 0.08 (d, *J* = 1.8 Hz, 6H); **^{13}C NMR** (125 MHz, $CDCl_3$): δ 173.7, 142.1, 115.7, 85.1, 80.0, 70.4, 60.7, 35.7, 28.0, 26.1, 20.2, 18.3, 18.2, 9.7, 9.3, -4.4, -4.4; **HRMS-ESI** (m/z): calcd. for $C_{19}H_{39}O_4Si$ [M + H]+: 359.2612, found 359.2611; **TLC**: R_f = 0.40 with Hex:Et_2O (9:1). p-anisaldehyde (blue spot). Isolated as a clear oil.

(2*S*,6*S*,7*R*,8*R*,*E*)-8-((*tert*-butyldimethylsilyl)oxy)-7-methoxy-2,4,6-trimethylnon-4-en-1-ol (S21): DMPU (3.5 mL, THF:DMPU (4:1)) was added dropwise to a solution of LiHMDS (7.0 mL, 7.0 mmol, 5 eq, 1.0 M in THF) in THF (9 mL) at -78 °C and stirred for 5 min. Then a solution of the ester **S20** (0.500 g, 1.39 mmol, 1 eq) and TBSCl (1.21 g, 8.1 mmol, 5.8 eq) in THF (5 mL) was added dropwise and the reaction was stirred for 45 min at -78 °C. The reaction was quenched at -78 °C by the addition of MeOH (2 mL), warmed to room temperature, and stirred for 30 min. The mixture was partitioned with Et_2O (15 mL) and 1 M HCl (20 mL). The organic layer was washed with 1 M HCl (20 mL), dried over Na_2SO_4, and concentrated to obtain the crude product. The previous crude mixture was dissolved in dry Et_2O (1.4 mL) and cooled to -40 °C while stirring. Then $LiAlH_4$ (2.1 mL, 4.2 mmol, 3.0 eq, 2.0 M in THF). The reaction mixture was warmed to 0 °C and stirred for 2 h before being quenched by the portion-wise addition of $Na_2SO_4 \cdot 10H_2O$ as a solid, and then stirred overnight. The precipitate that formed was filtered through Celite and washed with Et_2O (2 x 50 mL). The organic layer was dried over Na_2SO_4 and concentrated under vacuum. Purification via flash chromatography (15% EtOAc/Hexanes) afforded the product **S21** (0.402 g, 1.2 mmol) in 84% (both reactions) yield as a colorless oil.
$[\alpha]_D^{20}$ = +8.3 (c 1.0, $CHCl_3$); **^{1}H NMR** (500 MHz, $CDCl_3$): δ 5.19 (dq, *J* = 9.3, 1.2 Hz, 1H), 3.87 – 3.81 (m, 1H), 3.50 (dd, *J* = 10.6, 5.7 Hz, 1H), 3.44 (s, 3H), 3.44 – 3.40 (m, 1H), 2.79 (dd, *J* = 5.9, 5.0 Hz, 1H), 2.61 – 2.52 (m, 1H), 2.06 (ddd, *J* = 12.6, 6.4, 1.2 Hz, 1H), 1.91 – 1.84 (m, 1H), 1.81 (ddd, *J* = 12.7, 7.7, 1.1 Hz, 1H), 1.62 (d, *J* = 1.4 Hz, 3H), 1.12 (d, *J* = 6.3 Hz, 3H), 0.92 (d, *J* = 6.8 Hz, 3H), 0.89 (s, 9H), 0.88 (d, *J* = 6.5 Hz, 3H), 0.06 (s, 6H); **^{13}C NMR** (125 MHz, $CDCl_3$): δ 132.2, 131.0, 89.9, 70.2, 68.7, 61.0, 44.6, 33.9, 33.9, 26.1, 20.3, 18.3, 17.0, 16.2, 15.6, -4.4, -4.5; **HRMS-ESI** (m/z): calcd. for $C_{19}H_{41}O_3Si$ [M + H]+: 345.2819, found 345.2812; **TLC**: R_f = 0.55 with Hex:EtOAc (4:1). p-anisaldehyde (mangenta spot). Isolated as a clear oil.

***tert*-butyl(((2*R*,3*R*,4*S*,8*S*,*E*)-3-methoxy-4,6,8-trimethyldeca-5,9-dien-2-yl)oxy)dimethylsilane (S22):** Prepared according to reported procedures. [7] IBX (1.35 g, 4.81 mmol, 3.0 eq) was added in one portion to a solution of the alcohol **S21** (553 mg, 1.60 mmol, 1.0 eq) in DMSO (8 mL, 0.2 M) and the reaction stirred for 2 h at room temperature. The solution was diluted with H_2O (30 mL), filtered through Celite, and rinsed with EtOAc (2 x 15 mL). The layers were separated, and the aqueous layer was extracted with EtOAc (3 x 10 mL). The combined organic layers were dried over Na_2SO_4, filtered, and concentrated in vacuo. The crude material was taken on to the next step without further purification. KOtBu (2.0 mL, 3.20 mmol, 2.0 eq, 1.6 M in THF) was added dropwise to a solution of (Methyl)triphenylphosphonium bromide (1.43 g, 4.00 mmol, 2.5 eq) in THF (6 mL) at room temperature and stirred for 1 h under Ar. The reaction was cooled to –78 °C, followed by the dropwise addition of the crude aldehyde in THF (2 mL). The reaction mixture was allowed to warm slowly to room temperature overnight. The reaction was quenched with sat. $NaHCO_3$ (aq) (15 mL) and extracted with EtOAc (3 x 10 mL). The combined organic layers were washed with brine, dried over Na_2SO_4, filtered, and concentrated in vacuo. Purification via flash chromatography (5% Et_2O/Hexanes) afforded the product **S22** (461 mg, 1.35 mmol) in 84% yield as a colorless oil.
$[\alpha]_D^{20}$ = +16.8 (c 1.0, $CHCl_3$); **^{1}H NMR** (500 MHz, $CDCl_3$): δ 5.72 (ddd, *J* = 17.3, 10.3, 7.1 Hz, 1H), 5.14 (dd, *J* = 9.3, 1.3 Hz, 1H), 4.94 (dt, *J* = 17.2, 1.6 Hz, 1H), 4.88 (ddt, *J* = 10.3, 1.7, 0.8 Hz, 1H), 3.44 (d, *J* = 0.5 Hz, 3H), 2.79 (dd, *J* = 5.9, 4.9 Hz, 1H), 2.55 (dqd, *J* = 9.4, 6.8, 4.9 Hz, 1H), 2.32 (dq, *J* = 8.1, 6.5 Hz, 1H), 2.01 (ddd, *J* = 13.5, 7.1, 1.2 Hz, 1H), 1.89 (ddd, *J* = 13.5, 7.8, 1.0 Hz, 1H), 1.59 (d, *J* = 1.4 Hz, 3H), 1.13 (dd, *J* = 6.4, 0.5 Hz, 3H), 0.95 (d, *J* = 6.7 Hz, 2H), 0.91 (d, *J* = 6.8 Hz, 2H), 0.89 (d, *J* = 0.6 Hz, 9H), 0.06 (d, *J* = 0.5 Hz, 6H); **^{13}C NMR** (125 MHz, $CDCl_3$): δ 144.8, 131.7, 131.0, 112.1, 90.0, 70.3, 61.0, 47.4, 35.8, 33.9, 26.1, 20.3, 19.7, 18.3, 16.3,

15.6, -4.4, -4.4; **HRMS-ESI** (m/z): calcd. for $C_{19}H_{38}NaO_3Si$ [M + Na]+: 365.2482, found 365.2479; **TLC**: R_f = 0.30 with Hex:Et_2O (9:1). p-anisaldehyde (blue spot). Isolated as a clear oil.

***tert*-butyl(((2*R*,3*R*,4*S*,5*E*,8*S*,9*Z*)-3-methoxy-4,6,8-trimethyl-10-(4,4,5,5-tetramethyl-1,3,2-dioxaborolan-2-yl)undeca-5,9-dien-2-yl)oxy)dimethylsilane (S23):** Prepared according to reported procedures. [7] To a solution of olefin **S22** (50 mg, 0.15 mmol, 1.0 eq) and isopropenylboronic acid pinacol ester (0.3 mL, 1.5 mmol, 10.0 eq) in DCM (1.5 mL, degassed with Ar for 15 min) was added Hoveyda-Grubbs 2nd generation catalyst (9 mg, 0.02 mmol, 0.1 eq). The resulting solution was allowed to stir at 50 °C for 24 h in a sealed pressure vial. The reaction was quenched by adding imidazole (10 mg, to precipitate the catalyst) and stirred for 30 min. The mixture was filtered through a short plug of silica gel and eluted with DCM (3 × 10 mL). The filtrate was concentrated in vacuo to provide a brown oil. Purification via flash chromatography (2% Et_2O/Hexanes) afforded the product **S23** (63 mg, 0.13 mmol) in 88% yield as a colorless oil.
$[\alpha]_D^{20}$ = +4.7 (c 1.1, $CHCl_3$); **^{1}H NMR** (500 MHz, $CDCl_3$): δ 6.07 (dd, *J* = 9.3, 1.8 Hz, 1H), 5.16 – 5.03 (m, 1H), 3.81 (p, *J* = 6.3 Hz, 1H), 3.43 (s, 3H), 2.77 (dd, *J* = 5.9, 4.9 Hz, 1H), 2.75 – 2.68 (m, 1H), 2.52 (dqd, *J* = 9.2, 6.7, 4.9 Hz, 1H), 1.97 (ddd, *J* = 13.4, 7.3, 1.2 Hz, 1H), 1.90 (ddd, *J* = 13.3, 7.4, 1.0 Hz, 1H), 1.67 (d, *J* = 1.7 Hz, 3H), 1.57 (d, *J* = 1.3 Hz, 3H), 1.25 (s, 6H), 1.25 (s, 6H), 1.12 (d, *J* = 6.4 Hz, 3H), 0.91 (d, *J* = 6.6 Hz, 3H), 0.89 (s, 9H), 0.88 – 0.87 (m, 3H), 0.06 (s, 6H); **^{13}C NMR** (125 MHz, $CDCl_3$): δ 152.4, 131.7, 130.8, 90.0, 83.2, 70.3, 61.0, 47.2, 33.9, 31.1, 26.1, 25.0, 24.9, 20.4, 19.9, 18.3, 16.3, 15.6, 14.0, -4.4, -4.4; **HRMS-ESI** (m/z): calcd. for $C_{26}H_{51}BNaO_4Si$ [M + Na]+: 489.3542, found 489.3534; **TLC**: R_f = 0.64 with Hex:EtOAc (4:1). p-anisaldehyde (blue spot). Isolated as a yellowish oil.

***tert*-butyl(((2*R*,3*R*,4*S*,5*E*,8*S*,9*E*)-3-methoxy-4,6,8-trimethyl-10-(4,4,5,5-tetramethyl-1,3,2-dioxaborolan-2-yl)deca-5,9-dien-2-yl)oxy)dimethylsilane (S24):** To a solution of olefin **S22** (50 mg, 0.15 mmol, 1.0 eq) and vinyl boronic acid pinacol ester (30 µL, 0.18 mmol, 1.2 eq) in DCM (1.9 mL, degassed with Ar for 15 min) was added Hoveyda-Grubbs 2nd generation catalyst (19 mg, 0.03 mmol, 0.2 eq). The resulting solution was allowed to stir at 50 °C for 36 h in a sealed pressure vial. The reaction was quenched by adding imidazole (20 mg, to precipitate the catalyst) and stirred for 30 min. The mixture was filtered through a short plug of silica gel and eluted with DCM. The filtrate was concentrated in vacuo to provide a brown oil. Purification via flash chromatography (2% Et_2O/Hexanes) afforded the product **S24** (38 mg, 0.082 mmol) in 74% yield as a yellowish oil.
$[\alpha]_D^{20}$ = +2.5 (c 1.0, $CHCl_3$); **^{1}H NMR** (500 MHz, $CDCl_3$): δ 6.51 (dd, *J* = 18.0, 7.0 Hz, 1H), 5.35 (dd, *J* = 18.0, 1.2 Hz, 1H), 5.11 (dd, *J* = 9.4, 1.3 Hz, 1H), 3.82 (p, *J* = 6.3 Hz, 1H), 3.43 (d, *J* = 4.9 Hz, 3H), 2.77 (dd, *J* = 5.9, 4.9 Hz, 1H), 2.53 (dqd, *J* = 9.4, 6.8, 4.9 Hz, 1H), 2.43 – 2.34 (m, 1H), 2.05 (ddd, *J* = 13.4, 7.0, 1.2 Hz, 1H), 1.88 (ddd, *J* = 13.3, 7.7, 1.0 Hz, 1H), 1.56 (d, *J* = 1.3 Hz, 3H), 1.37 (d, *J* = 6.0 Hz, 1H), 1.31 (s, 2H), 1.25 (s, 12H), 1.11 (d, *J* = 6.3 Hz, 3H), 0.95 (d, *J* = 6.7 Hz, 3H), 0.89 (d, *J* = 6.2 Hz, 3H), 0.88 (s, 9H), 0.05 (s, 6H); **^{13}C NMR** (125 MHz, $CDCl_3$): δ 159.9, 131.4, 131.1, 89.9, 83.1, 70.3, 61.0, 46.9, 37.6, 33.9, 26.1, 25.0, 24.9, 24.9, 22.3, 20.3, 19.2, 18.3, 16.2, 15.6, -4.4, -4.4; **HRMS-ESI** (m/z): calcd. for $C_{26}H_{51}BNaO_4Si$ [M + Na]+: 489.3542, found 489.3534; **TLC**: R_f = 0.45 with Hex:Et_2O (9:1). p-anisaldehyde (blue spot). Isolated as a yellowish oil.

Fragments A & B coupling

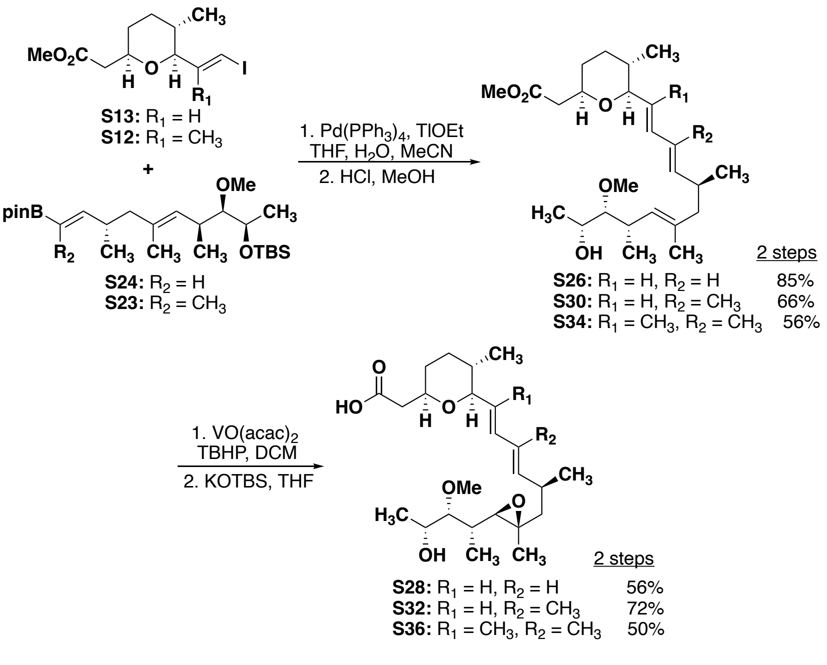

Scheme 3. Coupling of fragments A & B for the synthesis of GEX1A analogs.

methyl 2-((2*R*,5*S*,6*S*) -6-((1*E*,3*E*,5*S*,7*E*,9*S*,10*R*,11*R*) -11-hydroxy- 10-methoxy -5,7,9-trimethyldodeca-1,3,7-trien-1-yl)-5-methyltetrahydro-2*H*-pyran-2-yl) acetate (S26): Prepared according to reported procedures. [8] **Cross-coupling.** $Pd(PPh_3)_4$ (8.0 mg, 6.7 µmol, 0.1 eq) was added to the solution of vinyl boronate **S13** (31 mg, 0.067 mmol, 1.0 eq), and vinyl iodide **S24** (26 mg, 0.080 mmol, 1.2 eq) in THF (1.1 mL)/H_2O (0.4 mL)/CH_3CN (0.18 mL) in a pressure vial, that was sealed and flushed with Ar. After stirring for 5 min, TlOEt (33 mg, 0.134 mmol, 2.0 eq) in THF (0.4 mL) was added and a grey precipitate appeared. The mixture was stirred at 30 °C until the reaction was completed (around 12h). The reaction was diluted with Et_2O (2.5 mL) and a sodium bisulfate saturated solution (1.0 mL) to finalize it. The biphasic mixture was filtered through Celite, and the organic layer was separated, washed with brine, and dried over Na_2SO_4. Purification via flash chromatography (2% Et_2O/Hexanes) afforded the triene product **S25** (28.5 mg, 0.053 mmol) in 79% yield as a colorless oil. R_f = 0.48 with Hex:EtOAc (4:1). p-anisaldehyde (dark spot).

Deprotection. A stirred ice-cooled solution of the triene **S25** (23 mg, 43 µmol) in anhydrous MeOH (0.4 mL, 0.1 M) was slowly treated with freshly prepared methanolic HCl (0.85 mL, 0.17 mmol, 4.0 eq, 0.2 M). The ice bath was removed, and the reaction was allowed to stir for 2 h. The resulting mixture was then quenched with solid $NaHCO_3$ until pH >7. The solution was filtered and washed with EtOAc and concentrated under reduced pressure. Purification via flash chromatography (30% Et_2O/Hexanes) afforded the product **S26** (15.5 mg, 38 µmol) in 85% yield as a colorless oil.

$[\alpha]_D^{20}$ = -8.3 (c 1.3, $CHCl_3$); **^{1}H NMR** (500 MHz, $CDCl_3$): δ 6.14 (dd, *J* = 15.3, 10.4 Hz, 1H), 5.94 (dd, *J* = 15.4, 10.3 Hz, 1H), 5.54 (dd, *J* = 15.2, 7.5 Hz, 1H), 5.49 (dd, *J* = 15.3, 7.6 Hz, 1H), 4.98 – 4.92 (m, 1H), 3.77 (dtd, *J* = 11.1, 6.6, 2.1 Hz, 1H), 3.71 (td, *J* = 6.4, 4.1 Hz, 1H), 3.66 (s, 3H), 3.50 (s, 3H), 3.41 (dd, *J* = 9.7, 7.6 Hz, 1H), 2.69 (dd, *J* = 6.8, 4.1 Hz, 1H), 2.66 – 2.58 (m, 2H), 2.42 – 2.37 (m, 1H), 2.38 (m, 1H), 2.34 (q, *J* = 7.1 Hz, 1H), 1.99 (ddd, *J* = 13.4, 7.3, 1.1 Hz, 1H), 1.90 (ddd, *J* = 13.3, 7.6, 1.0 Hz, 1H), 1.82 (dq, *J* = 13.0, 3.2 Hz, 1H), 1.71 – 1.63 (m, 1H), 1.59 (d, *J* = 1.4 Hz, 3H), 1.37 – 1.31 (m, 2H), 1.24 (s, 2H), 1.18 (d, *J* = 6.3 Hz, 3H), 0.95 (d, *J* = 6.7 Hz, 3H), 0.93 (d, *J* = 6.7 Hz, 3H), 0.77 (d, *J* = 6.6 Hz, 3H); **^{13}C NMR** (125 MHz, $CDCl_3$): δ 172.0, 140.7, 133.3, 133.2, 130.5, 129.8, 127.9, 89.9, 84.9, 74.0, 68.0, 61.6, 51.7, 47.6, 41.4, 35.2, 34.9, 34.8, 32.4, 31.7, 20.6, 20.0, 17.9, 16.7, 16.4; **HRMS-ESI** (m/z): calcd. for $C_{25}H_{43}O_5$ [M + H]+: 423.3105, found 423.3108; **TLC**: R_f = 0.57 with Hex:EtOAc (1:1). p-anisaldehyde (purple spot). Isolated as a clear oil.

methyl 2-((2R,5S,6S) -6-((S,1E,3E) -6-((2R,3R) -3-((2R,3R,4R) -4-hydroxy-3-methoxypentan-2-yl) -2-methyloxiran-2-yl)- 5-methylhexa-1,3-dien-1-yl) -5-methyltetrahydro-2H-pyran-2-yl) acetate (S27): Prepared according to reported procedures. [9] To a mixture of alcohol **S26** (16 mg, 38 µmol, 1.0 eq) and $VO(acac)_3$ (2 mg, 8 µmol, 0.2 eq) in DCM (1.3 mL) at -78 °C was slowly added t-BuOOH (27 µL, 0.15 mmol, 4.0 eq, 5.5 M in decane). The resulting mixture was then stirred at -78 °C for 1 h and then kept at -20 °C for 24h, at which point the brown-red solution was partitioned between DCM (15 mL) and brine (5 mL). The aqueous layer was extracted with DCM (3 x 15 mL) and the combined organic layers were dried with Na_2SO_4, filtered, and concentrated under reduced pressure. Purification via flash chromatography (30% Et_2O/Hexanes) afforded the product **S27** (9.4 mg, 0.021 mmol) in 56% yield as a colorless oil.

$[\alpha]_D^{20}$ = +1.6 (c 3.1, $CHCl_3$); **^{1}H NMR** (500 MHz, $CDCl_3$): δ 6.15 (dd, *J* = 15.1, 10.5 Hz, 1H), 6.06 (dd, *J* = 15.0, 10.5 Hz, 1H), 5.55 – 5.45 (m, 2H), 3.82 – 3.75 (m, 2H), 3.66 (s, 3H), 3.56 – 3.54 (m, 1H), 3.53 (s, 3H), 3.42 (t, *J* = 8.3 Hz, 1H), 2.98 (dd, *J* = 6.4, 4.1 Hz, 1H), 2.66 (d, *J* = 9.5 Hz, 1H), 2.47 (dd, *J* = 7.6, 6.5 Hz, 2H), 2.44 – 2.38 (m, 1H), 1.92 (dd, *J* = 13.4, 4.4 Hz, 1H), 1.87 – 1.79 (m, 1H), 1.71 – 1.64 (m, 1H), 1.50 (dqd, *J* = 9.4, 7.0, 4.1 Hz, 1H), 1.36 – 1.31 (m, 2H), 1.27 (s, 3H), 1.23 – 1.16 (m, 1H), 1.11 (d, *J* = 6.5 Hz, 3H), 1.04 (d, *J* = 6.7 Hz, 3H), 0.92 – 0.88 (m, 1H), 0.85 (d, *J* = 7.0 Hz, 2H), 0.82 – 0.80 (m, 1H), 0.78 (d, *J* = 6.0 Hz, 3H); **^{13}C NMR** (125 MHz, $CDCl_3$): δ 173.5, 140.9, 134.1, 132.3, 130.4, 88.5, 86.2, 75.3, 69.9, 67.8, 62.6, 61.9, 52.1, 48.0, 42.0, 36.5, 36.4, 36.2, 33.3, 32.6, 22.5, 19.8, 18.1, 16.8, 11.5; **HRMS-ESI** (m/z): calcd. for $C_{25}H_{43}O_6$ [M + H]+: 439.3054, found 439.3051; **TLC**: R_f = 0.29 with Hex:EtOAc (1:1). p-anisaldehyde (dark spot).

C8-desmethyl-GEX1A (S28): A solution of **S27** (8.2 mg, 19 µmol, 1.0 eq) in MeOH (1.9 mL) and water (0.32 mL), was added K_2CO_3 (17 mg, 0.12 mmol, 6.5 eq) and refluxed for 2 h. The reaction was monitored by TLC, and after completion, it was cooled to rt and diluted with EtOAc (3 mL). The mixture was acidified to pH ~3 with HCl 1M, extracted with EtOAc (3 x 5 mL), the combined organic layers were dried with Na_2SO_4, filtered, and concentrated under reduced pressure. Purification via flash chromatography (DCM/ MeOH 3%) afforded the product **S28** (3.2 mg, 0.08 mmol) in 56% yield as a colorless oil.

$[\alpha]_D^{20}$ = +2.5 (c 0.2, MeOH); **^{1}H NMR** (500 MHz, MeOD-d_4): δ 6.16 (dd, *J* = 15.1, 10.4 Hz, 1H), 6.06 (dd, *J* = 15.0, 10.4 Hz, 1H), 5.48 – 5.55 (m, 2H), 3.84 – 3.74 (m, 2H), 3.53 (s, 3H), 3.43 (t, *J* = 8.3 Hz, 1H), 2.98 (dd, *J* = 6.3, 4.1 Hz, 1H), 2.66 (d, *J* = 9.4 Hz, 1H), 2.45 (m, 3H), 1.91 (dd, *J* = 13.4, 4.4 Hz, 1H), 1.87 – 1.82 (m, 1H), 1.72 (d, *J* = 11.7 Hz, 1H), 1.50 (ddd, *J* = 9.4, 6.9, 4.1 Hz, 1H), 1.27 (s, 3H), 1.21 (d, *J* = 11.2 Hz, 1H), 1.18 (d, *J* = 11.0 Hz, 1H), 1.11 (d, *J* = 6.4 Hz, 3H), 1.04 (d, *J* = 6.6 Hz, 3H), 0.93 – 0.86 (m, 1H), 0.85 (d, *J* = 7.0 Hz, 3H), 0.78 (d, *J* = 6.0 Hz, 3H); **^{13}C NMR** (125 MHz, MeOD-d_4): δ 140.9, 134.2, 132.3, 130.4, 88.5, 86.3, 75.6, 69.9, 67.8, 62.6, 61.9, 49.4, 48.0, 36.5, 36.4, 36.2, 33.4, 32.7, 22.4, 19.8, 18.1, 16.7, 11.5; **HRMS-ESI** (m/z): calcd. for $C_{24}H_{40}O_6$ [M + Na]+: 447.2717, found 447.2706; **TLC**: R_f = 0.54 with DCM:MeOH 3%. p-anisaldehyde (dark spot).

2-((2*R*,5*S*,6*S*)-6-((*S*,1*E*,3*E*)-6-((2*R*,3*R*)-3-((2*R*,3*R*,4*R*)-4-hydroxy-3-methoxypentan-2-yl)-2-methyloxiran-2-yl)-3,5-dimethylhexa-1,3-dien-1-yl)-5-methyltetrahydro-2*H*-pyran-2-yl)acetic acid (S30): Cross-coupling. $Pd(PPh_3)_4$ (12.0 mg, 10.0 µmol, 0.1 eq) was added to the solution of vinyl boronate **S13** (50 mg, 0.10 mmol, 1.0 eq), and vinyl iodide **S23** (44 mg, 0.13 mmol, 1.2 eq) in THF (1.9 mL)/H_2O (0.7 mL)/CH_3CN (0.3 mL) in a pressure vial, that was sealed and flushed with Ar. After stirring for 5 min, TlOEt (50 mg, 0.20 mmol, 2.0 eq) in THF (1.9 mL) was added and a grey precipitate appeared. The mixture was stirred at 30 °C until the reaction was completed (around 12h). The reaction was diluted with Et_2O (2.5 mL) and a sodium bisulfate sat solution (1.0 mL) to finalize it. The biphasic mixture was filtered through Celite, and the organic layer was separated, washed with brine, and dried over Na_2SO_4.

Purification via flash chromatography (2% Et_2O/Hexanes) afforded the triene product **S29** (51 mg, 0.092 mmol) in 92% yield as a colorless oil. R_f = 0.53 with Hex:EtOAc (4:1). p-anisaldehyde (blue spot).

Deprotection. A stirred ice-cooled solution of the triene **S29** (51 mg, 92 µmol) in anhydrous MeOH (1.0 mL, 0.1 M) was slowly treated with freshly prepared methanolic HCl (1.9 mL, 0.37 mmol, 4.0 eq, 0.2 M). The ice bath was removed, and the reaction was allowed to stir for 2 h. The resulting mixture was then quenched with solid $NaHCO_3$ until pH >7. The solution was filtered and washed with EtOAc and concentrated under reduced pressure. Purification via flash chromatography (30% Et_2O/Hexanes) afforded the product **S30** (29 mg, 66 µmol) in 72% yield as a colorless oil.

$[\alpha]_D^{20}$ = +1.74 (c 2.42, $CHCl_3$); **^{1}H NMR** (500 MHz, $CDCl_3$): δ 6.16 (d, *J* = 15.7 Hz, 1H), 5.43 (dd, *J* = 15.6, 7.7 Hz, 1H), 5.19 (d, *J* = 9.4, 1H), 4.92 (dd, *J* = 9.6, 1.3 Hz, 1H), 3.82 – 3.73 (m, 1H), 3.70 – 3.67 (m, 1H), 3.65 (s, 3H), 3.51 – 3.49 (m, 1H), 3.48 (s, 3H), 3.43 (dd, *J* = 9.3, 8.0 Hz, 1H), 2.70 – 2.63 (m, 2H), 2.63 – 2.57 (m, 2H), 2.40 (dd, *J* = 15.3, 6.7 Hz, 1H),2.18 (d, *J* = 7.0 Hz, 1H), 1.93 (d, *J* = 7.1 Hz, 2H), 1.82 (dd, *J* = 12.9, 3.5 Hz, 1H), 1.70 (d, *J* = 1.2 Hz, 3H), 1.68 – 1.66 (m, 1H), 1.59 (d, *J* = 1.4 Hz, 3H), 1.37 – 1.29 (m, 1H),1.27 – 1.20 (m, 1H), 1.17 (d, *J* = 6.5 Hz, 3H), 0.90 (d, *J* = 6.7 Hz, 3H), 0.88 (d, *J* = 6.6 Hz, 3H), 0.75 (d, *J* = 6.6 Hz, 3H); **^{13}C NMR** (125 MHz, $CDCl_3$): δ 171.9, 139.6, 138.1, 133.8, 131.6, 129.6, 126.0, 89.9, 85.6, 74.0, 68.0, 61.5, 51.7, 47.8, 41.4, 35.4, 34.8, 32.4, 31.7, 31.0, 20.7, 20.6, 18.0, 16.6, 16.5, 12.7; **HRMS-ESI** (m/z): calcd. for $C_{26}H_{44}O_5$ [M + H]+: 437.3261, found 437.3225; **TLC**: R_f = 0.29 with Hex:EtOAc (7:1). p-anisaldehyde (purple spot).

Methyl -2-((2*R*,5*S*,6*S*) -6-((*S*,1*E*,3*E*)-6-((2*R*,3*R*) -3-((2*R*,3*R*,4*R*) -4-hydroxy-3-methoxypentan-2-yl) -2-methyloxiran-2-yl) -3,5-dimethylhexa-1,3-dien-1-yl) -5-methyltetrahydro-2*H*-pyran-2-yl) acetate (S31): To a mixture of alcohol **S30** (16 mg, 38 µmol, 1.0 eq) and $VO(acac)_3$ (2 mg, 8 µmol, 0.2 eq) in DCM (1.3 mL) at -78 °C was slowly added t-BuOOH (27 µL, 0.15 mmol, 4.0 eq, 5.5 M in decane). The resulting mixture was then stirred at -78 °C for 1 h and then kept at -20 °C for 24h, at which point the brown-red solution was partitioned between DCM (15 mL) and brine (5 mL). The aqueous layer was extracted with DCM (3 x 15 mL) and the combined organic layers were dried with Na_2SO_4, filtered, and concentrated under reduced pressure. Purification via flash chromatography (30% Et_2O/Hexanes) afforded the product **S31** (21.7 mg, 0.048 mmol) in 72% yield as a colorless oil.

$[\alpha]_D^{20}$ = +4.2 (c 1.8, $CHCl_3$); **^{1}H NMR** (500 MHz, $CDCl_3$): δ 6.20 (d, *J* = 15.7 Hz, 1H), 5.46 (dd, *J* = 15.6, 7.8 Hz, 1H), 5.27 (m, *J* = 10.0 Hz, 1H), 3.83 – 3.77 (m, 1H), 3.78 (m, 1H), 3.66 (s, 3H), 3.56 – 3.54 (m, 1H), 3.53 (s, 3H), 3.45 (dd, *J* = 8.9, 7.5 Hz, 1H), 2.97 (dd, *J* = 6.3, 4.2 Hz, 1H), 2.82 – 2.74 (m, 1H), 2.67 (d, *J* = 9.4 Hz, 1H), 2.50 (dd, *J* = 15.4, 7.7 Hz, 1H), 2.45 (dd, *J* = 15.4, 5.5 Hz, 1H), 1.97 (dd, *J* = 13.3, 4.0 Hz, 1H), 1.89 – 1.83 (m, 1H), 1.78 (d, *J* = 1.2 Hz, 3H), 1.69 (m, 1H), 1.49 – 1.43 (m, 2H), 1.32 – 1.28 (m, 2H), 1.24 (s, 3H), 1.11 (d, *J* = 6.5 Hz, 3H), 1.00 (d, *J* = 6.7 Hz, 3H), 0.95 – 0.88 (m, 2H), 0.78 (d, *J* = 7.0 Hz, 3H), 0.78 (d, *J* = 6.4 Hz, 3H); **^{13}C NMR** (125 MHz, $CDCl_3$): δ 173.5, 139.6, 139.0, 133.1, 127.9, 88.5, 86.9, 75.3, 69.9, 67.9, 62.6, 61.9, 52.1, 48.2, 42.0, 37.6, 36.6, 36.3, 33.4, 31.4, 22.2, 19.8, 18.2, 16.8, 12.8, 11.3; **HRMS-ESI** (m/z): calcd. for $C_{26}H_{44}O_6$ [M + H]+: 453.3211, found 453.3202; **TLC**: R_f = 0.32 with Hex:EtOAc (1:1). p-anisaldehyde (black spot).

C10-methyl-shifted-GEX1A (S32): A solution of **S31** (9.0 mg, 20 µmol, 1.0 eq) in MeOH (2.0 mL) and water (0.33 mL), was added K_2CO_3 (18 mg, 0.13 mmol, 6.5 eq) and refluxed for 2 h. The reaction was monitored by TLC, and after completion, it was cooled to rt and diluted with EtOAc (3 mL). The mixture was acidified to pH ~3 with HCl 1M, extracted with EtOAc (3 x 5 mL), the combined organic layers were dried with Na_2SO_4, filtered, and concentrated under reduced pressure. Purification via flash chromatography (DCM/ MeOH 3%) afforded the product **S32** (6.0 mg, 14 µmol) in 68% yield as a colorless oil.

$[\alpha]_D^{20}$ = +25.8 (c 0.33, MeOH); **^{1}H NMR** (500 MHz, MeOD-d_4): δ 6.21 (d, *J* = 15.7 Hz, 1H), 5.46 (dd, *J* = 15.6, 7.9 Hz, 1H), 5.27 (d, *J* = 9.9 Hz, 1H), 3.83 – 3.75 (m, 2H), 3.53 (s, 3H), 3.46 (t, *J* = 8.6 Hz, 1H), 2.98 (dd, *J* = 6.3, 4.2 Hz, 1H), 2.78 (m, 1H), 2.67 (d, *J* = 9.5 Hz, 1H), 2.52 – 2.44 (m, 1H), 2.44 – 2.36 (m, 1H), 1.97 (dd, *J* = 13.3, 4.0 Hz, 1H), 1.89 – 1.83 (m, 1H), 1.78 (s, 3H), 1.73 (m, 1H),1.71 (m, 1H), 1.47 (ddd, *J* = 9.8, 6.9, 4.1 Hz, 1H), 1.32 – 1.28 (m, 2H), 1.24 (s, 3H), 1.17 (dd, *J* = 13.4, 11.1 Hz, 1H), 1.11 (d, *J* = 6.4 Hz, 3H), 1.00 (d, *J* = 6.7 Hz, 3H), 0.93 – 0.84 (m, 2H), 0.78 (d, *J* = 5.3 Hz, 3H), 0.77 (d, *J* = 4.8 Hz, 3H); **^{13}C NMR** (125 MHz, MeOD-d_4): δ 139.6, 139.1, 133.1, 127.9, 88.5, 87.0, 75.6, 69.9, 67.9, 62.6, 61.9, 49.3, 48.2, 36.6, 36.3, 33.4, 32.7, 31.4, 22.2, 19.8, 18.3, 16.8, 12.8, 11.3; **HRMS-ESI** (m/z): calcd. for $C_{25}H_{42}O_6$ [M + Na]+: 461.2874, found 461.2867; **TLC**: R_f = 0.29 with DCM:MeOH (9:1). p-anisaldehyde (dark spot).

methyl 2-((2*R*,5*S*,6*S*)-6-((2*E*,4*E*,6*S*,8*E*,10*S*,11*R*,12*R*)-12-hydroxy-11-methoxy-4,6,8,10-tetramethyltrideca-2,4,8-trien-2-yl)-5-methyltetrahydro-2*H*-pyran-2-yl)acetate (S34): Cross-coupling. $Pd(PPh_3)_4$ (12.0 mg, 10.0 µmol, 0.1 eq) was added to the solution of vinyl boronate **S12** (49 mg, 0.10 mmol, 1.0 eq), and vinyl iodide **S23** (41 mg, 0.12 mmol, 1.2 eq) in THF (1.7 mL)/H_2O (0.6 mL)/CH_3CN (0.3 mL) in a pressure vial, that was sealed and flushed with Ar. After stirring for 5 min, TlOEt (50 mg, 0.20 mmol, 2.0 eq) in THF (1.9 mL) was added and a grey precipitate appeared. The mixture was stirred at 30 °C until the reaction was completed (around 12h). The reaction was diluted with Et_2O (2.5 mL) and a sodium bisulfate sat solution (1.0 mL) to finalize it. The biphasic mixture was filtered through Celite, and the organic layer was separated, washed with brine, and dried over Na_2SO_4. Purification via flash chromatography (2% Et_2O/Hexanes) afforded the triene product **S33** (32 mg, 0.06 mmol) in 60% yield as a colorless oil. R_f = 0.43 with Hex:Et_2O (4:1). p-anisaldehyde (blue spot).

Deprotection. A stirred ice-cooled solution of the triene **S33** (41 mg, 72 µmol) in anhydrous MeOH (3.6 mL, 0.1 M) was slowly treated with freshly prepared methanolic HCl (1.5 mL, 0.3 mmol, 4.0 eq, 0.2 M). The ice bath was removed, and the reaction was allowed to stir for 2 h. The resulting mixture was then quenched with solid $NaHCO_3$ until pH >7. The solution was filtered and washed with EtOAc and concentrated under reduced pressure. Purification via flash chromatography (30% Et_2O/Hexanes) afforded the product **S34** (14.7 mg, 26 µmol) in 37% yield as a colorless oil.

[α]D20 = +45.3 (c 1.2, $CHCl_3$); **1H NMR** (500 MHz, $CDCl_3$): δ 5.74 (s, 1H), 5.07 (d, *J* = 9.3 Hz, 1H), 4.96 (dd, *J* = 9.6, 1.3 Hz, 1H), 3.80 – 3.73 (m, 1H), 3.70 (dd, *J* = 6.5, 4.3 Hz, 1H), 3.66 (s, 3H), 3.50 (s, 3H), 3.49 – 3.45 (m, 1H), 3.29 (d, *J* = 9.8 Hz, 1H), 2.70 (dd, *J* = 6.7, 4.2 Hz, 1H), 2.67 – 2.56 (m, 2H), 2.40 (dd, *J* = 15.1, 7.0 Hz, 1H), 1.95 (ddd, *J* = 7.1, 5.9, 1.1 Hz, 1H), 1.83 (dq, *J* = 13.2, 3.4 Hz, 1H), 1.71 (m, 3H), 1.71 (m, 3H), 1.70 (dd, *J* = 4.5, 1.5 Hz, 1H), 1.68 – 1.65 (m, 1H), 1.61 (d, *J* = 1.3 Hz, 3H), 1.56 – 1.49 (m, 1H), 1.35 – 1.31 (m, 1H), 1.29 (dd, *J* = 5.8, 3.2 Hz, 1H), 1.19 (d, *J* = 6.4 Hz, 3H), 0.94 (d, *J* = 6.7 Hz, 3H),0.89 (d, *J* = 6.6 Hz, 3H), 0.71 (d, *J* = 6.6 Hz, 3H); **13C NMR** (125 MHz, $CDCl_3$): δ 172.0, 136.8, 134.1, 133.8, 132.5, 130.6, 129.5, 91.38, 90.0, 73.9, 68.0, 61.6, 51.7, 48.0, 41.5, 34.8, 32.5, 32.4, 31.8, 30.9, 20.8, 20.6, 18.0, 17.1, 16.5, 16.4, 13.4; **HRMS-ESI** (m/z): calcd. for $C_{27}H_{46}O_5$ [M + H]+: 451.3418, found 451.3408; **TLC**: R_f = 0.28 with Hex:EtOAc (7:3). p-anisaldehyde (dark spot).

Methyl -2-((2*R*,5*S*,6*S*) -6-((*S*,1*E*,3*E*)-6-((2*R*,3*R*) -3-((2*R*,3*R*,4*R*) -4-hydroxy-3-methoxypentan-2-yl) -2-methyloxiran-2-yl) -3,5-dimethylhexa-1,3-dien-1-yl) -5-methyltetrahydro-2*H*-pyran-2-yl) acetate (S35): To a mixture of alcohol **S34** (16 mg, 38 μmol, 1.0 eq) and $VO(acac)_3$ (2 mg, 8 μmol, 0.2 eq) in DCM (1.3 mL) at -78 °C was slowly added t-BuOOH (27 μL, 0.15 mmol, 4.0 eq, 5.5 M in decane). The resulting mixture was then stirred at -78 °C for 1 h and then kept at -20 °C for 24h, at which point the brown-red solution was partitioned between DCM (15 mL) and brine (5 mL). The aqueous layer was extracted with DCM (3 x 15 mL) and the combined organic layers were dried with Na_2SO_4, filtered, and concentrated under reduced pressure. Purification via flash chromatography (30% Et_2O/Hexanes) afforded the product **S35** (21.7 mg, 0.048 mmol) in 72% yield as a colorless oil.
[α]D20 = +4.2 (c 1.8, $CHCl_3$); **1H NMR** (500 MHz, $CDCl_3$): δ 6.20 (d, *J* = 15.7 Hz, 1H), 5.46 (dd, *J* = 15.6, 7.8 Hz, 1H), 5.27 (m, *J* = 10.0 Hz, 1H), 3.83 – 3.77 (m, 1H), 3.78 (m, 1H), 3.66 (s, 3H), 3.56 – 3.54 (m, 1H), 3.53 (s, 3H), 3.45 (dd, *J* = 8.9, 7.5 Hz, 1H), 2.97 (dd, *J* = 6.3, 4.2 Hz, 1H), 2.82 – 2.74 (m, 1H), 2.67 (d, *J* = 9.4 Hz, 1H), 2.50 (dd, *J* = 15.4, 7.7 Hz, 1H), 2.45 (dd, *J* = 15.4, 5.5 Hz, 1H), 1.97 (dd, *J* = 13.3, 4.0 Hz, 1H), 1.89 – 1.83 (m, 1H), 1.78 (d, *J* = 1.2 Hz, 3H), 1.69 (m, 1H), 1.49 – 1.43 (m, 2H), 1.32 – 1.28 (m, 2H), 1.24 (s, 3H), 1.11 (d, *J* = 6.5 Hz, 3H), 1.00 (d, *J* = 6.7 Hz, 3H), 0.95 – 0.88 (m, 2H), 0.78 (d, *J* = 7.0 Hz, 3H), 0.78 (d, *J* = 6.4 Hz, 3H); **13C NMR** (125 MHz, $CDCl_3$): δ 173.5, 139.6, 139.0, 133.1, 127.9, 88.5, 86.9, 75.3, 69.9, 67.9, 62.6, 61.9, 52.1, 48.2, 42.0, 37.6, 36.6, 36.3, 33.4, 31.4, 22.2, 19.8, 18.2, 16.8, 12.8, 11.3; **HRMS-ESI** (m/z): calcd. for $C_{26}H_{44}O_6$ [M + H]+: 453.3211, found 453.3202; **TLC**: R_f = 0.32 with Hex:EtOAc (1:1). p-anisaldehyde (black spot).

C8,C10-dimethyl-GEX1A (S36): A solution of **S35** (20.0 mg, 43 μmol, 1.0 eq) in THF (0.2 mL), was added TMSOK (58 mg, 0.45 mmol, 10 eq) and stirred for 12 h. The reaction was monitored by TLC. After completion, the mixture was acidified to pH ~3 with citric acid 1M and extracted with EtOAc (3 x 5 mL). The combined organic layers were dried with Na_2SO_4, filtered, and concentrated under reduced pressure. Purification via flash chromatography DCM:MeOH (9:1) afforded the product **S36** (19.4 mg, 42 μmol) in 98% yield as a colorless oil.
[α]D20 = +11.7 (c 0.33, MeOH); **1H NMR** (500 MHz, MeOD-d_4): δ 5.81 (s, 1H), 5.15 – 5.08 (m, 1H), 3.84 – 3.73 (m, 3H), 3.54 (m, 3H), 3.34 (s, 1H), 3.00 (dd, *J* = 6.3, 4.2 Hz, 1H), 2.76 – 2.71 (m, 1H), 2.68 (d, *J* = 9.4 Hz, 1H), 2.49 (dd, *J* = 15.3, 6.9 Hz, 1H), 2.40 (dd, *J* = 15.4, 5.4 Hz, 1H), 1.88 (d, *J* = 4.7 Hz, 1H), 1.86 (d, *J* = 4.3 Hz, 1H), 1.81 (d, *J* = 1.4 Hz, 3H), 1.77 (d, *J* = 1.4 Hz, 1H), 1.75 (d, *J* = 1.4 Hz, 3H), 1.73 – 1.69 (m, 1H), 1.57 (ddq, *J* = 13.7, 6.1, 3.0 Hz, 1H), 1.50 (ddd, *J* = 9.5, 6.9, 4.2 Hz, 1H), 1.37 – 1.32 (m, 2H), 1.27 (s, 3H), 1.12 (d, *J* = 6.6 Hz, 3H), 1.02 (d, *J* = 6.7 Hz, 3H), 0.86 (d, *J* = 7.0 Hz, 3H), 0.73 (d, *J* = 6.6 Hz, 3H); **13C NMR** (125 MHz, MeOD-d_4): δ 137.7, 135.2, 133.6, 132.3, 92.9, 88.5, 75.5, 69.9, 67.7, 62.7, 61.9, 48.1, 36.3, 33.5, 33.3, 32.8, 31.3, 22.4, 19.8, 18.2, 17.2, 16.9, 13.7, 11.1; **HRMS-ESI** (m/z): calcd. for $C_{26}H_{44}O_6$ [M + Na]+: 475.3030, found 475.3027; **TLC**: R_f = 0.29 with DCM:MeOH (9:1). p-anisaldehyde (dark spot).

Growth Inhibition Assay

Cell culture and EC50 measurement
Molm-13 acute myeloid leukemia cells were maintained in RPMI 1640 medium supplemented with 10% fetal bovine serum (FBS), and HeLa cells were maintained in DMEM supplemented with 10% FBS. Cytotoxicity of GEX1A analogs was determined using the Cell Counting Kit-8 assay (APExBio, #K1018). Briefly, 100 μL of Molm-13 cell suspension (1×10^5 cells/mL) or HeLa cell suspension (5×10^4 cells/mL) was seeded into each well of a 96-well plate. Cells remained in culture for 24 hours at 37 °C in a 5% CO_2 incubator prior to treatment. Test compounds were serially diluted in culture medium containing DMSO, and 10 μL of each compound dilution was added to each well, ensuring a final DMSO concentration of ≤1%. Cells were treated for 48 hours, then 10 μL of CCK-8 reagent was added to each well. Absorbance at 450 nm (reference 650 nm) was measured after 4 hours of incubation for Molm-13 cells or after 1.5 hours for HeLa cells. Cell viability was calculated by dividing the background-corrected absorbance of the treated group by that of the DMSO control. EC50 values were determined using GraphPad Prism. All assays were performed in triplicate.

HeLa	Log IC_{50}	IC_{50}
GEX1A (**1**)	-7.874	13.4 nM
C8-Desmethyl (**2**)	-5.16	6.94 μM
C10-Methyl shifted (**3**)	Unstable	Unstable
C8,C10-Dimethyl (**4**)	-1.75	17.9 mM

Molm-13	Log IC_{50}	IC_{50}
GEX1A (**1**)	-8.40	4.00 nM
C8-Desmethyl (**2**)	-5.38	4.17 μM
C10-Methyl shifted (**3**)	-3.34	461 μM
C8,C10-Dimethyl (**4**)	-3.97	107 μM

References

[1] M. Banwell, M. McLeod, R. Premraj, G. Simpson. *Pure Appl. Chem.* **2000**, *72*, 1631-1634.
[2] M. Banwell, C. Bui, G. Simpson. *Chem. Soc. Perkin Trans*. **1998**, *1*, 791–800.
[3] A. Chatterjee, T. Choi, D. Sanders, R. Grubbs. *J. Am. Chem. Soc.* **2003,** *125*,11360–11370.
[4] H. Blackwell, D. O'Leary, A. Chatterjee, R. Washenfelder, A. Bussmann, R. Grubbs H. *J. Am. Chem. Soc*. **2000**, *122*, 58–71.
[5] C. Schneider, A. Schuffenhauer. *European J. Org. Chem*. **2000**, 73–82.
[6] M. Pellicena,, K. Krämer, P. Romea, F. Urpí. *Org. Lett.* **2011**, *13*, 5350–5353.
[7] A. Ghosh, J. Li. *Org. Lett.* **2011**, *13*, 66–69.
[8] S. A. Frank, H. Chen, R. K. Kunz, M. J. Schnaderbeck, W. R. Roush. *Org. Lett*. **2000**, *2*, 2691-2694.
[9] P. R. Blakemore, P. J. Kocieński, A. Morley, K. Muir. *J. Chem. Soc., Perkin Trans. 1*. **1999**, 955-968.

^{1}H and ^{13}C spectra

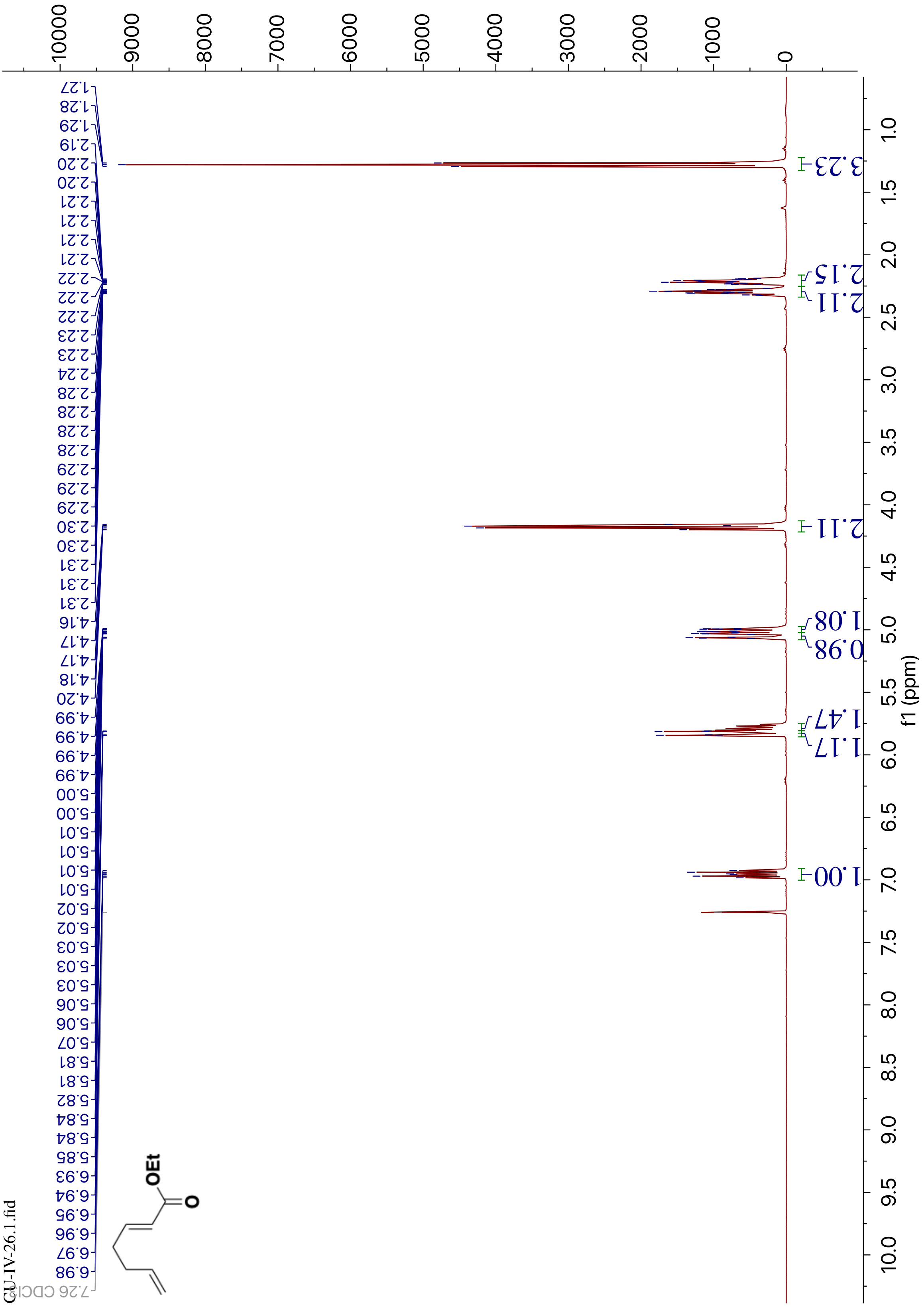

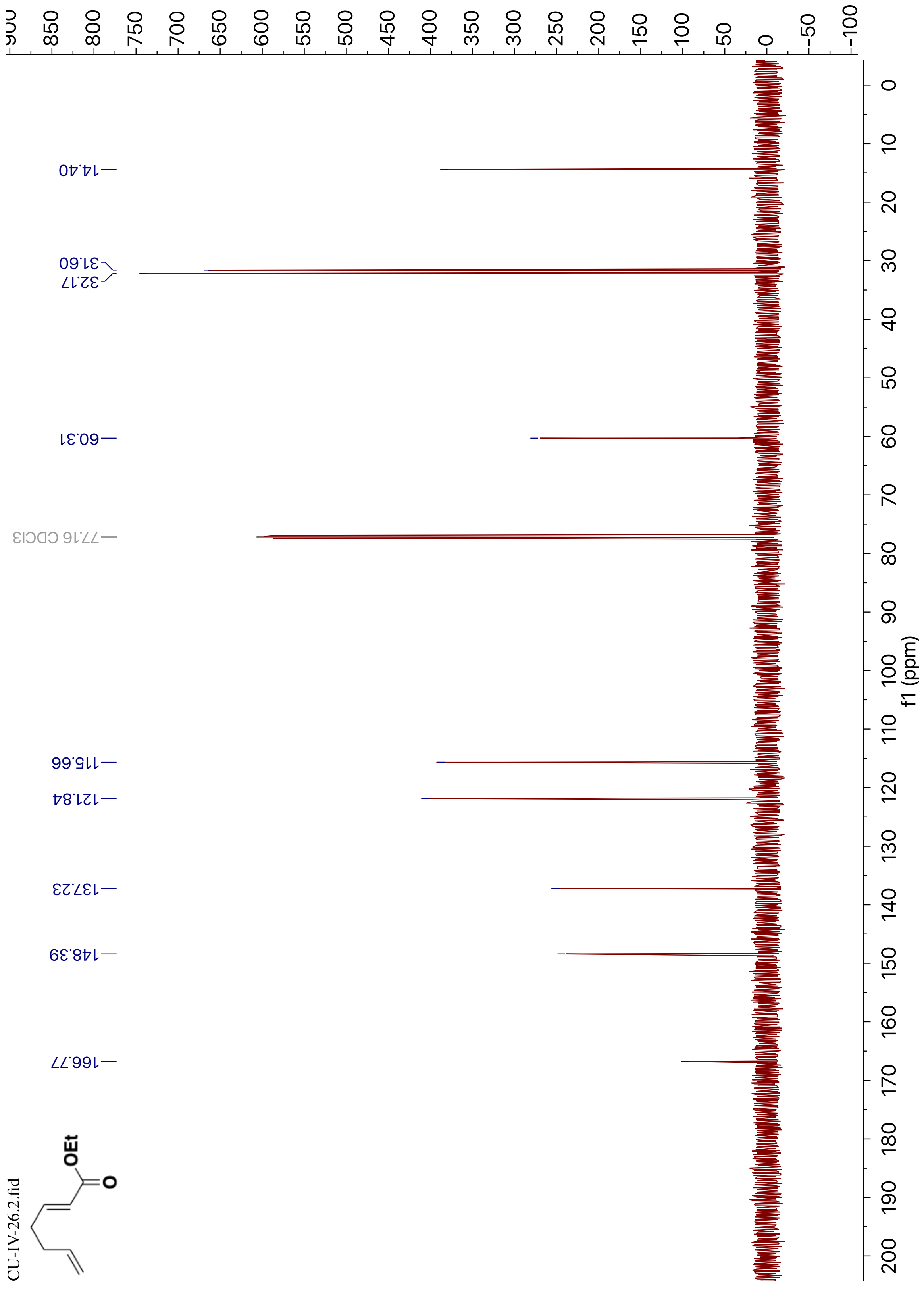
CU-IV-26.2.fid
OEt
O
14.40
31.60
32.17
60.31
77.16 CDCl3
115.66
121.84
137.23
148.39
166.77
f1 (ppm)

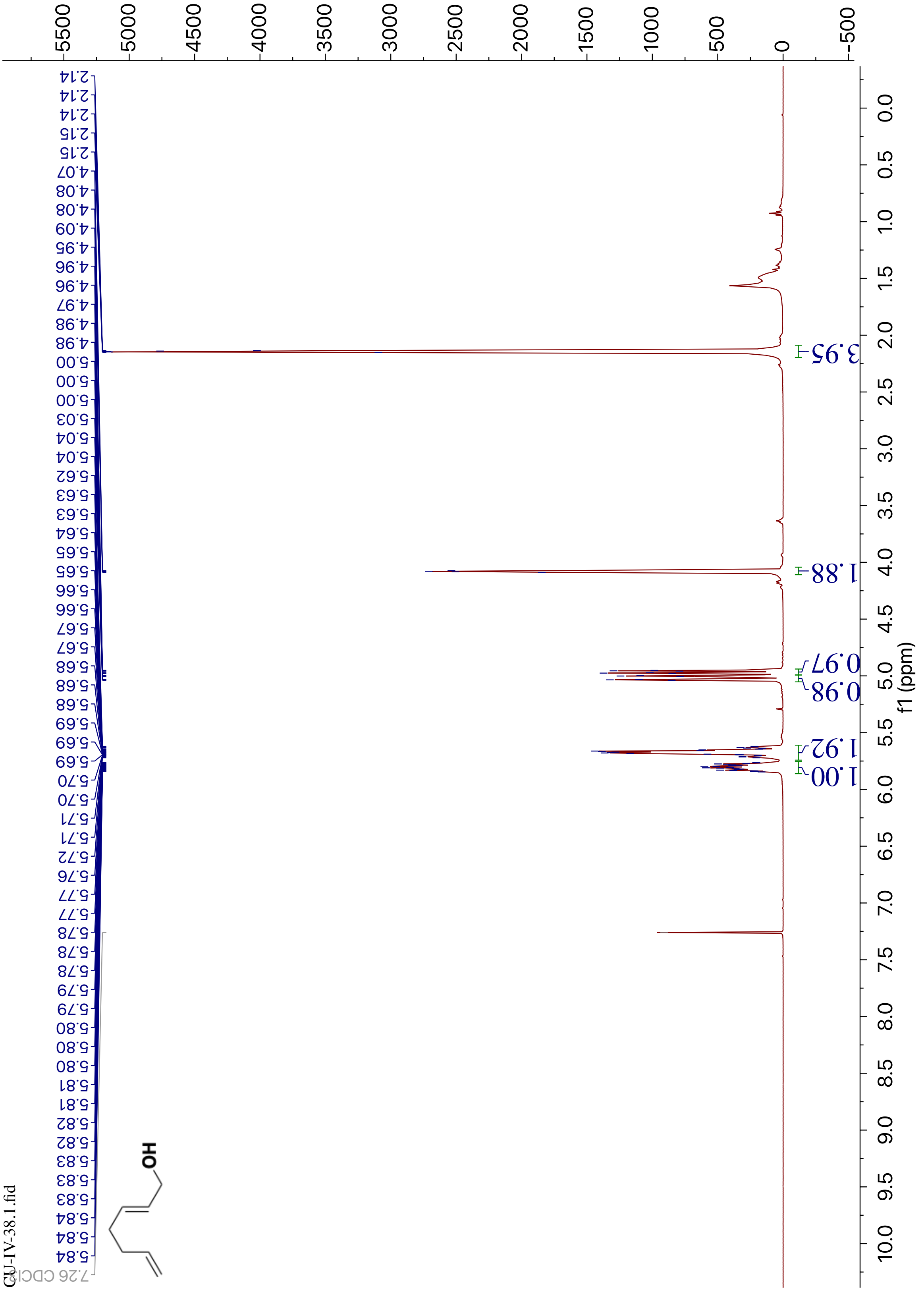
CU-IV-38.1.fid
7.26 CDCl3
OH
f1 (ppm)
3.95
1.88
0.97
0.98
1.92
1.00

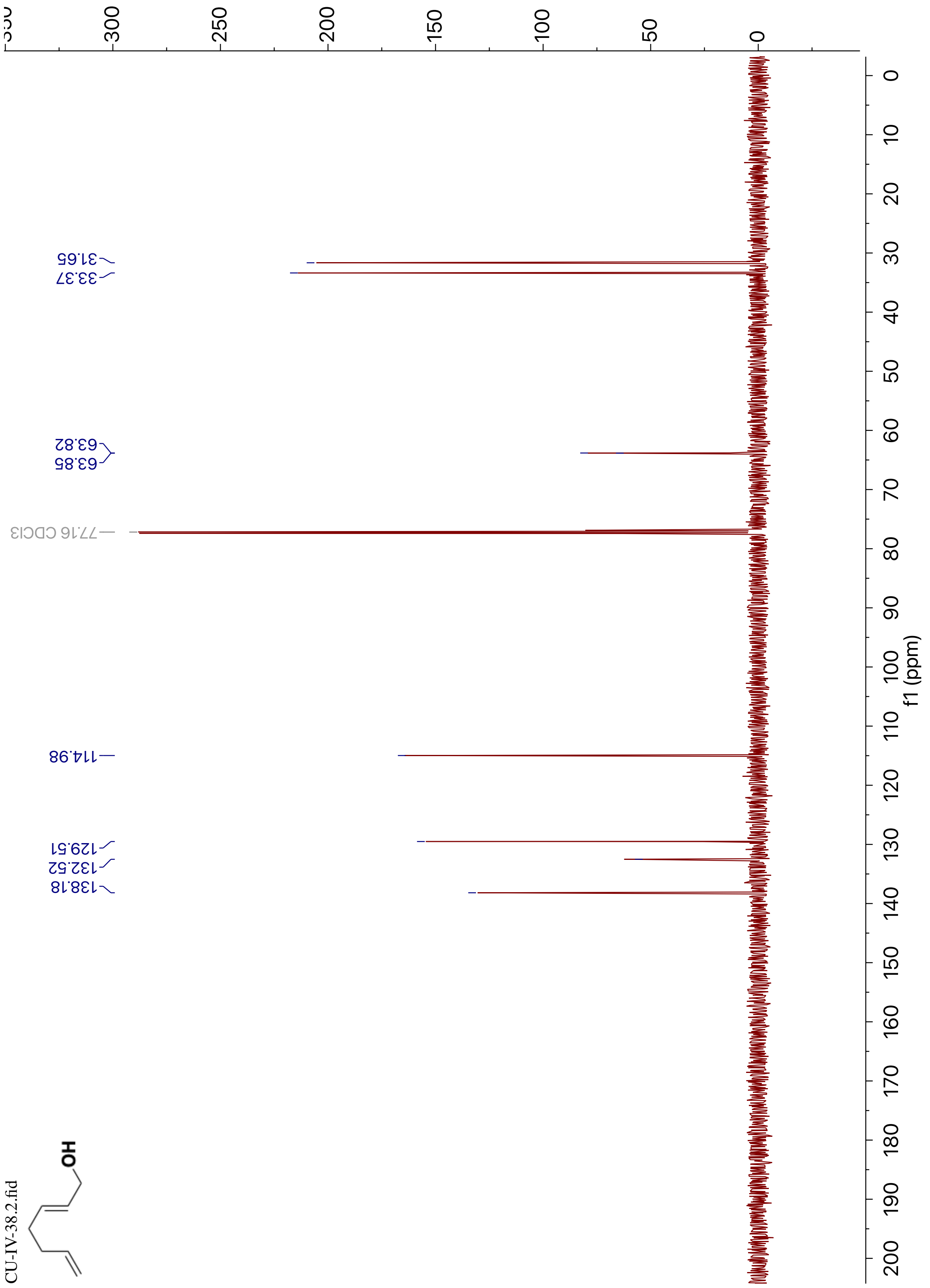
CU-IV-38.2.fid
OH
31.65
33.37
63.82
63.85
77.16 CDCl3
114.98
129.51
132.52
138.18
f1 (ppm)

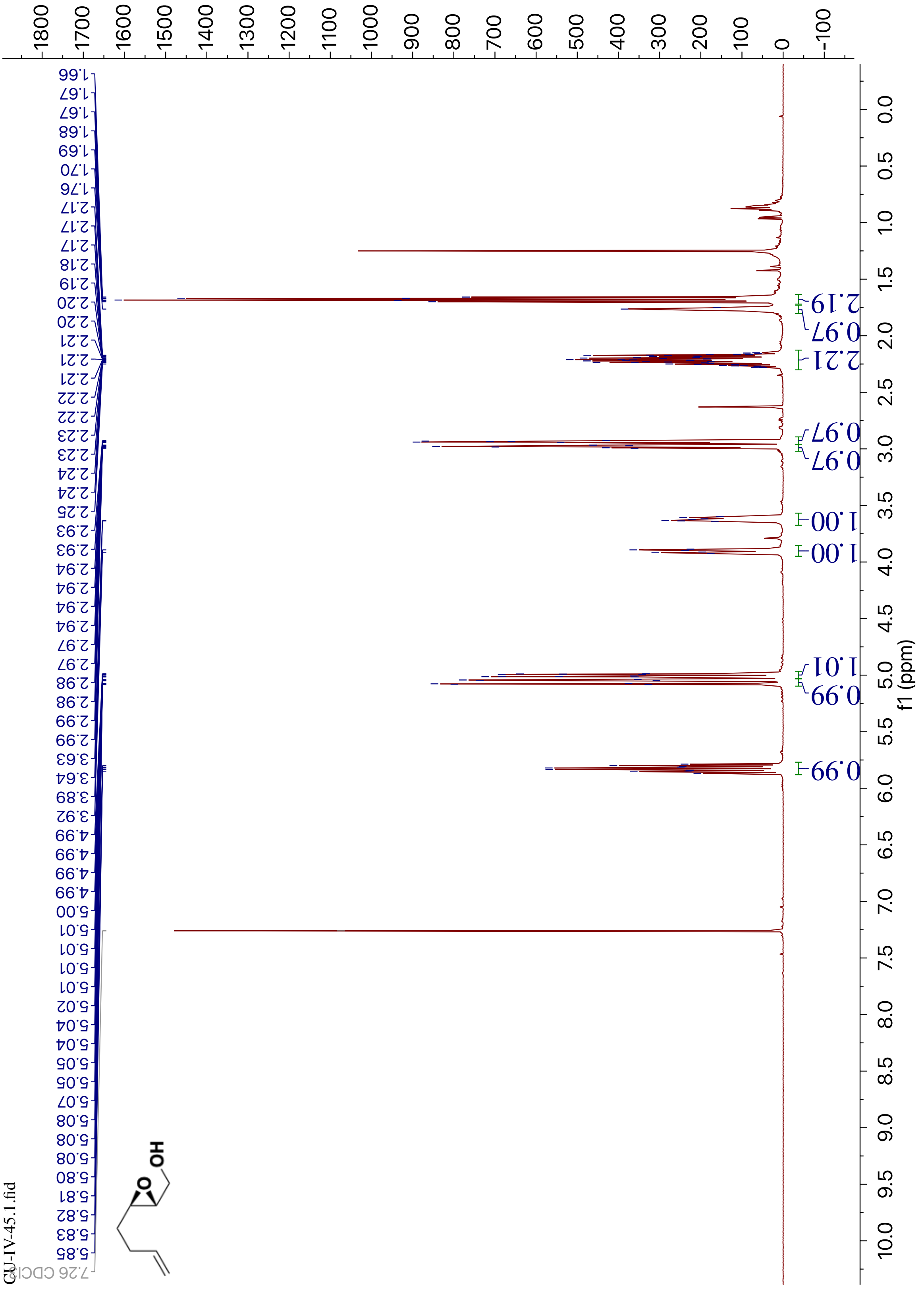
GU-IV-45.1.fid
7.26 CDCl3
OH
O
f1 (ppm)

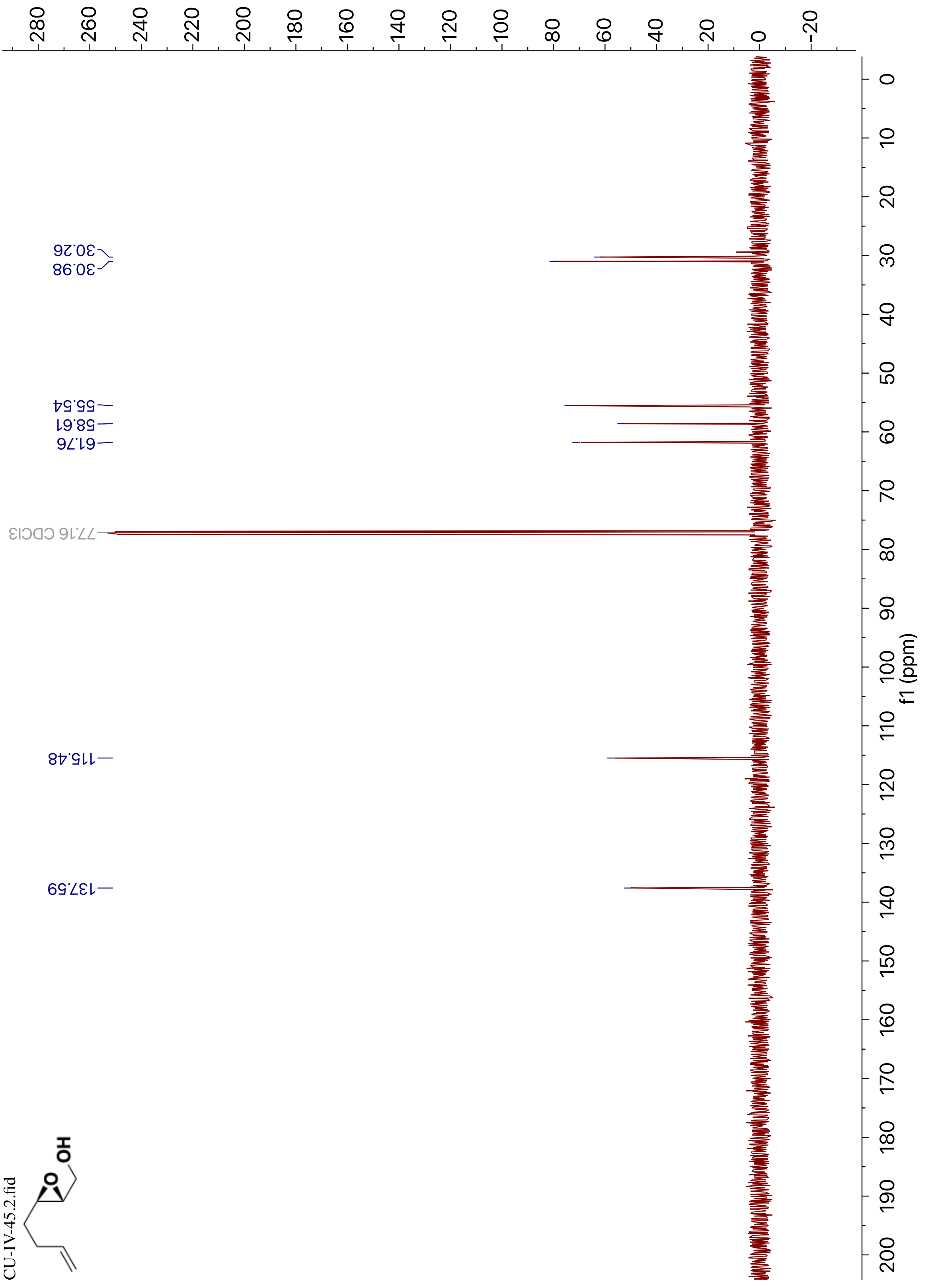
CU-IV-45.2.fid
OH
O
30.26
30.98
55.54
58.61
61.76
77.16 CDCl3
115.48
137.59
f1 (ppm)

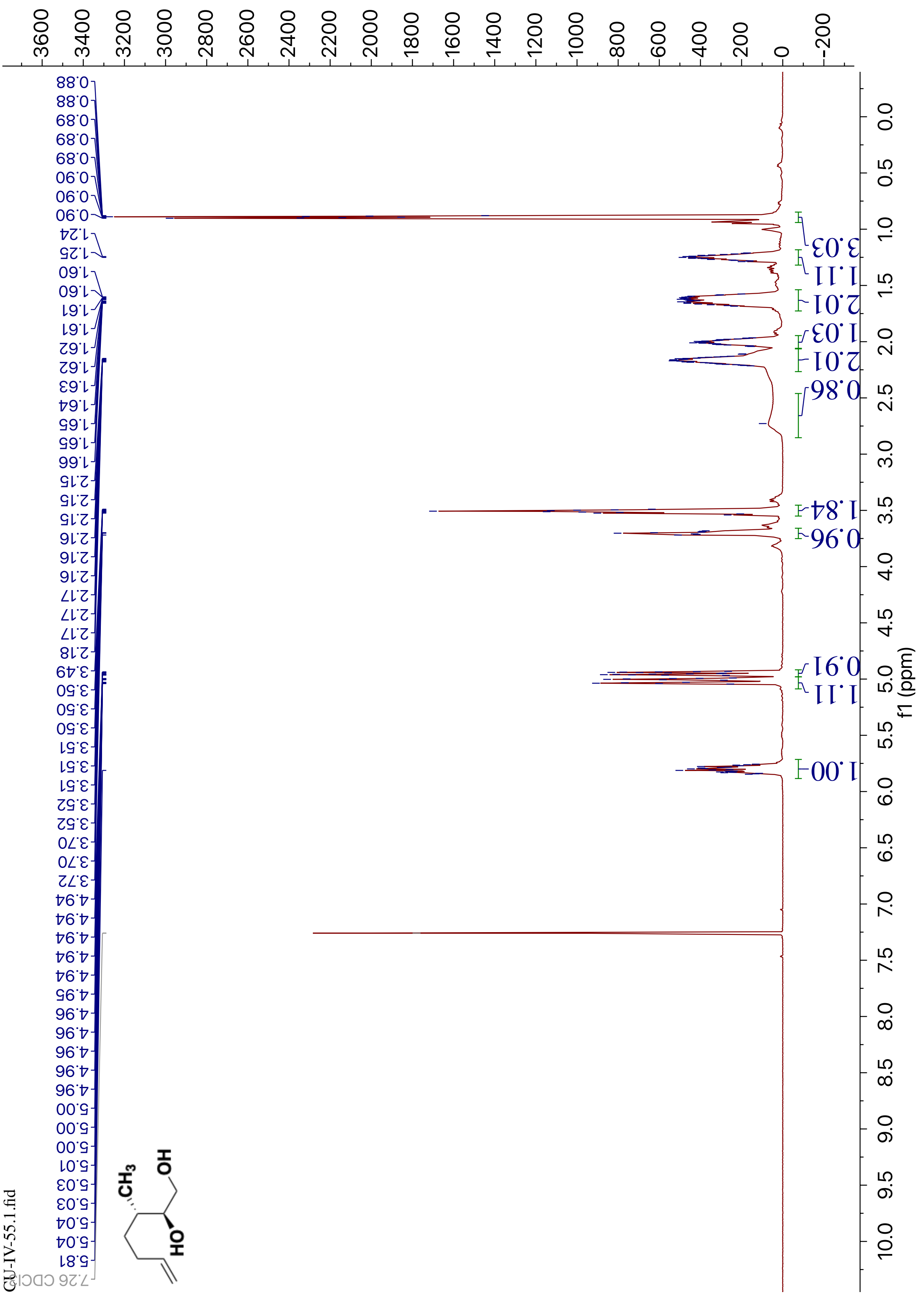

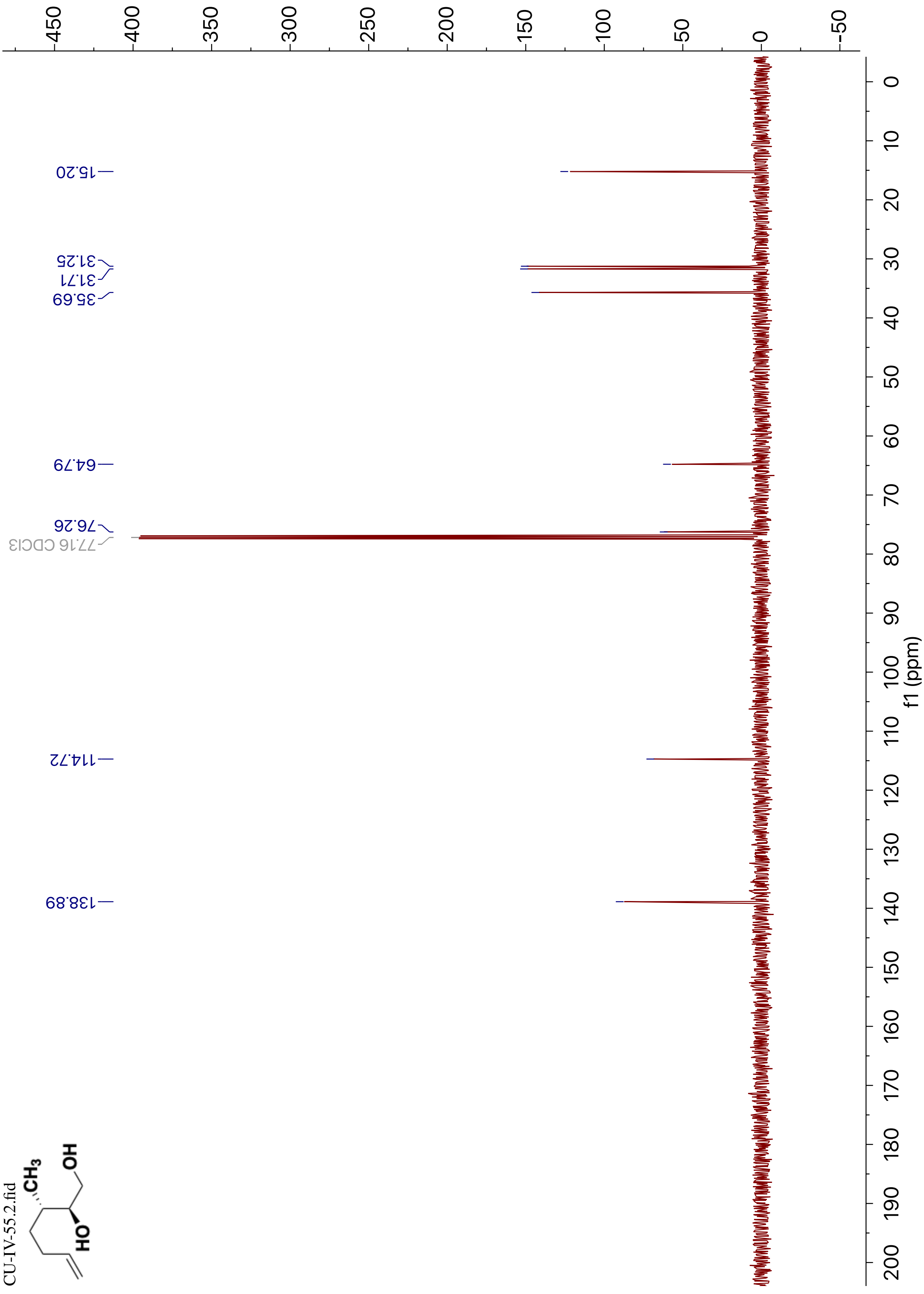
CU-IV-55.2.fid
CH3
OH
HO
15.20
31.25
31.71
35.69
64.79
76.26
77.16 CDCl3
114.72
138.89
f1 (ppm)

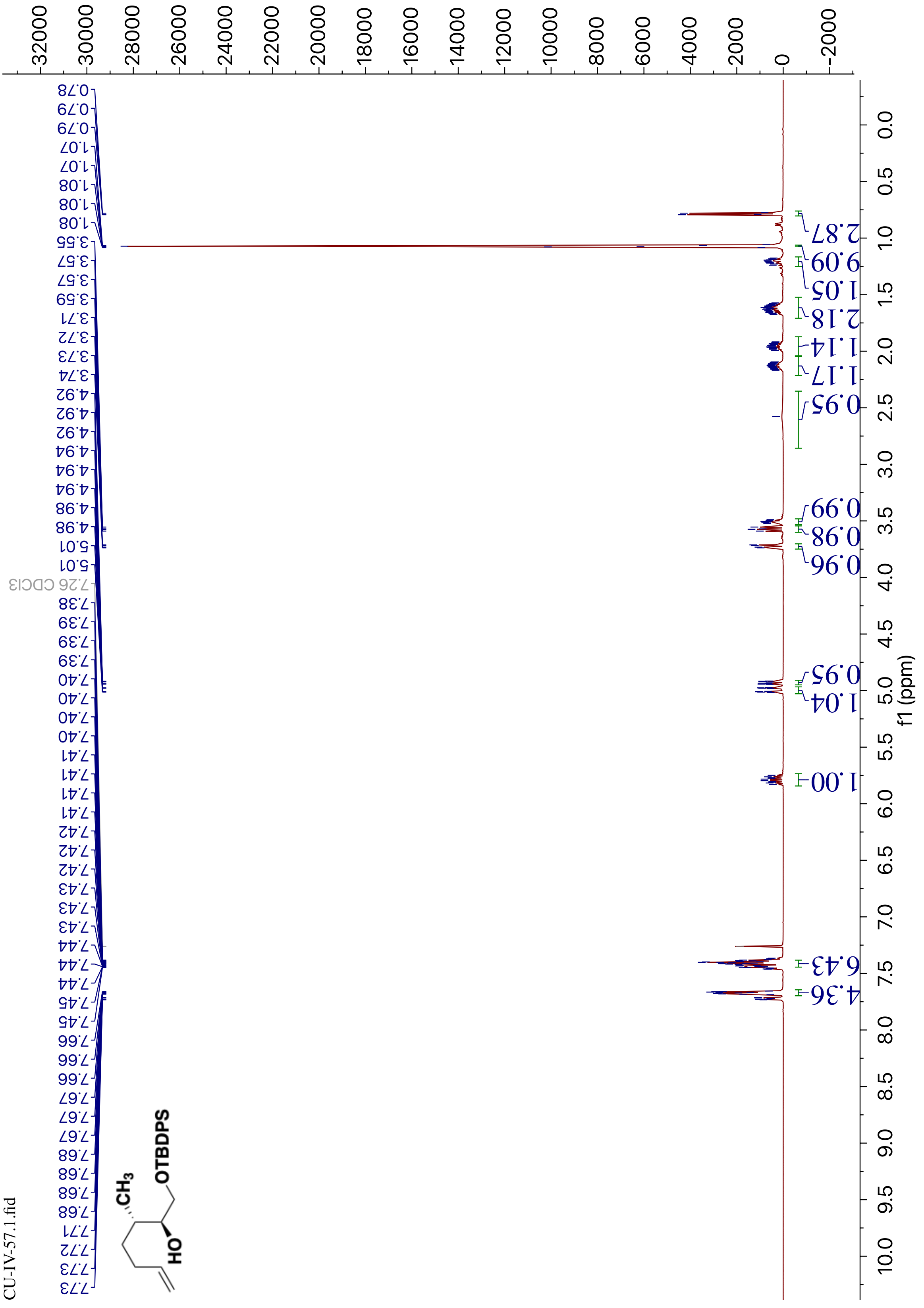
CU-IV-57.1.fid
0.78
0.79
0.79
1.07
1.07
1.08
1.08
1.08
3.55
3.57
3.57
3.59
3.71
3.72
3.73
3.74
4.92
4.92
4.92
4.94
4.94
4.94
4.98
4.98
5.01
5.01
7.26 CDCl3
7.38
7.39
7.39
7.39
7.40
7.40
7.40
7.40
7.41
7.41
7.41
7.41
7.42
7.42
7.42
7.43
7.43
7.43
7.44
7.44
7.44
7.45
7.45
7.66
7.66
7.66
7.67
7.67
7.67
7.68
7.68
7.68
7.68
7.71
7.72
7.73
7.73
CH3
OTBDPS
HO
2.87
9.09
1.05
2.18
1.14
1.17
0.95
0.99
0.98
0.96
0.95
1.04
1.00
6.43
4.36
f1 (ppm)

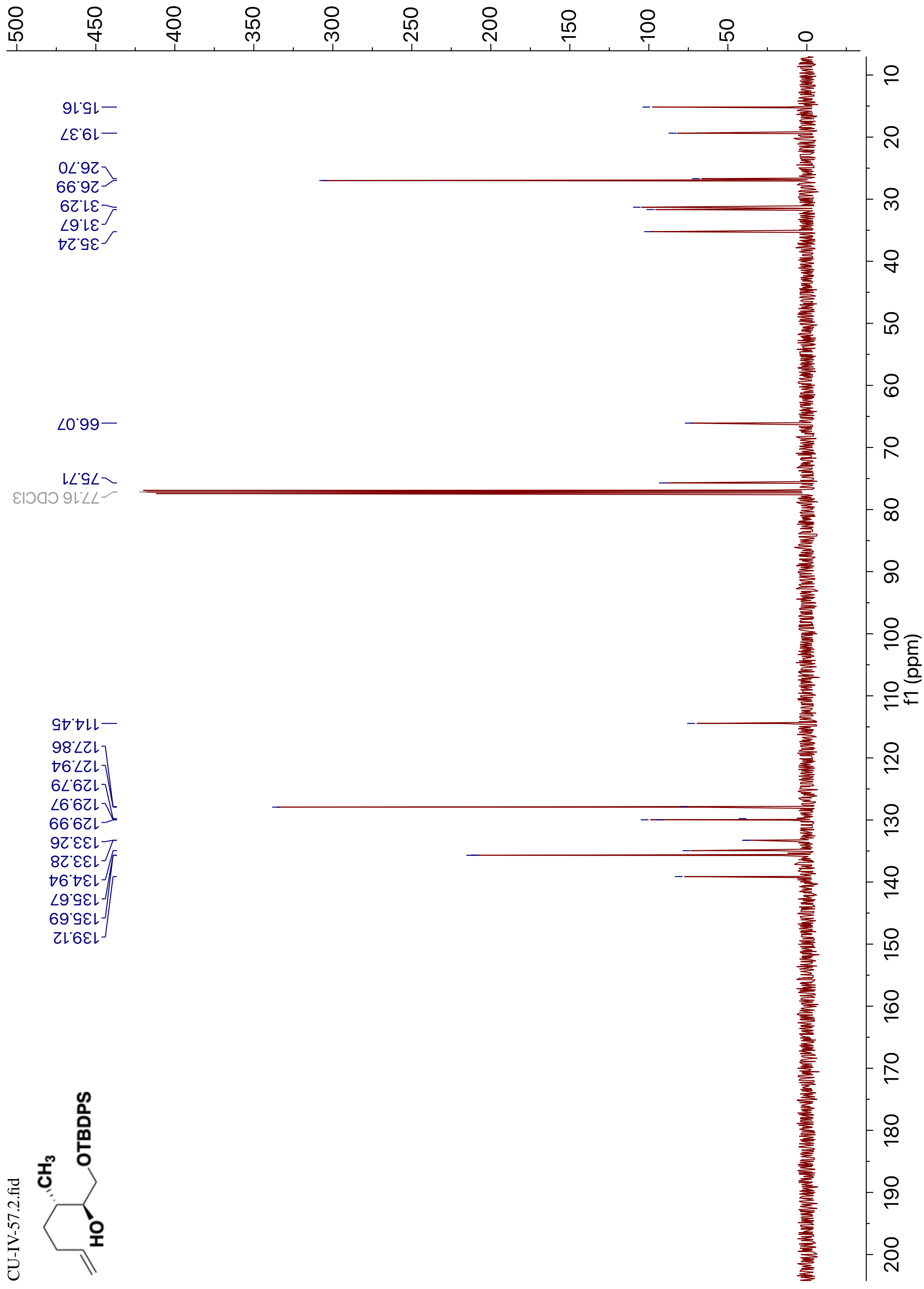
CU-IV-57.2.fid
HO
CH3
OTBDPS
15.16
19.37
26.70
26.99
31.29
31.67
35.24
66.07
75.71
77.16 CDCl3
114.45
127.86
127.94
129.79
129.97
129.99
133.26
133.28
134.94
135.67
135.69
139.12
f1 (ppm)

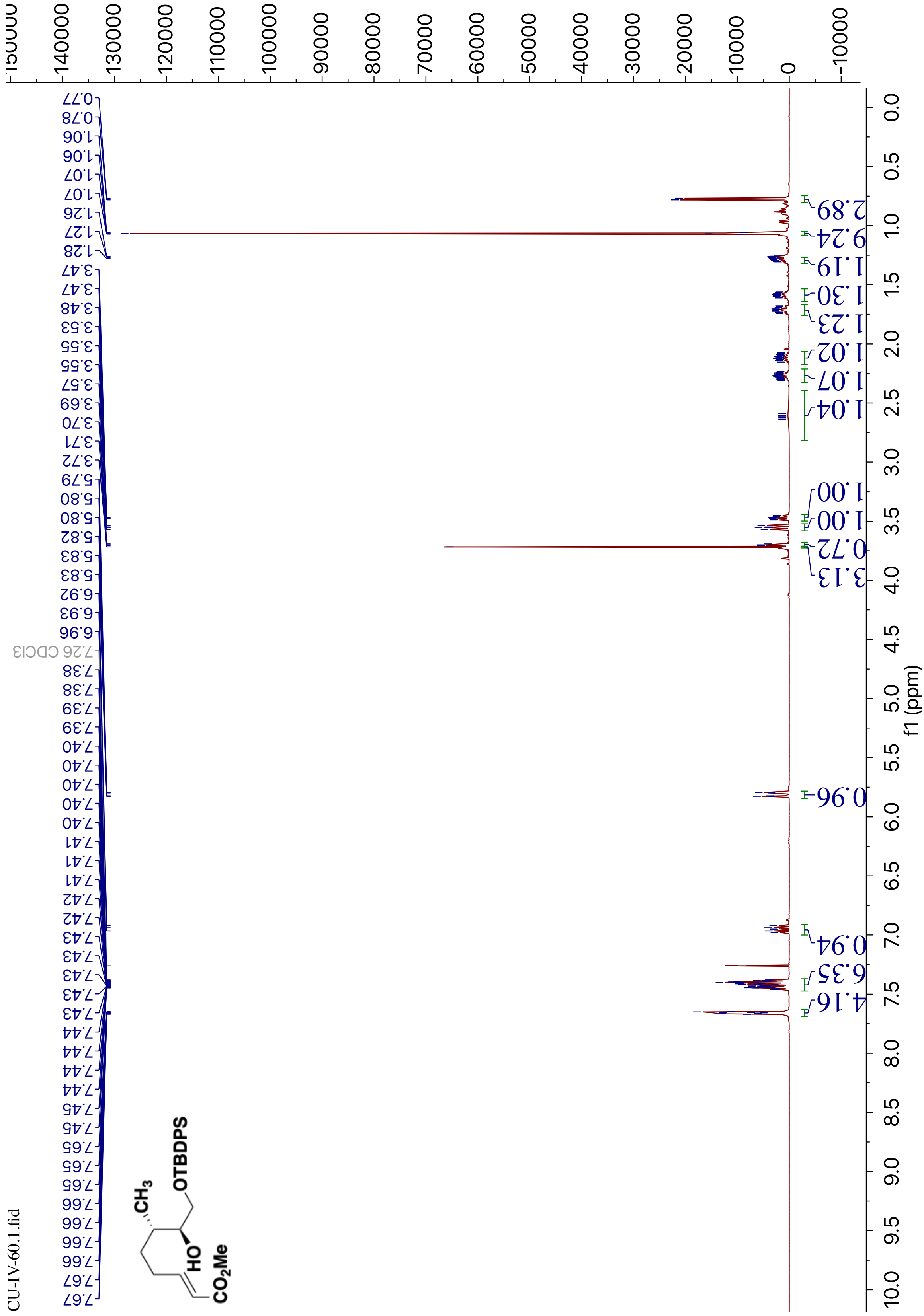
CU-IV-60.1.fid
f1 (ppm)
OTBDPS
CH3
HO
CO2Me
7.26 CDCl3

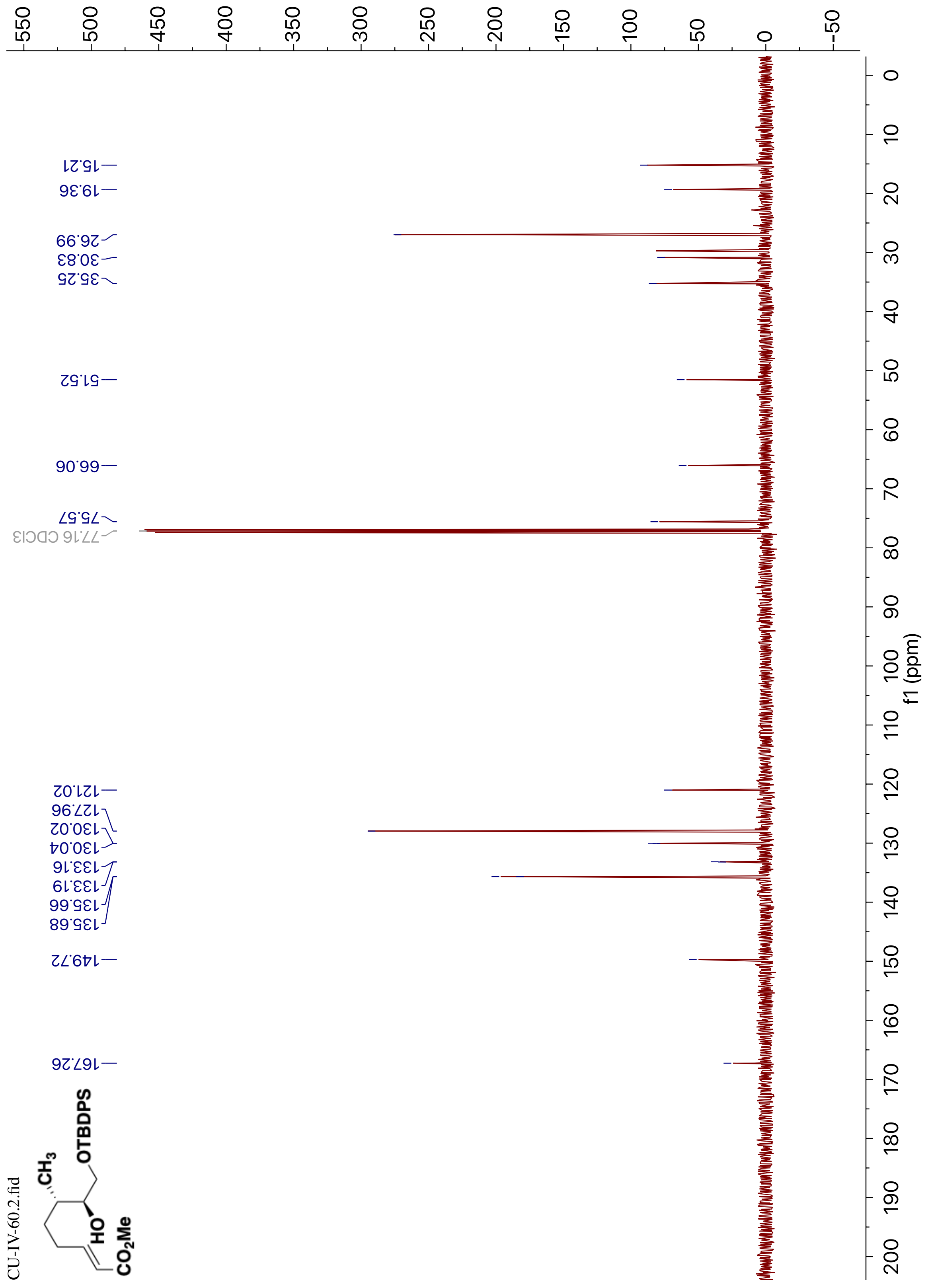
CU-IV-60.2.fid
OTBDPS
CH3
HO
CO2Me
15.21
19.36
26.99
30.83
35.25
51.52
66.06
75.57
77.16 CDCl3
121.02
127.96
130.02
130.04
133.16
133.19
135.66
135.68
149.72
167.26
f1 (ppm)

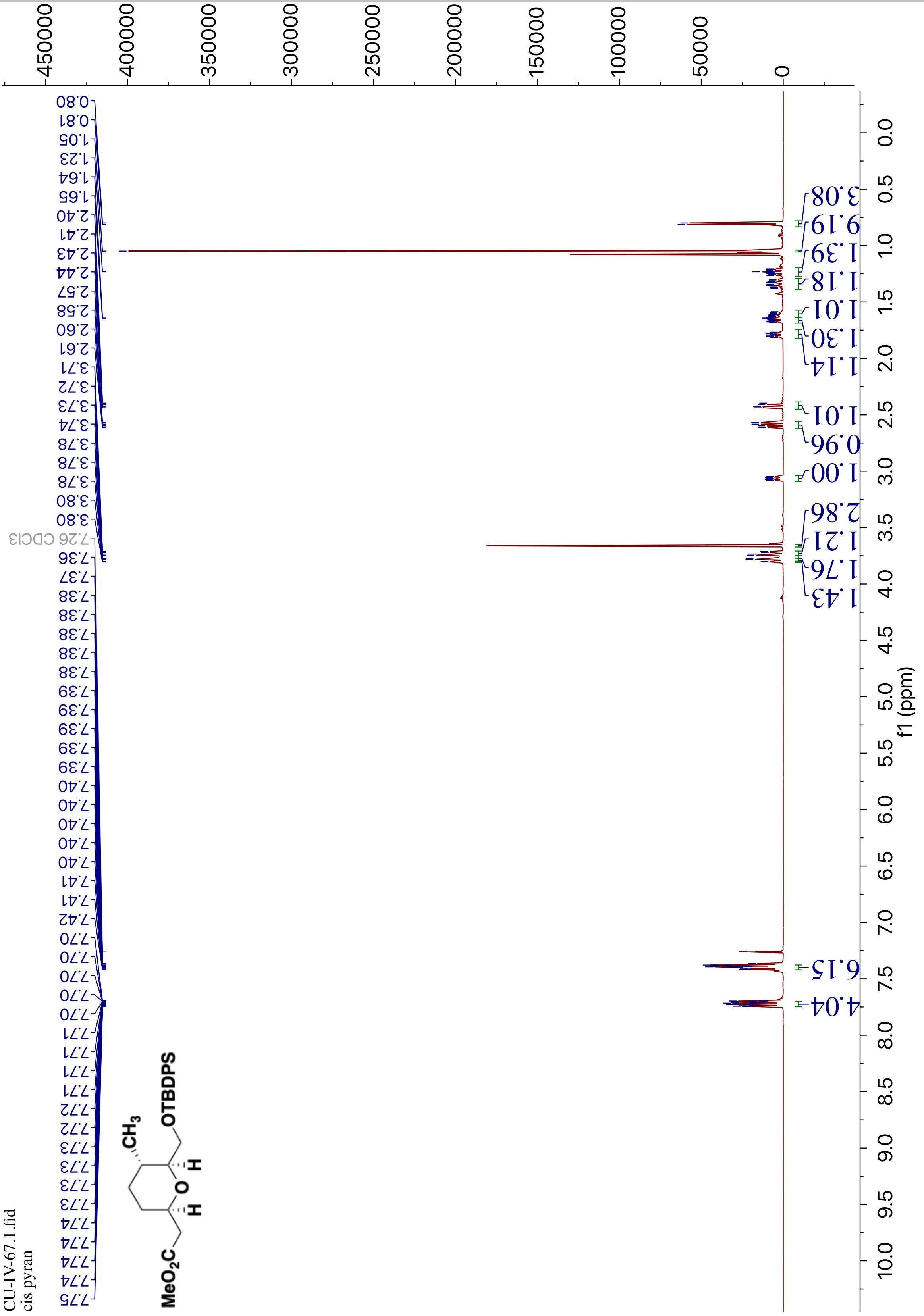
CU-IV-67.1.fid
cis pyran
MeO$_2$C
OTBDPS
CH$_3$
f1 (ppm)
7.26 CDCl3
3.08
9.19
1.39
1.18
1.01
1.30
1.14
1.01
0.96
1.00
2.86
1.21
1.76
1.43
6.15
4.04

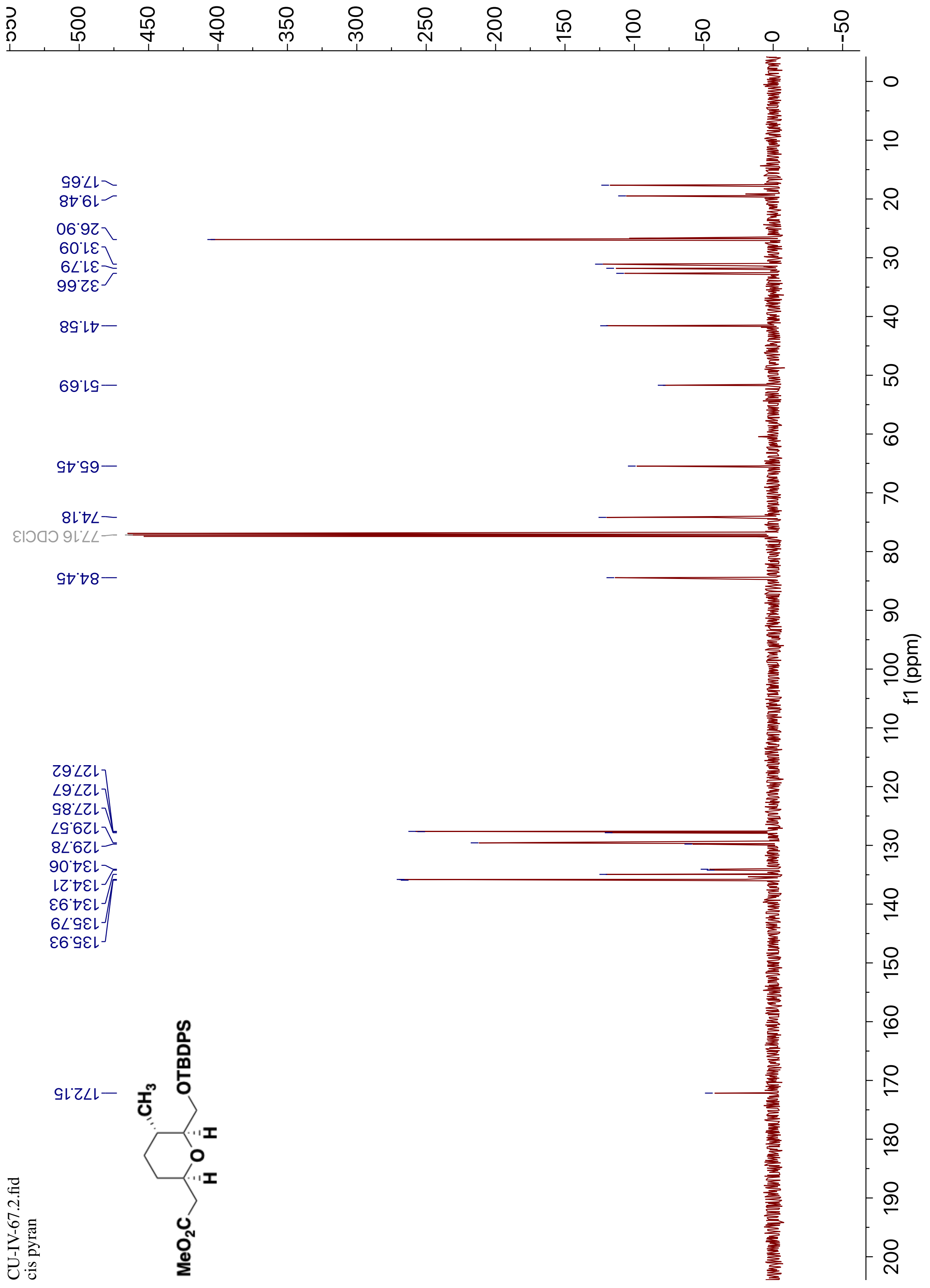
CU-IV-67.2.fid
cis pyran
17.65
19.48
26.90
31.09
31.79
32.66
41.58
51.69
65.45
74.18
77.16 CDCl3
84.45
127.62
127.67
127.85
129.57
129.78
134.06
134.21
134.93
135.79
135.93
172.15
MeO2C
H
O
H
CH3
OTBDPS
f1 (ppm)

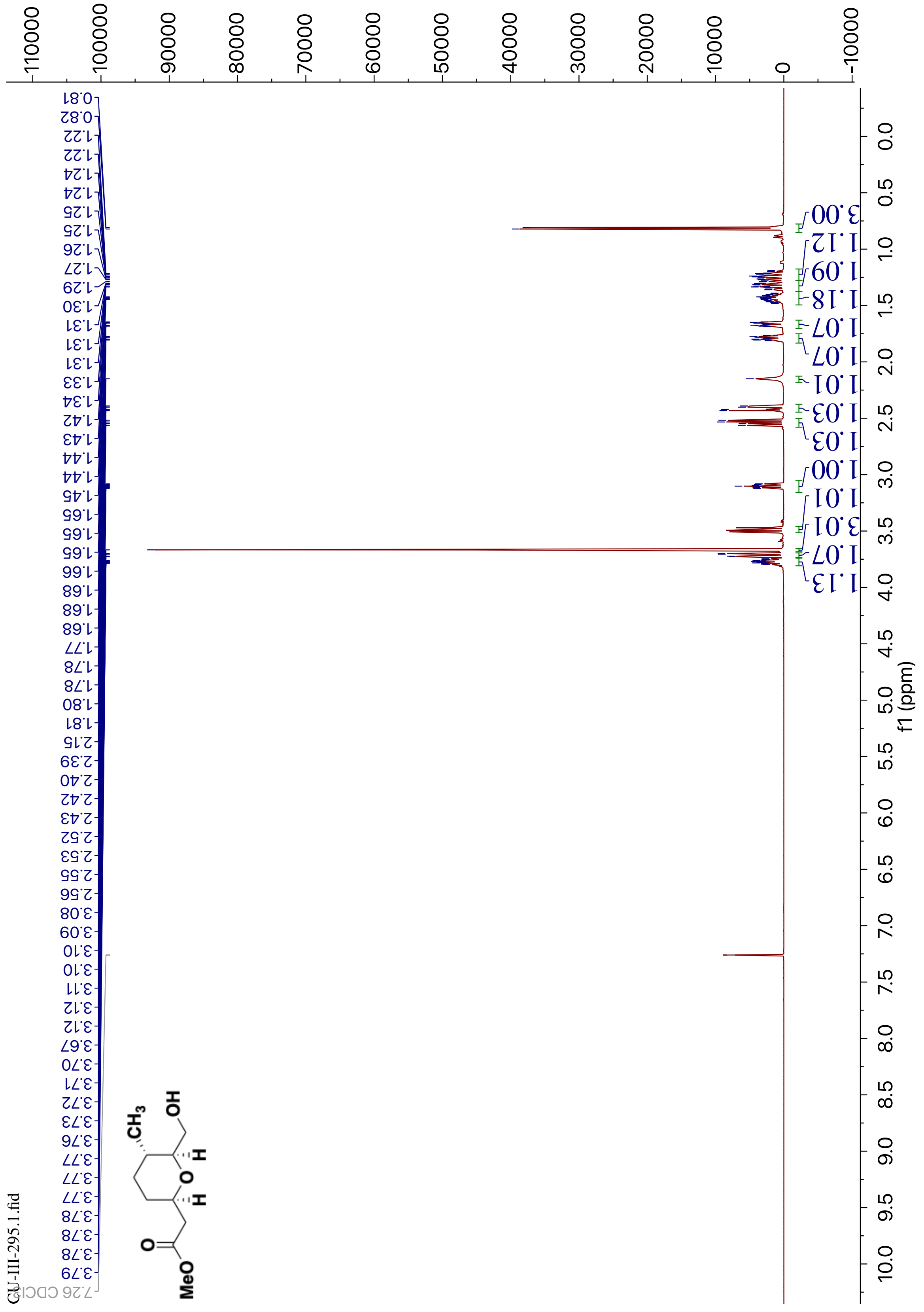
GU-III-295.1.fid
0.81
0.82
1.22
1.22
1.24
1.24
1.25
1.25
1.26
1.27
1.29
1.30
1.31
1.31
1.31
1.33
1.34
1.42
1.43
1.44
1.44
1.45
1.65
1.65
1.65
1.66
1.68
1.68
1.68
1.77
1.78
1.78
1.80
1.81
2.15
2.39
2.40
2.42
2.43
2.52
2.53
2.55
2.56
3.08
3.09
3.10
3.10
3.11
3.12
3.12
3.67
3.70
3.71
3.72
3.73
3.76
3.77
3.77
3.77
3.78
3.78
3.78
3.79
7.26 CDCl3
MeO
O
H
H
CH3
OH
3.00
1.12
1.09
1.18
1.07
1.07
1.01
1.03
1.03
1.00
1.01
3.01
1.07
1.13
f1 (ppm)

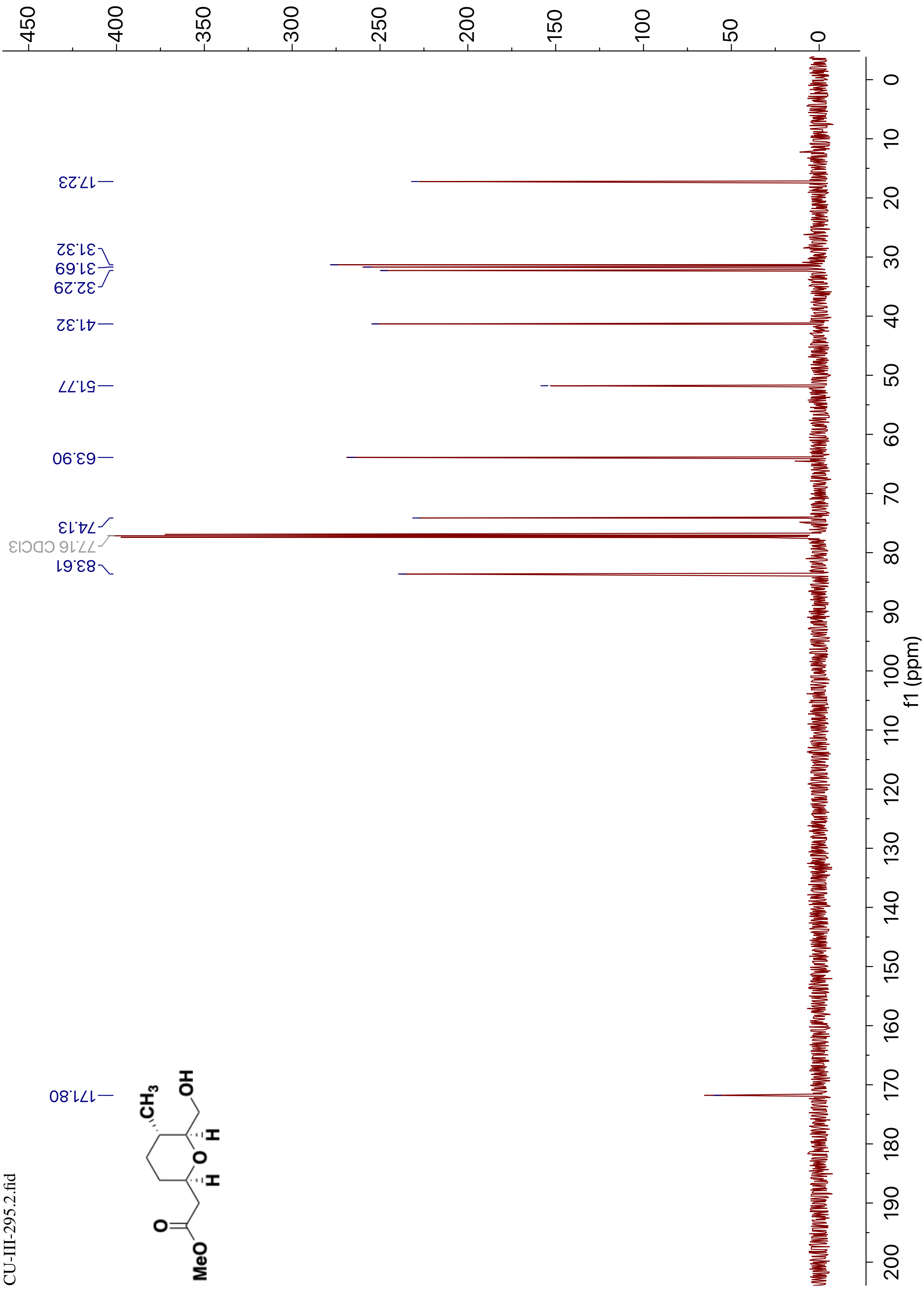
CU-III-295.2.fid
MeO
O
O
H
H
CH3
OH
171.80
83.61
77.16 CDCl3
74.13
63.90
51.77
41.32
32.29
31.69
31.32
17.23
f1 (ppm)
450
400
350
300
250
200
150
100
50
0
10
20
30
40
60
70
80
90
110
120
130
140
160
170
180
190

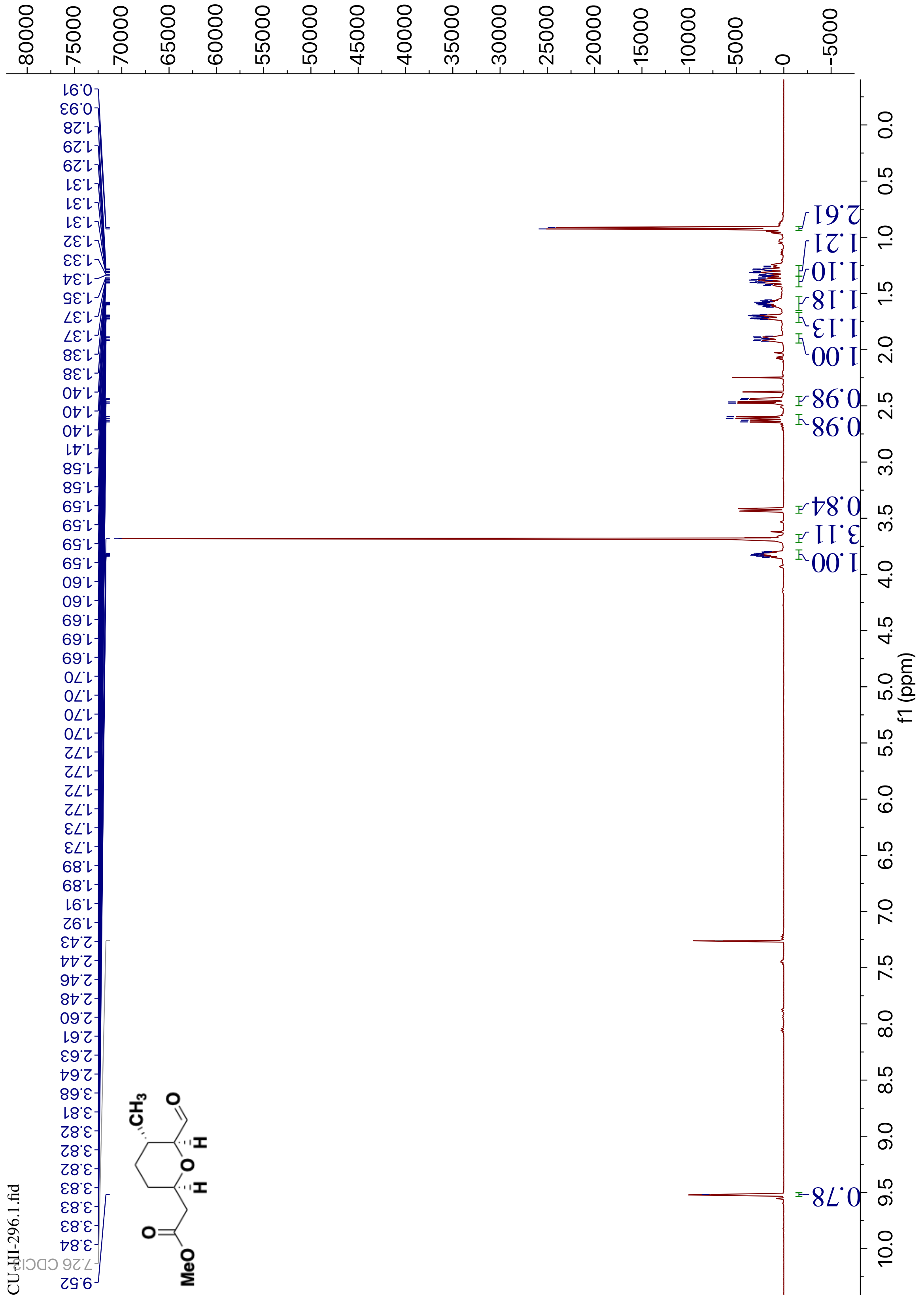
CU-III-296.1.fid
0.91 0.93 1.28 1.29 1.29 1.31 1.31 1.31 1.32 1.33 1.34 1.35 1.37 1.37 1.38 1.38 1.40 1.40 1.40 1.41 1.58 1.58 1.59 1.59 1.59 1.59 1.60 1.60 1.69 1.69 1.69 1.70 1.70 1.70 1.70 1.72 1.72 1.72 1.72 1.73 1.73 1.89 1.89 1.91 1.92 2.43 2.44 2.46 2.48 2.60 2.61 2.63 2.64 3.68 3.81 3.82 3.82 3.82 3.83 3.83 3.83 3.84 7.26 CDCl3 9.52
2.61 1.21 1.10 1.18 1.13 1.00 0.98 0.98 0.84 3.11 1.00 0.78
f1 (ppm)
MeO
O
CH3
H
H

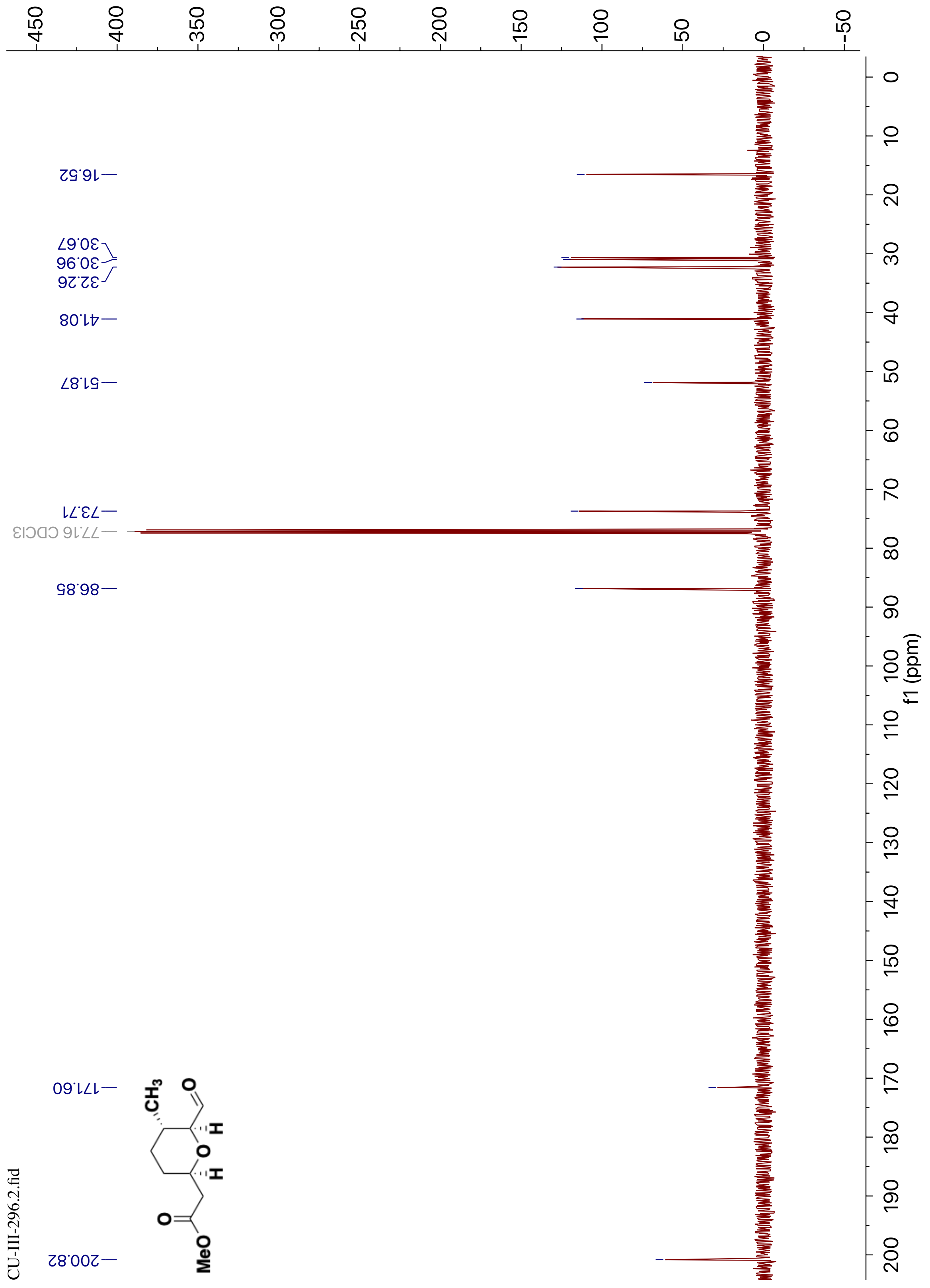
CU-III-296.2.fid
200.82
171.60
86.85
77.16 CDCl3
73.71
51.87
41.08
32.26
30.96
30.67
16.52
MeO
O
H
H
CH3
f1 (ppm)

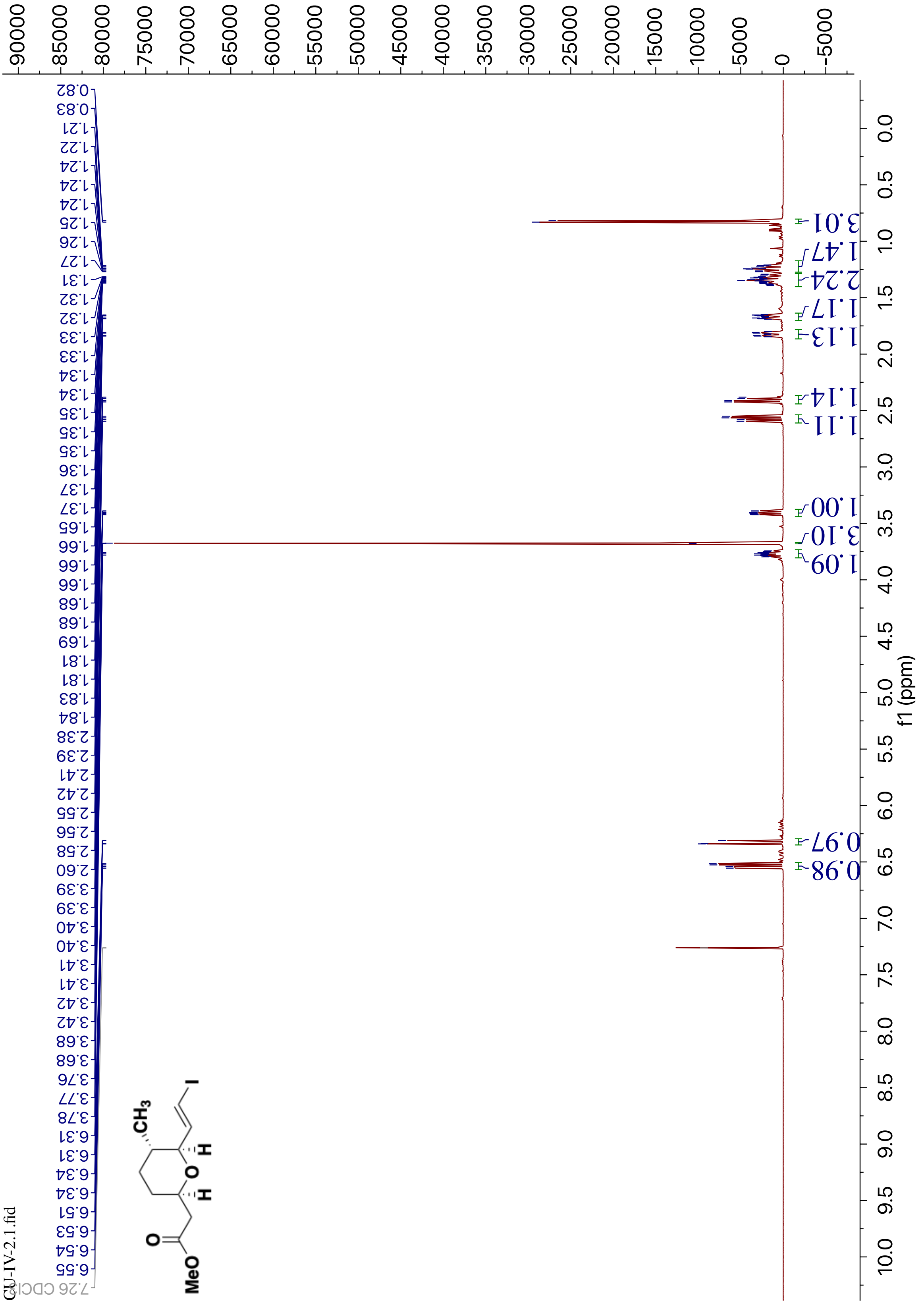
f1 (ppm)

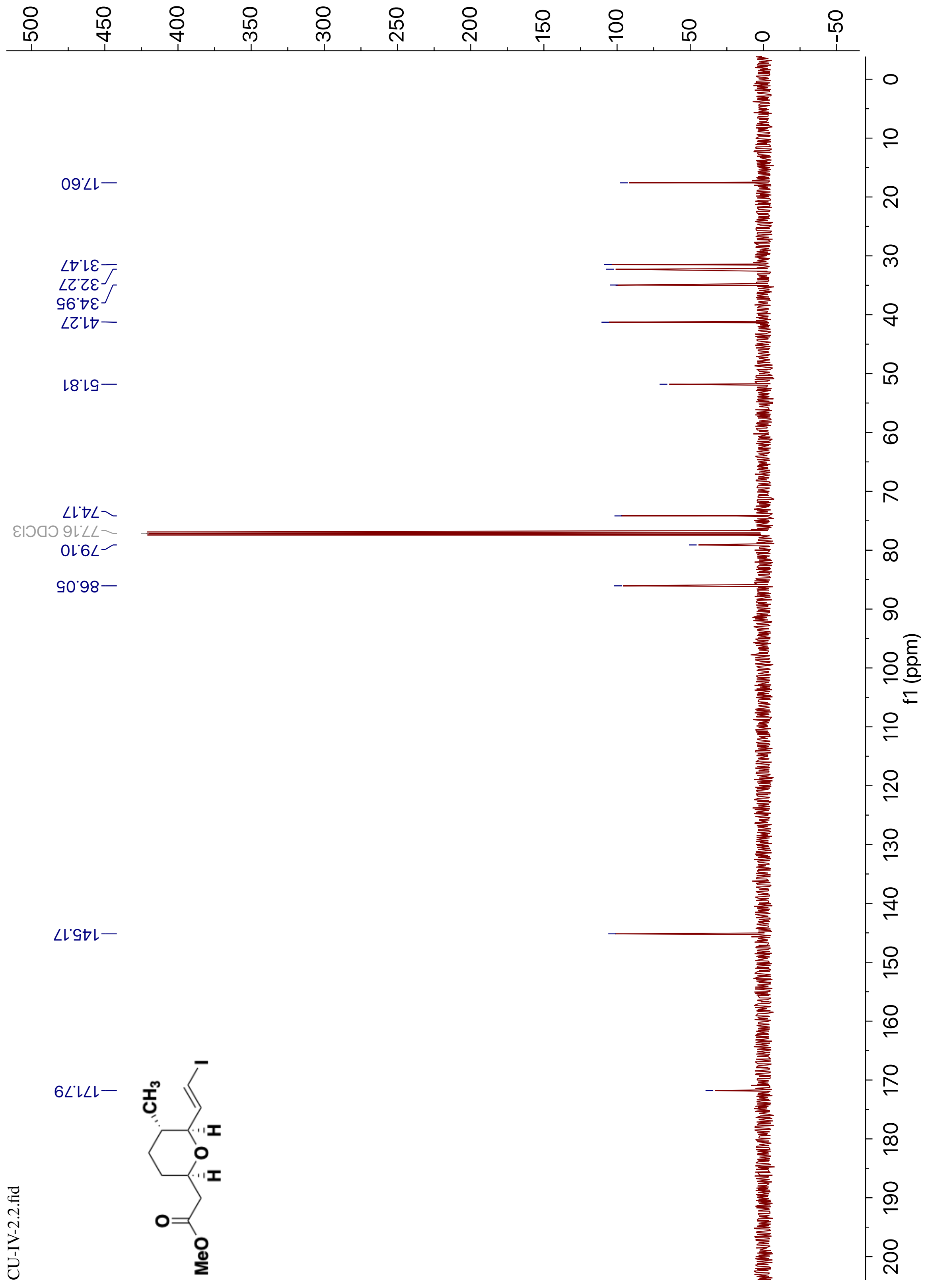
CU-IV-2.2.fid
17.60
31.47
32.27
34.95
41.27
51.81
74.17
77.16 CDCl3
79.10
86.05
145.17
171.79
MeO
CH3
I
f1 (ppm)

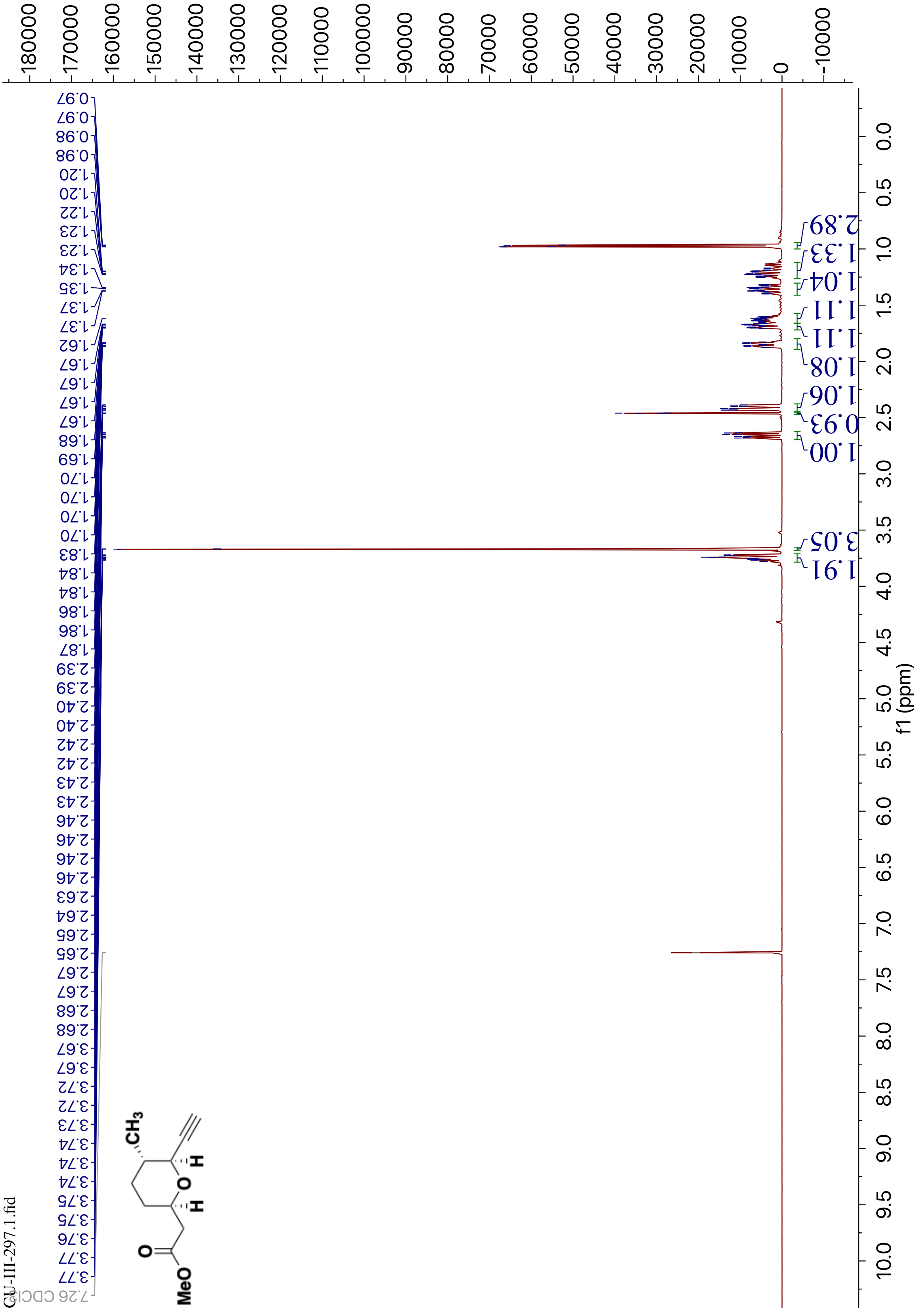
GU-III-297.1.fid
0.97
0.97
0.98
0.98
1.20
1.20
1.22
1.23
1.23
1.34
1.35
1.37
1.37
1.62
1.67
1.67
1.67
1.67
1.68
1.69
1.70
1.70
1.70
1.70
1.83
1.84
1.84
1.86
1.86
1.87
2.39
2.39
2.40
2.40
2.42
2.42
2.43
2.43
2.46
2.46
2.46
2.46
2.63
2.64
2.65
2.65
2.67
2.67
2.68
2.68
3.67
3.67
3.72
3.72
3.73
3.74
3.74
3.74
3.75
3.75
3.76
3.77
3.77
7.26 CDCl3
2.89
1.33
1.04
1.11
1.11
1.08
1.06
0.93
1.00
3.05
1.91
f1 (ppm)
MeO
O
H
CH3

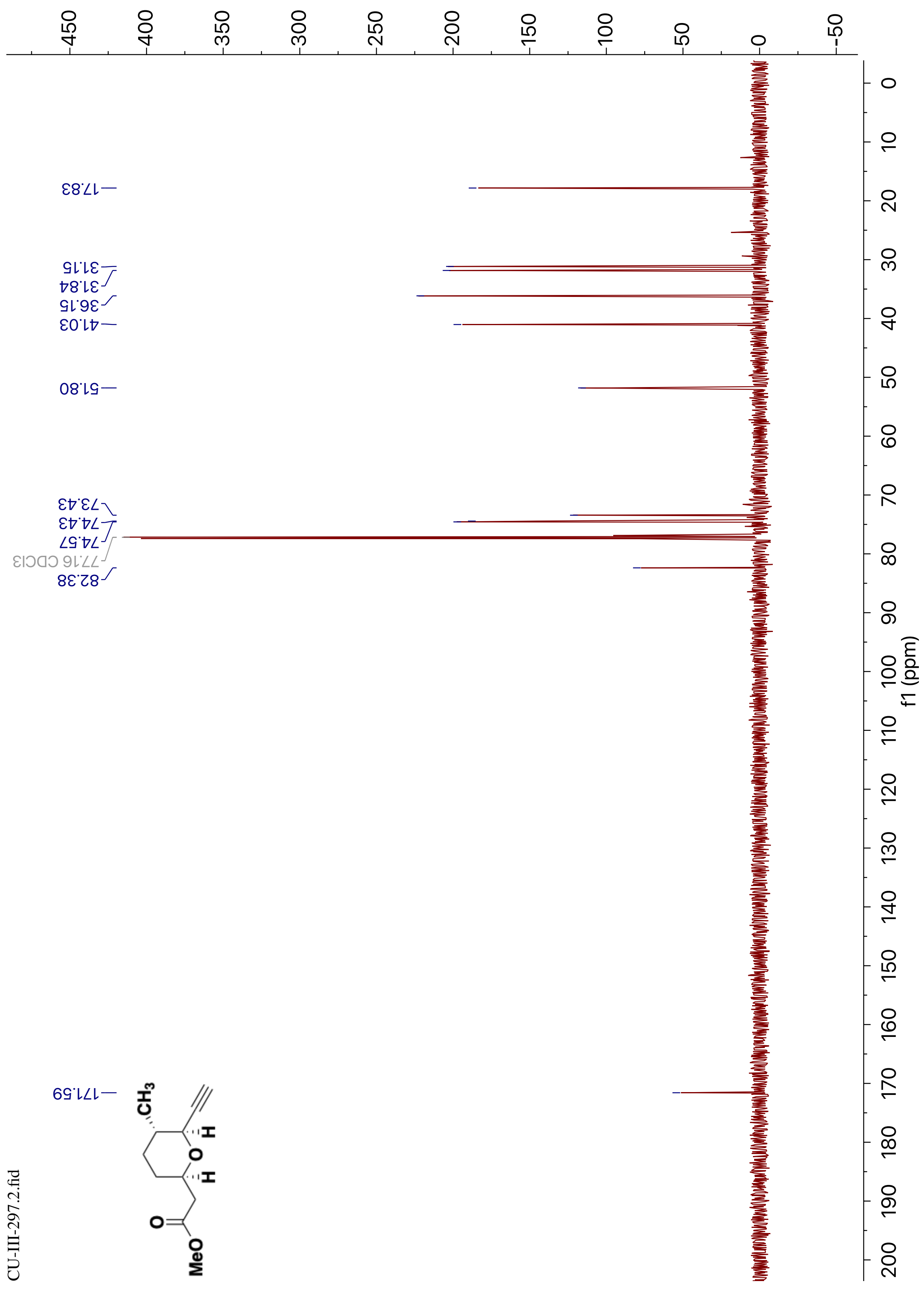
CU-III-297.2.fid
17.83
31.15
31.84
36.15
41.03
51.80
73.43
74.43
74.57
77.16 CDCl3
82.38
171.59
MeO
O
H
CH3
f1 (ppm)

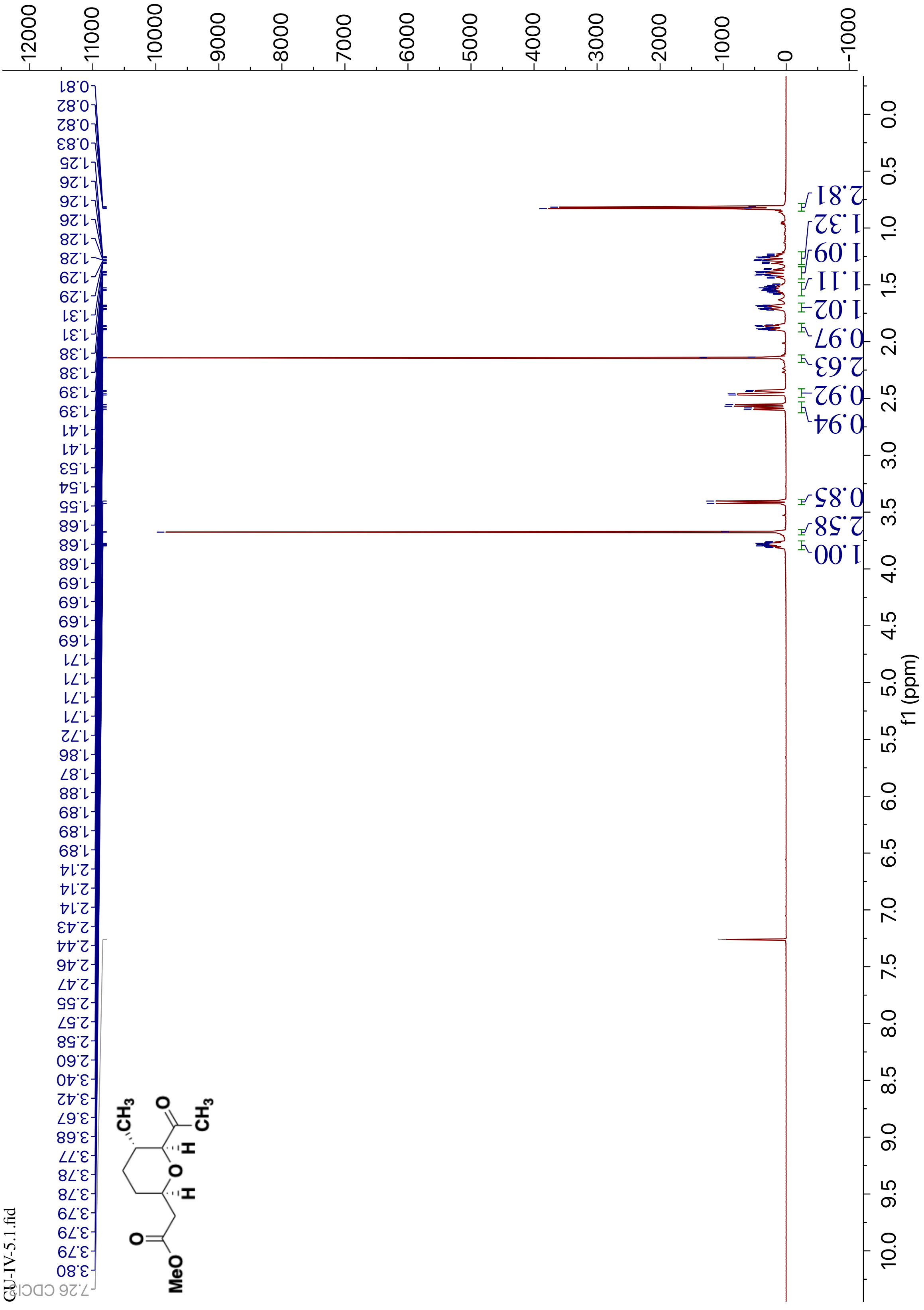
CU-IV-5.1.fid
f1 (ppm)
7.26 CDCl3
MeO

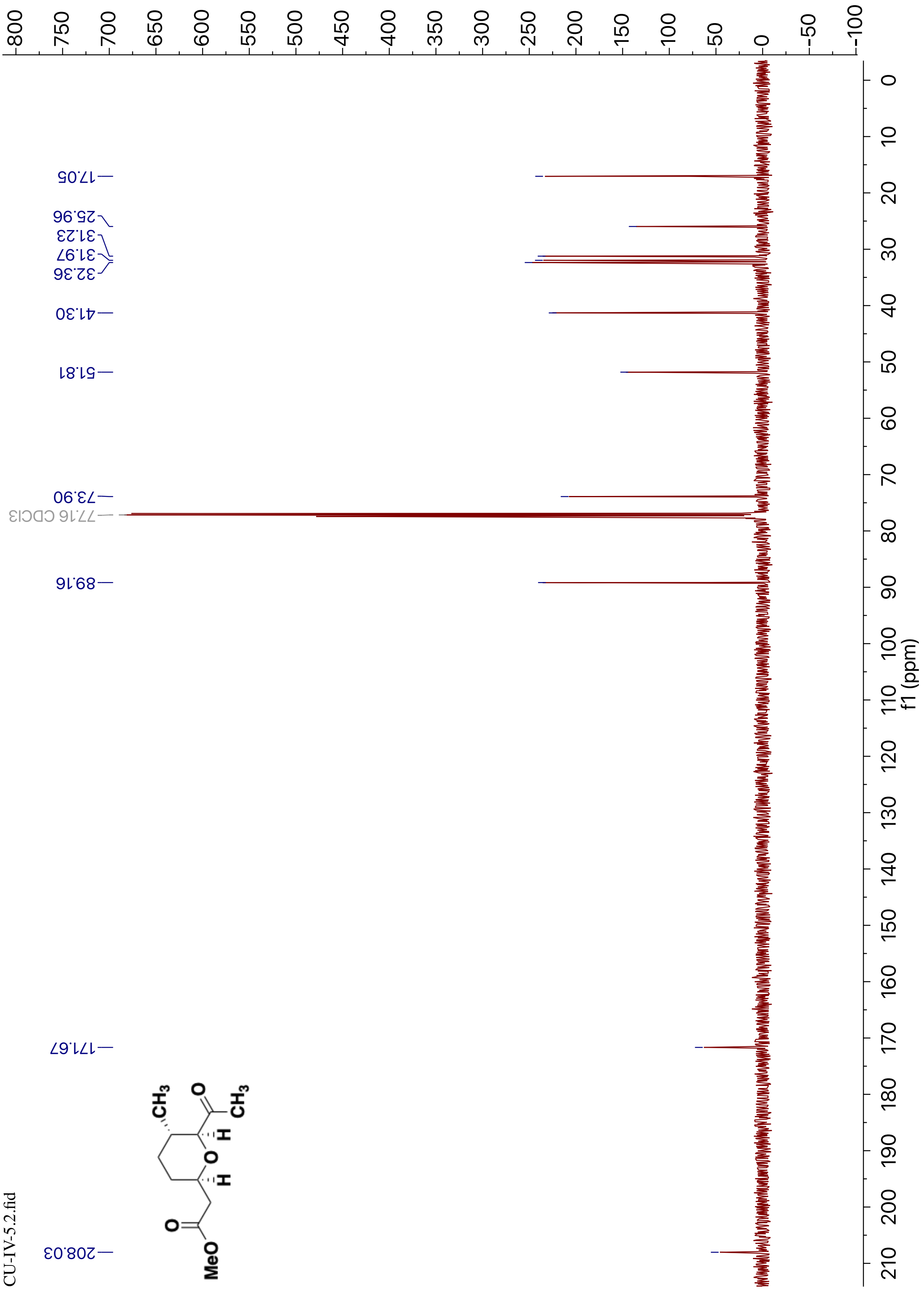
CU-IV-5.2.fid
208.03
171.67
89.16
77.16 CDCl3
73.90
51.81
41.30
32.36
31.97
31.23
25.96
17.05
MeO
O
H
H
CH3
f1 (ppm)

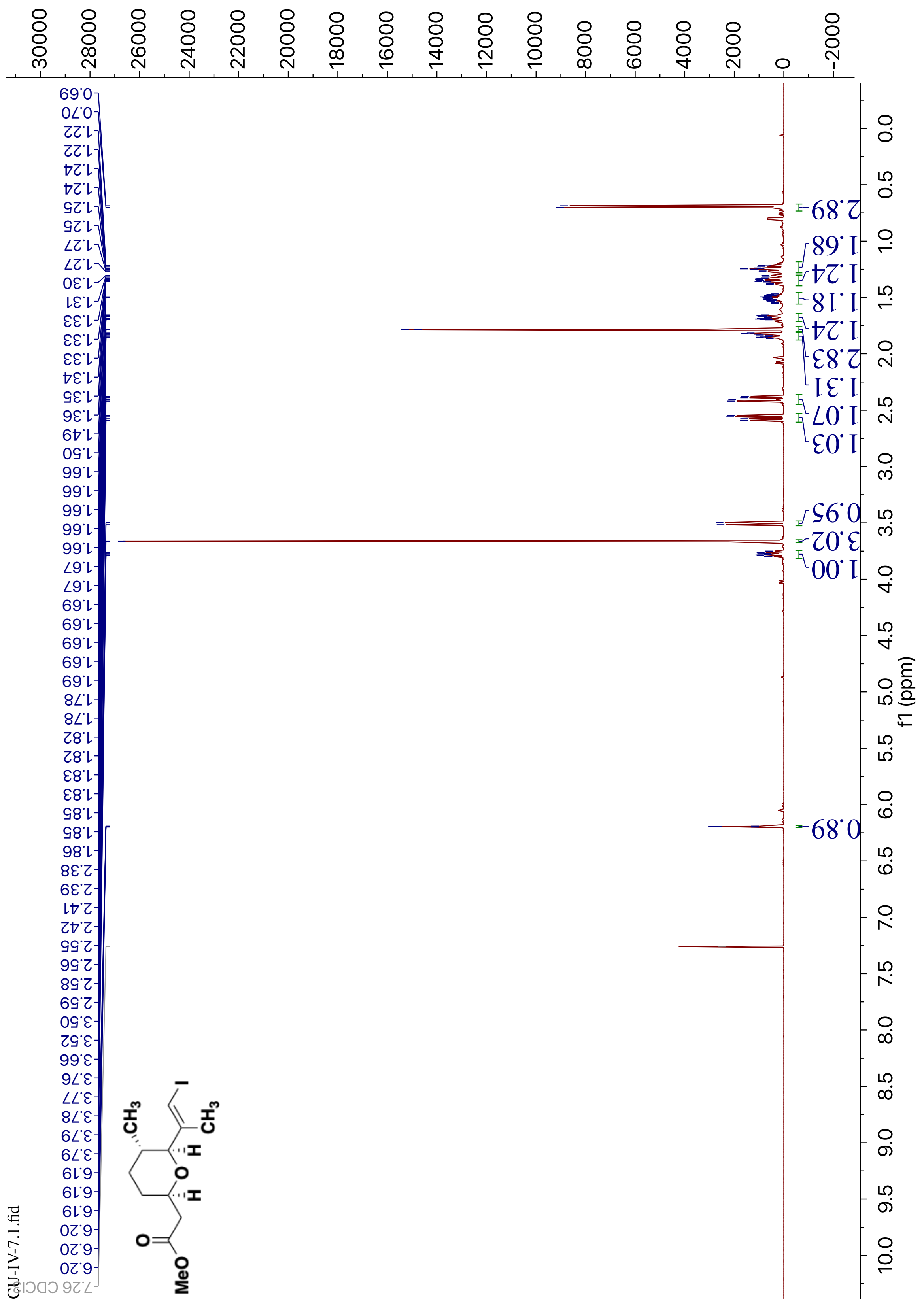
CU-IV-7.1.fid
f1 (ppm)
7.26 CDCl3

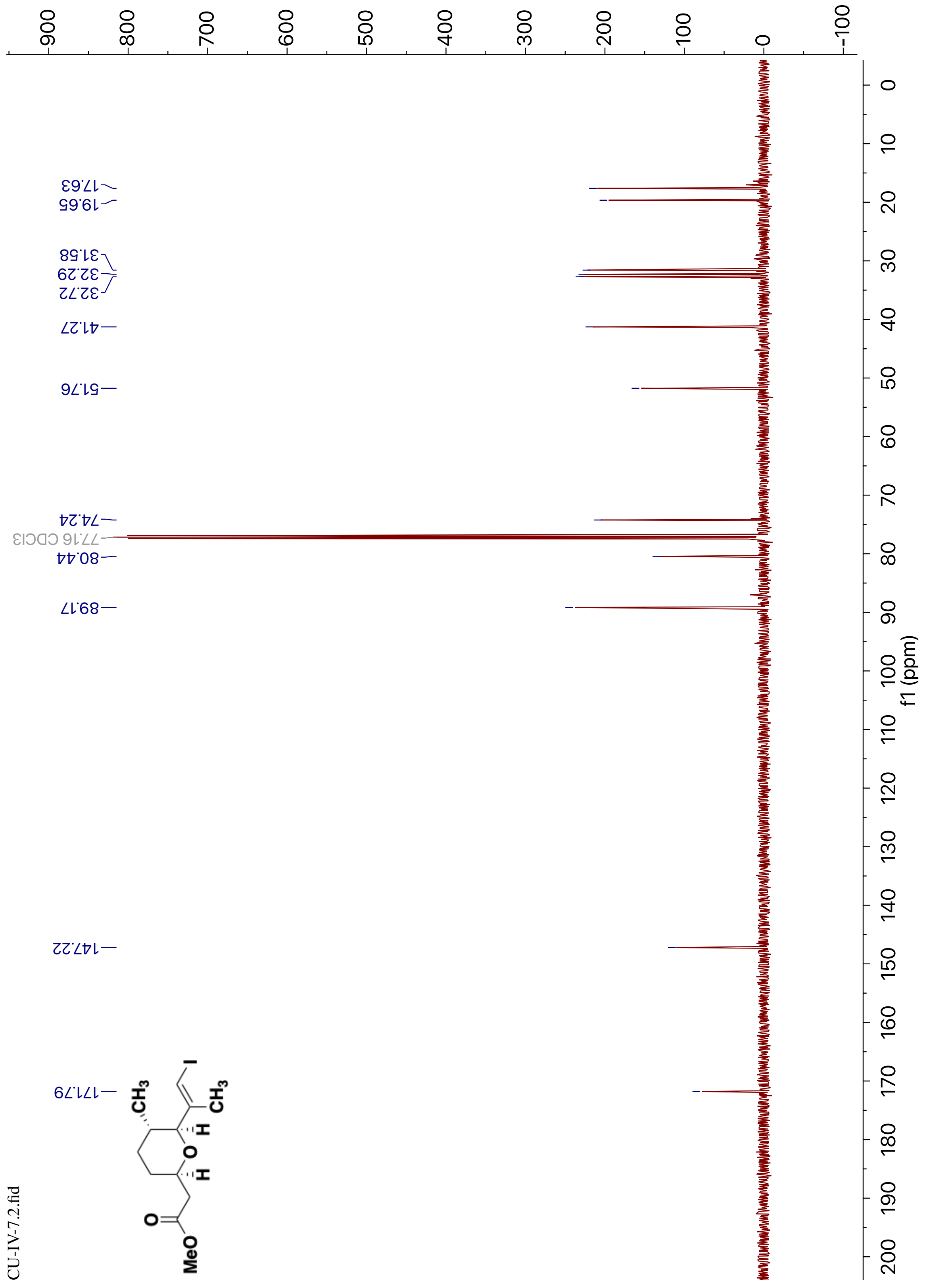
CU-IV-7.2.fid
17.63
19.65
31.58
32.29
32.72
41.27
51.76
74.24
77.16 CDCl3
80.44
89.17
147.22
171.79
f1 (ppm)

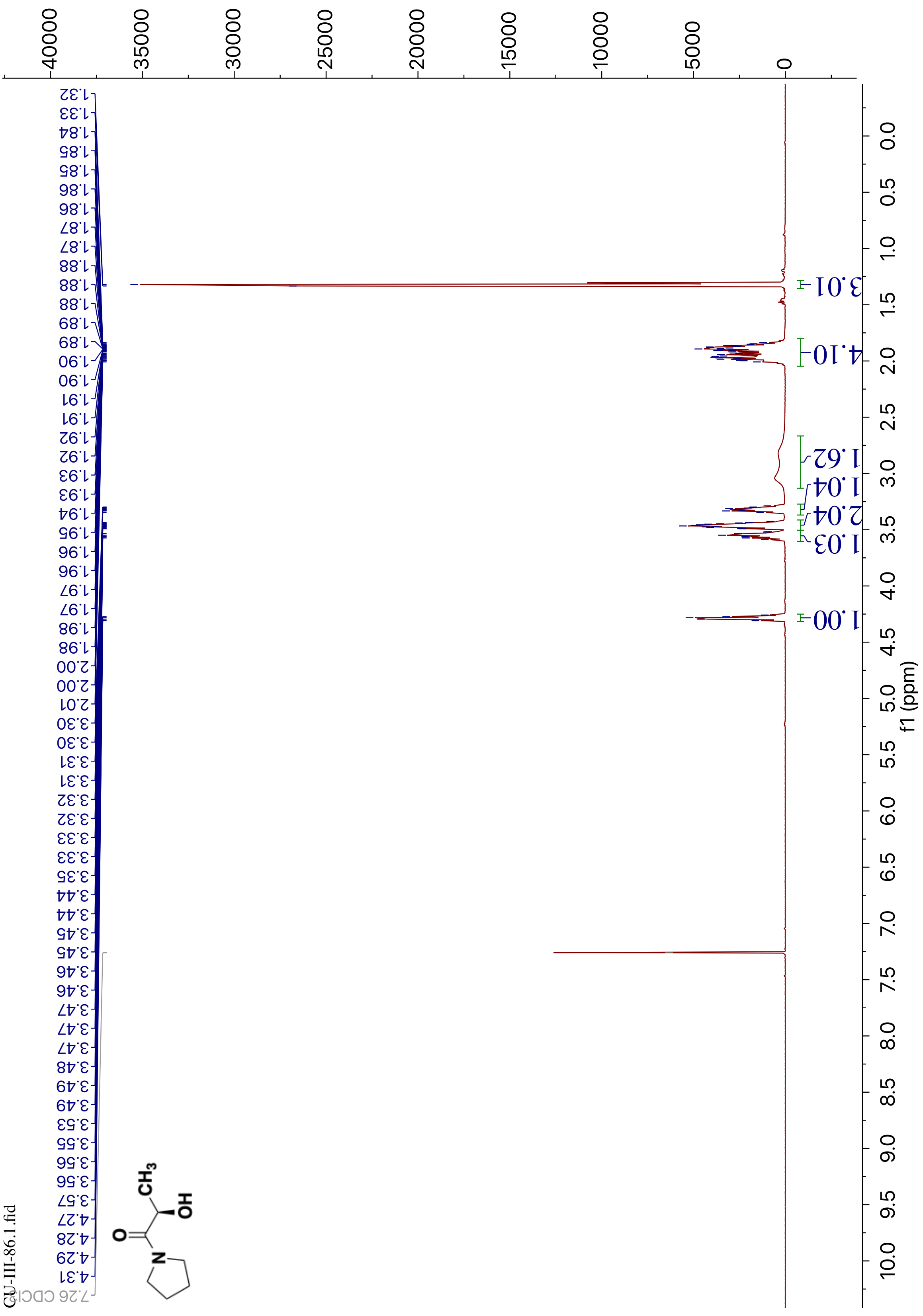
GU-III-86.1.fid
1.32
1.33
1.84
1.85
1.85
1.86
1.86
1.87
1.87
1.88
1.88
1.88
1.89
1.89
1.90
1.90
1.91
1.91
1.92
1.92
1.93
1.93
1.94
1.95
1.96
1.96
1.97
1.97
1.98
1.98
2.00
2.00
2.01
3.30
3.30
3.31
3.31
3.32
3.32
3.33
3.33
3.35
3.44
3.44
3.45
3.45
3.46
3.46
3.47
3.47
3.47
3.48
3.49
3.49
3.53
3.55
3.56
3.56
3.57
4.27
4.28
4.29
4.31
7.26 CDCl3
3.01
4.10
1.62
1.04
2.04
1.03
1.00
f1 (ppm)

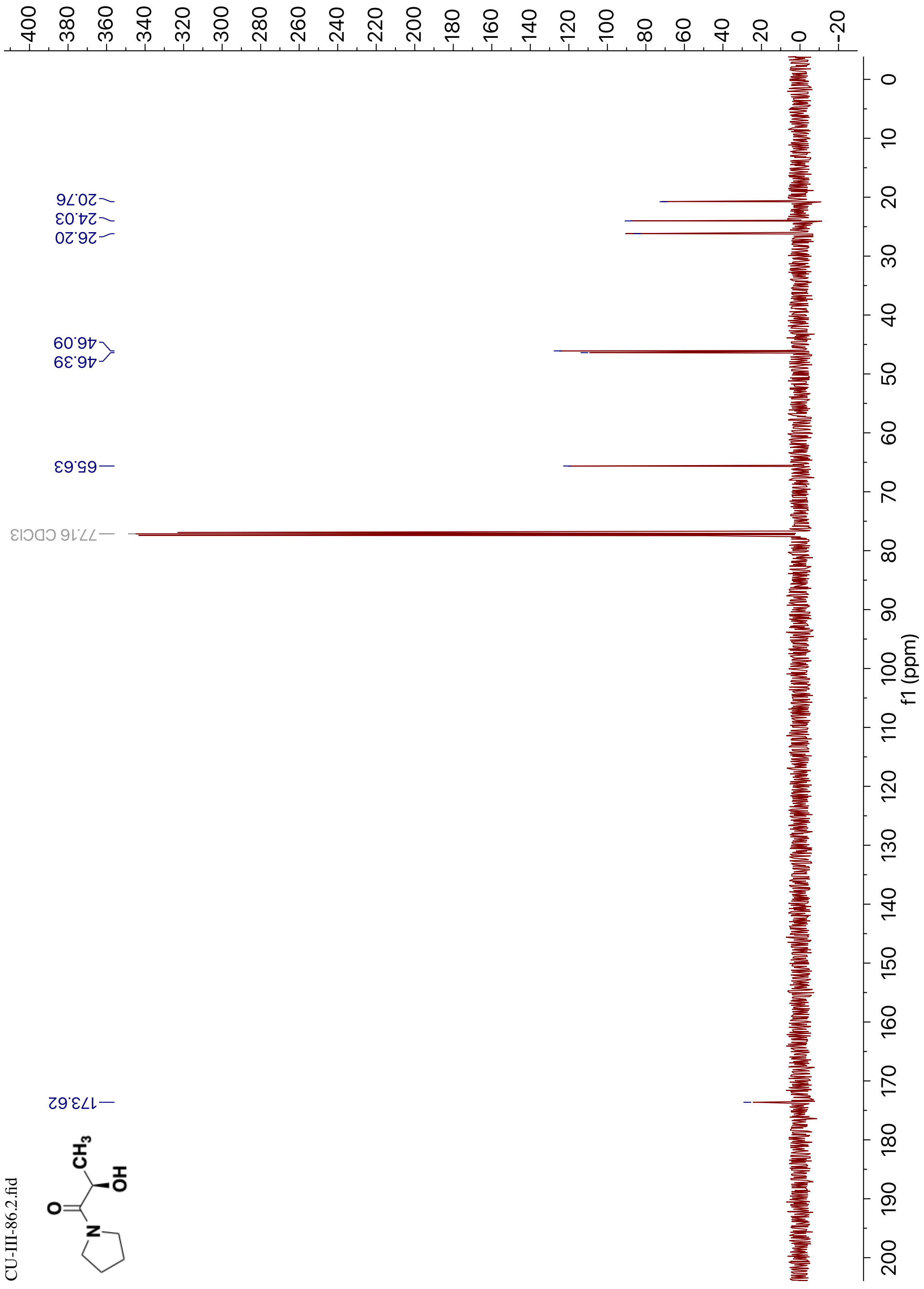
CU-III-86.2.fid
20.76
24.03
26.20
46.09
46.39
65.63
77.16 CDCl3
173.62
f1 (ppm)

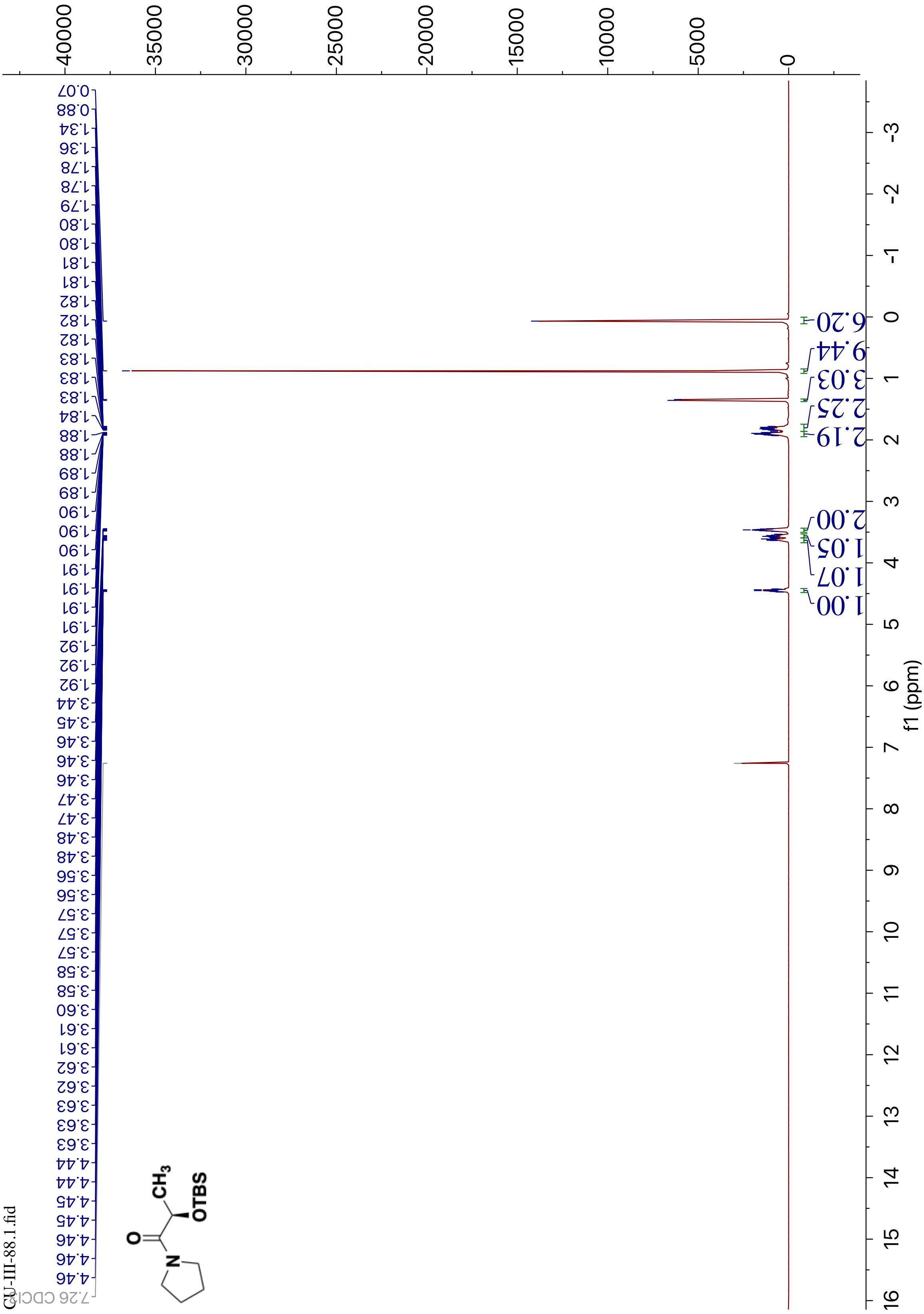
f1 (ppm)
OTBS
CH3
O
N

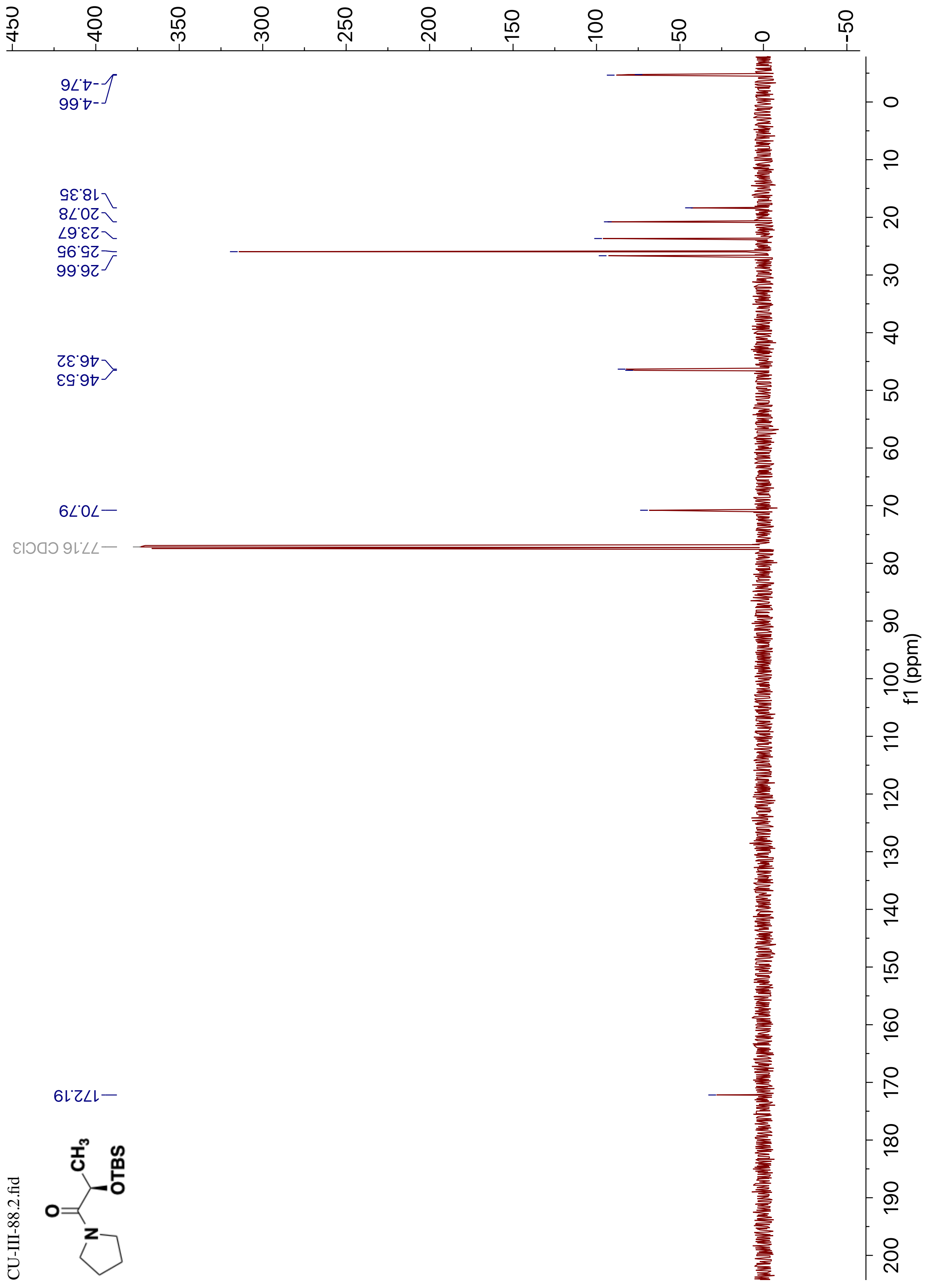
CU-III-88.2.fid
-4.76
-4.66
18.35
20.78
23.67
25.95
26.66
46.32
46.53
70.79
77.16 CDCl3
172.19
O
N
CH3
OTBS
f1 (ppm)

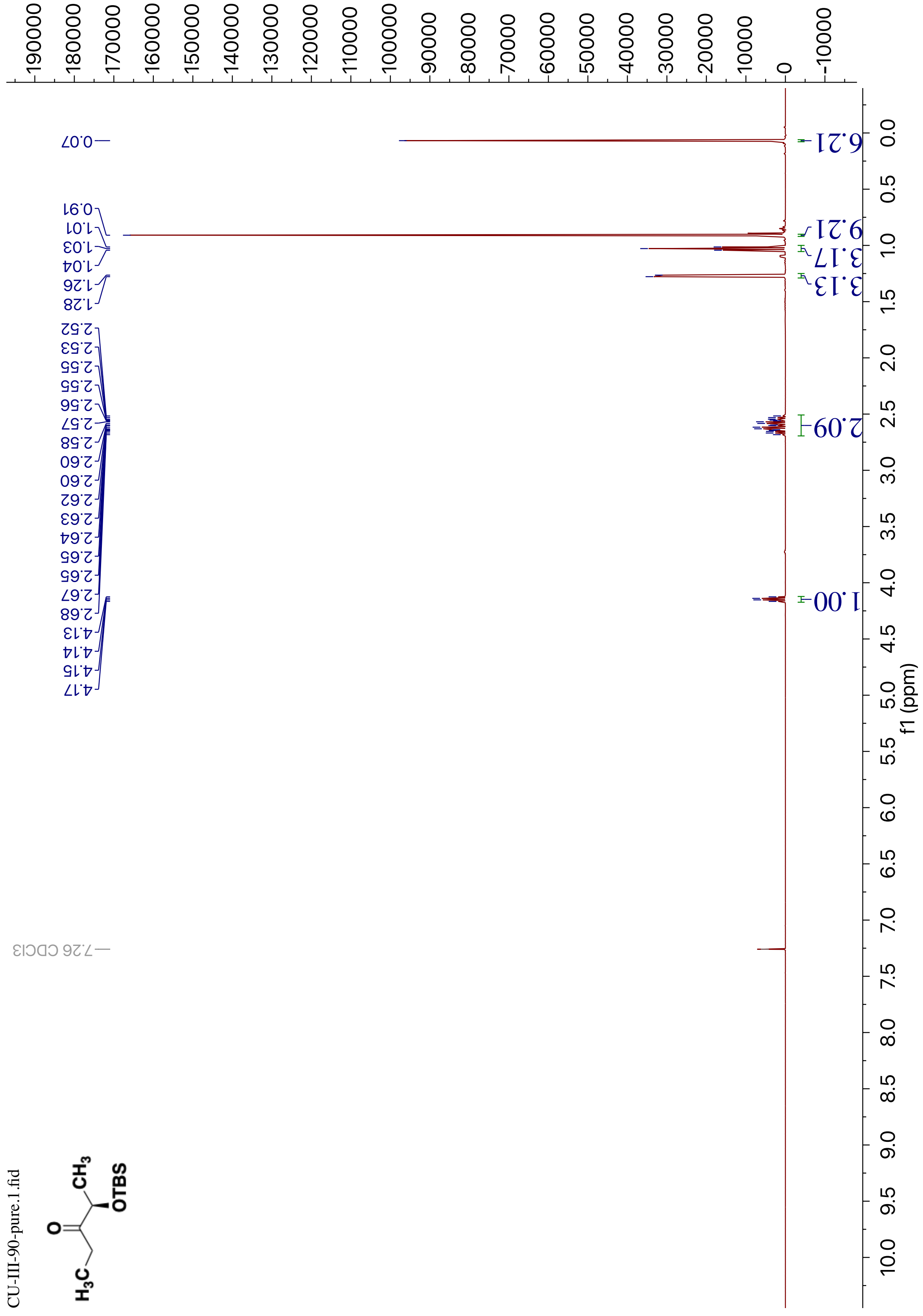
CU-III-90-pure.1.fid
OTBS
f1 (ppm)
7.26 CDCl3
6.21
9.21
3.17
3.13
2.09
1.00

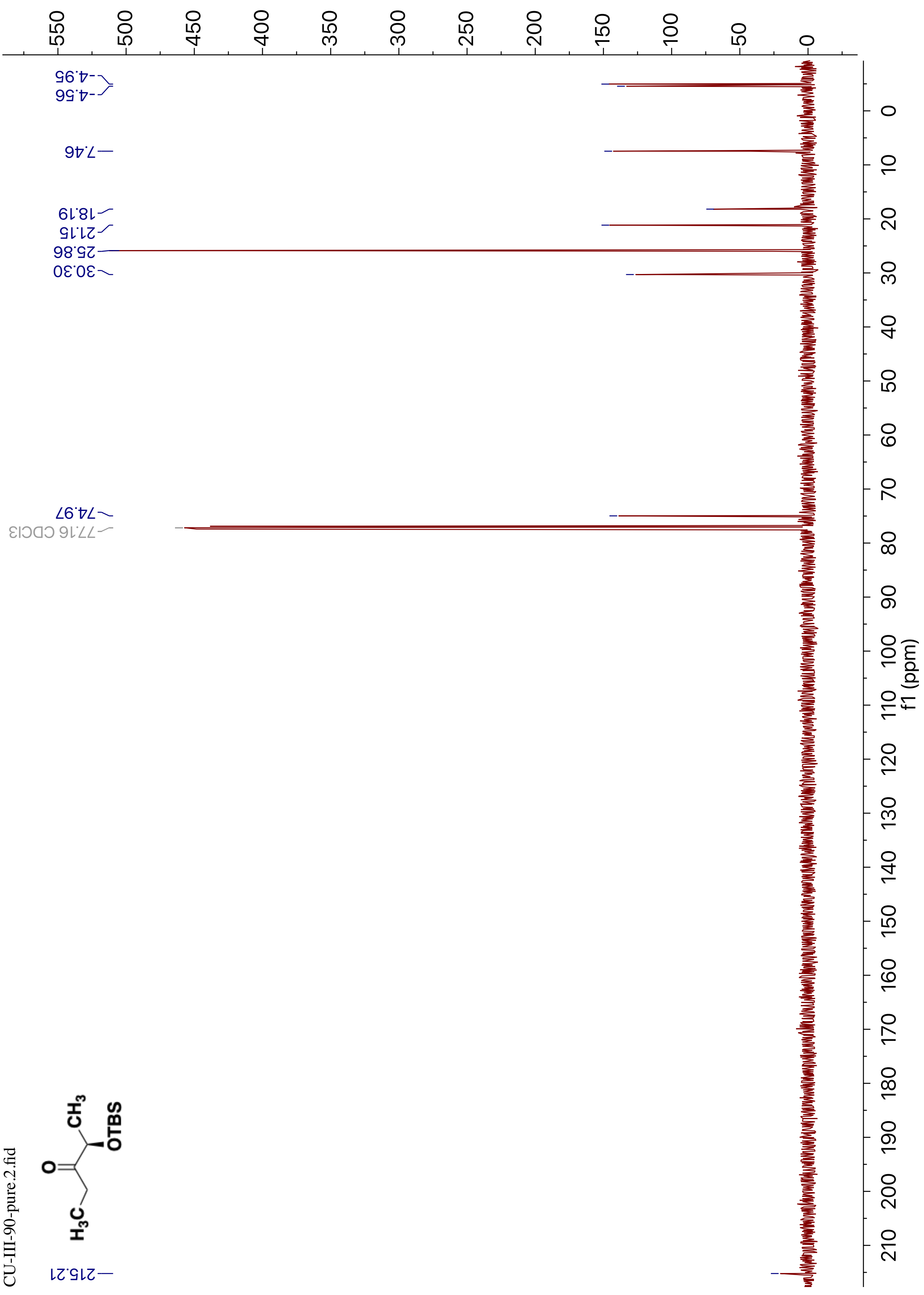
CU-III-90-pure.2.fid
OTBS
-4.95
-4.56
7.46
18.19
21.15
25.86
30.30
74.97
77.16 CDCl3
215.21
f1 (ppm)

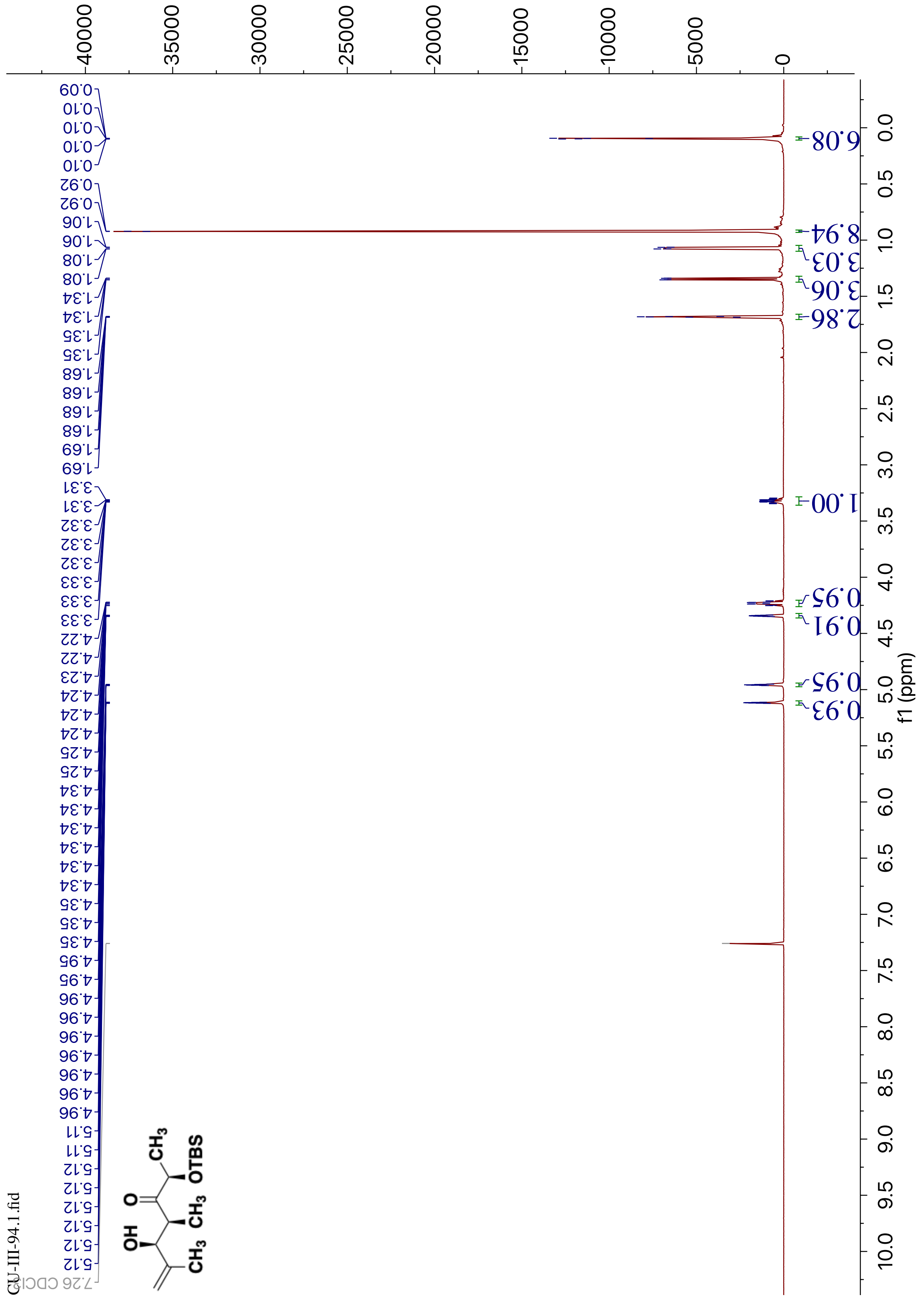
CU-III-94.1.fid
7.26 CDCl3
f1 (ppm)
OH O
CH3 CH3 OTBS
CH3

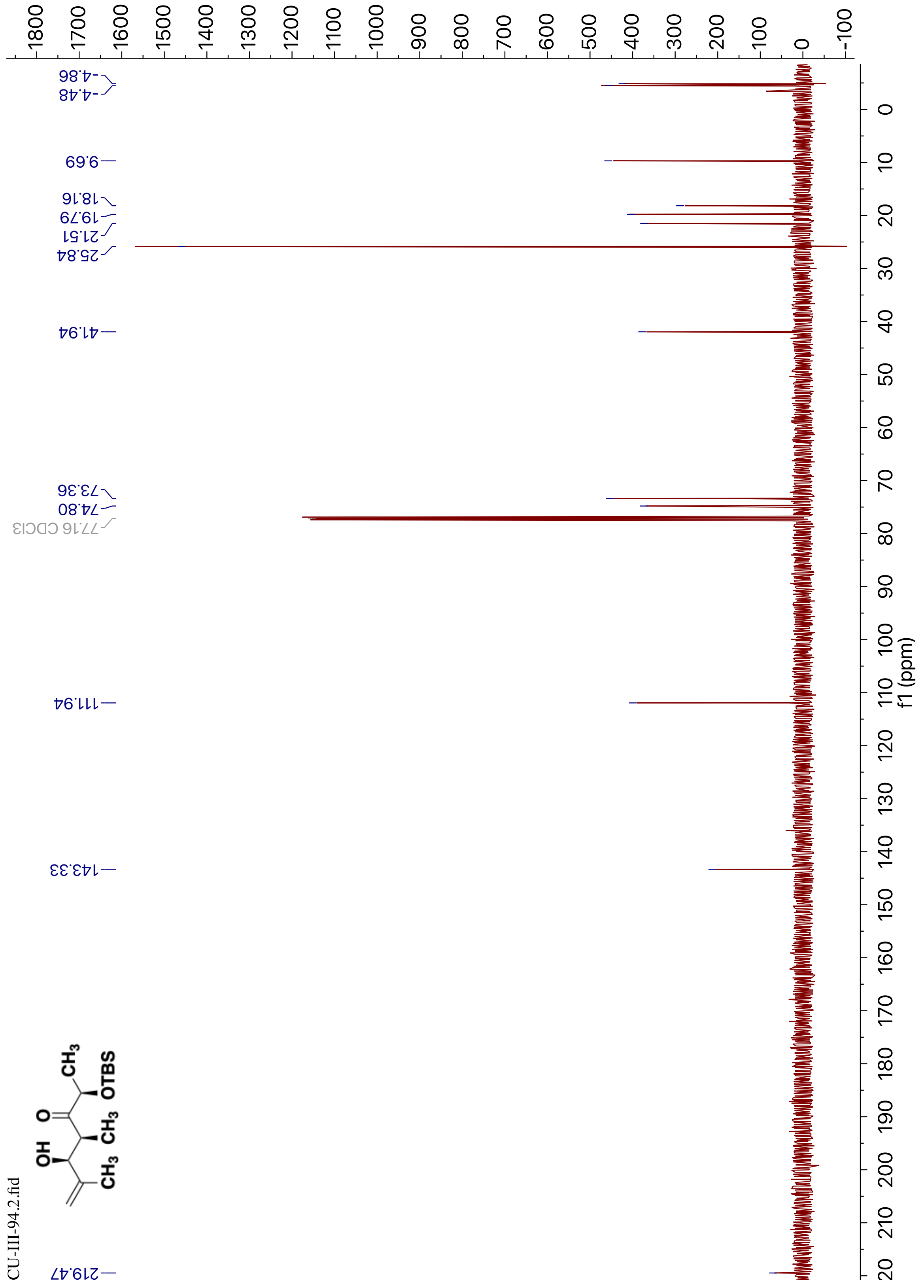
CU-III-94.2.fid
-4.86
-4.48
9.69
18.16
19.79
21.51
25.84
41.94
73.36
74.80
77.16 CDCl3
111.94
143.33
219.47
f1 (ppm)

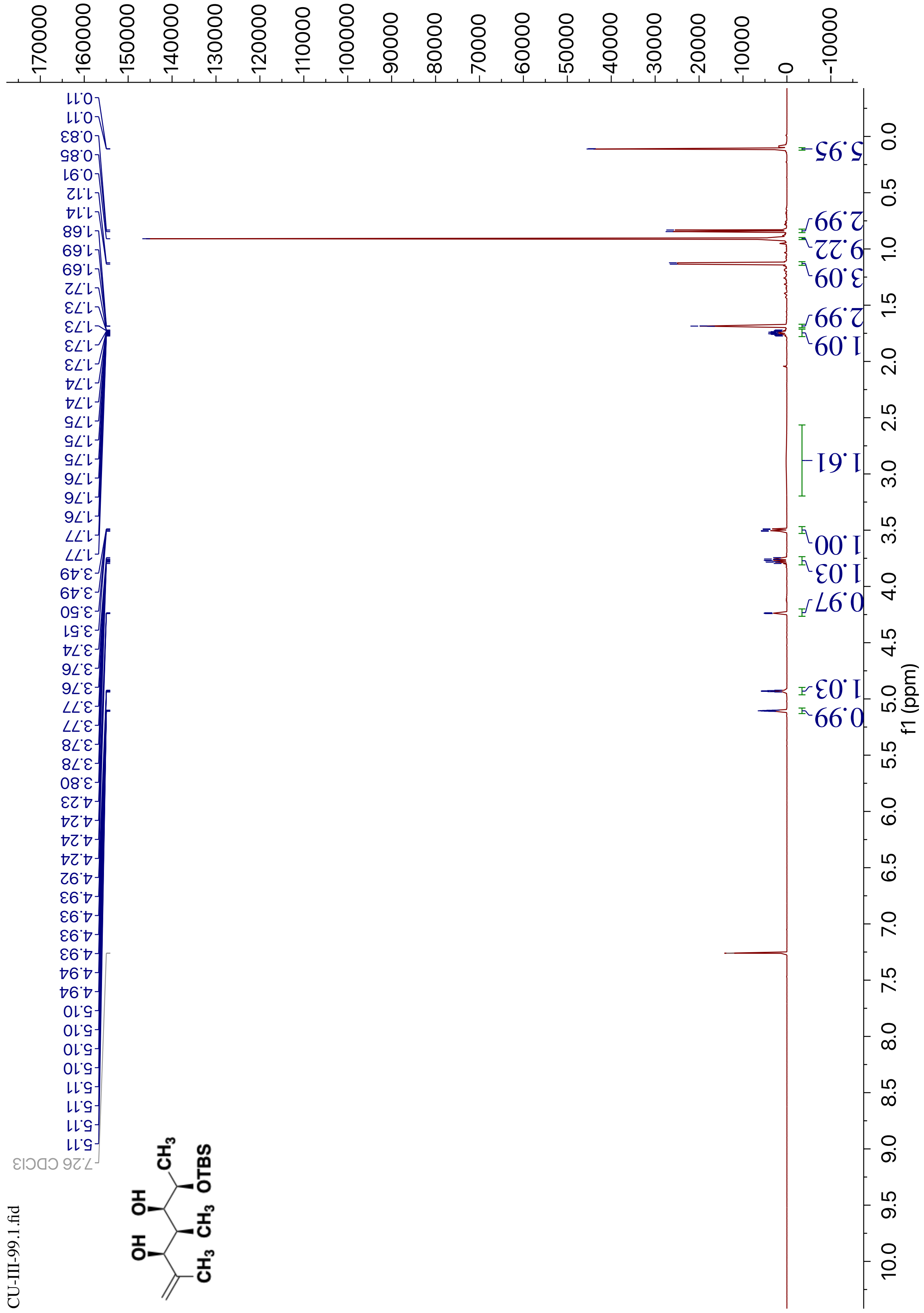
CU-III-99.1.fid
0.11
0.11
0.83
0.85
0.91
1.12
1.14
1.68
1.69
1.69
1.72
1.73
1.73
1.73
1.73
1.74
1.74
1.75
1.75
1.75
1.76
1.76
1.76
1.77
1.77
3.49
3.49
3.50
3.51
3.74
3.76
3.76
3.77
3.77
3.78
3.78
3.80
4.23
4.24
4.24
4.24
4.92
4.93
4.93
4.93
4.93
4.94
4.94
5.10
5.10
5.10
5.10
5.11
5.11
5.11
5.11
7.26 CDCl3
OH
OH
CH3
CH3
CH3
OTBS
5.95
2.99
9.22
3.09
2.99
1.09
1.61
1.00
1.03
0.97
1.03
0.99
f1 (ppm)

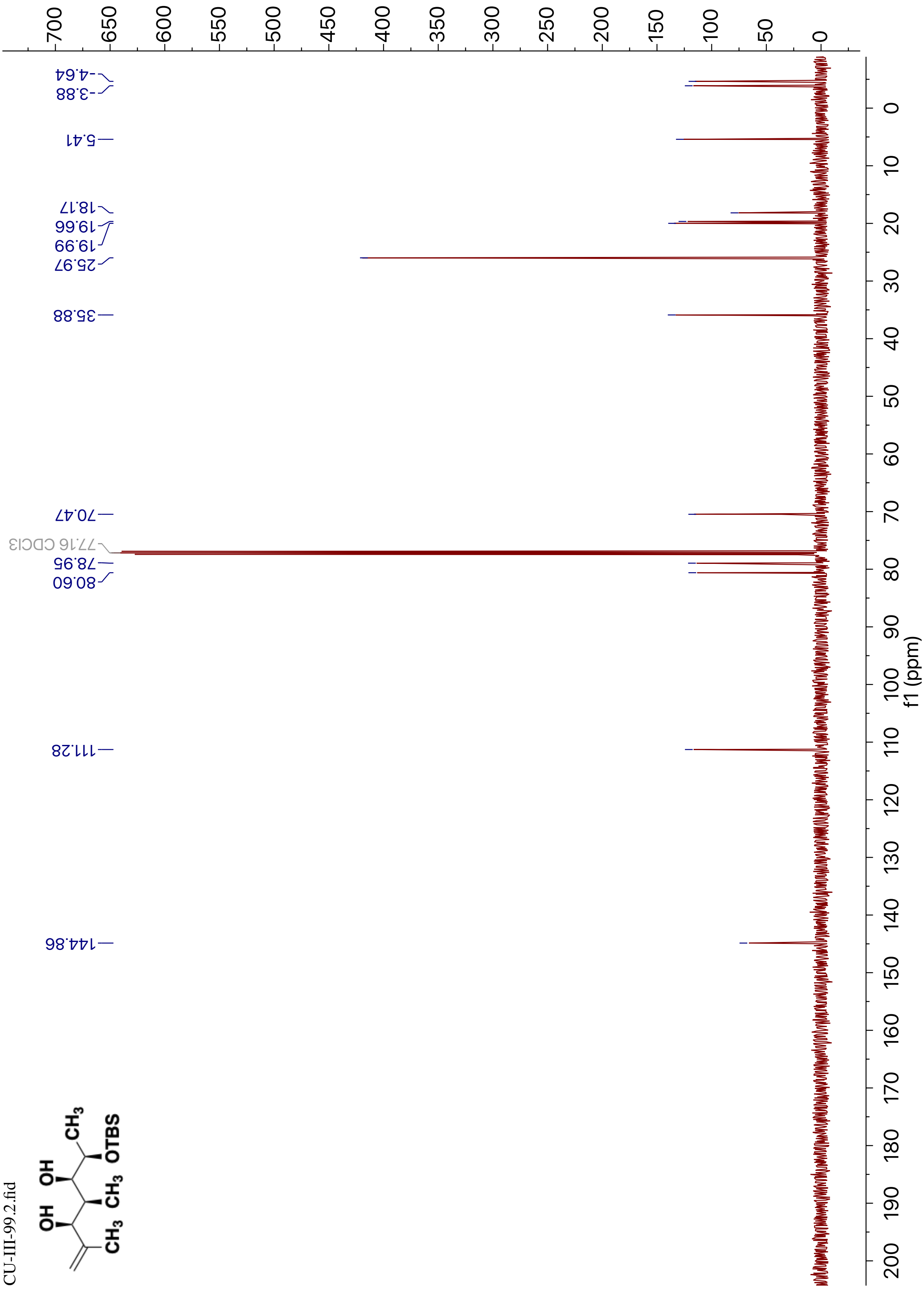
CU-III-99.2.fid
OH OH
CH3 CH3 OTBS
CH3
-4.64
-3.88
5.41
18.17
19.66
19.99
25.97
35.88
70.47
77.16 CDCl3
78.95
80.60
111.28
144.86
f1 (ppm)

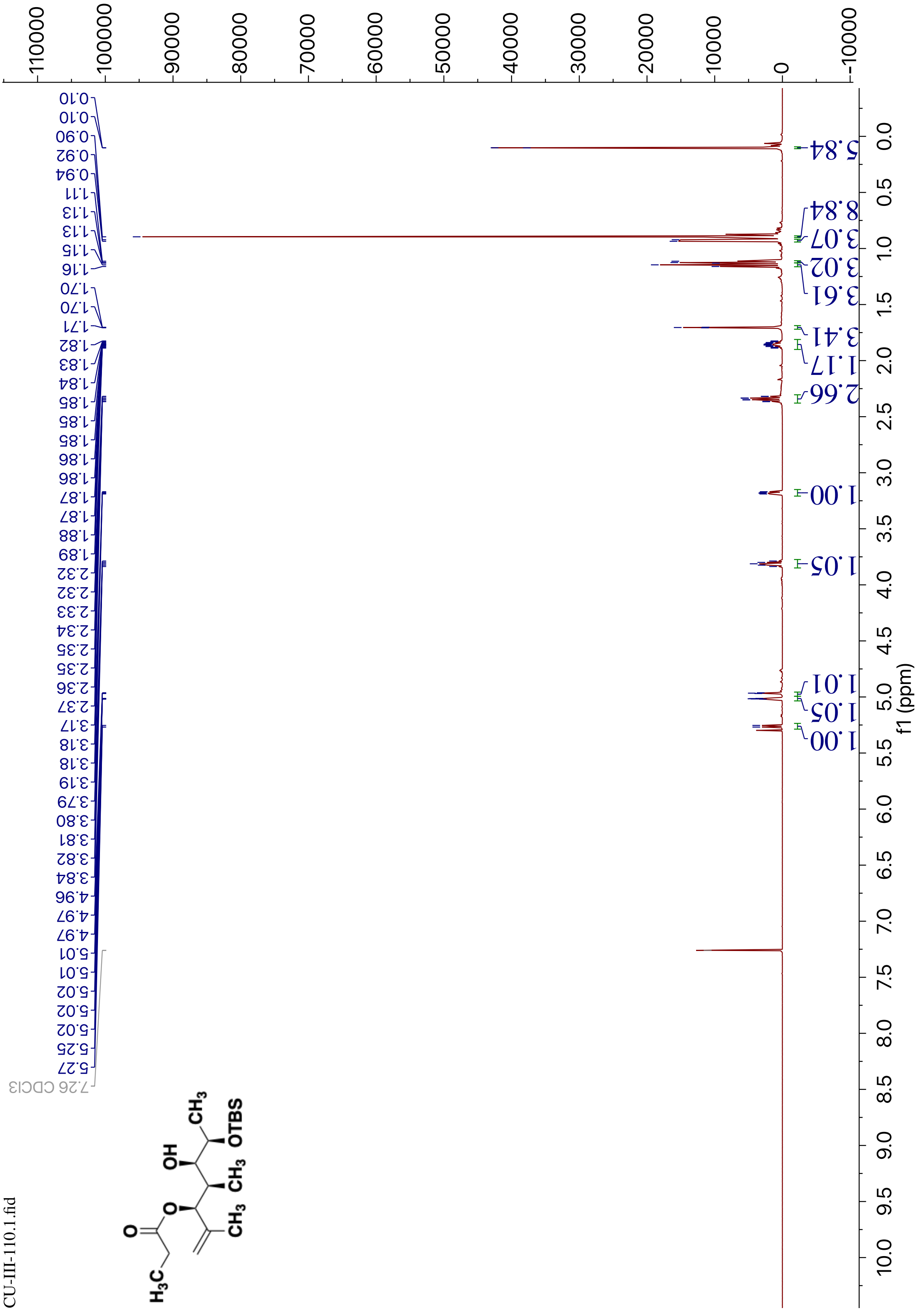
CU-III-110.1.fid
f1 (ppm)
7.26 CDCl3
OTBS

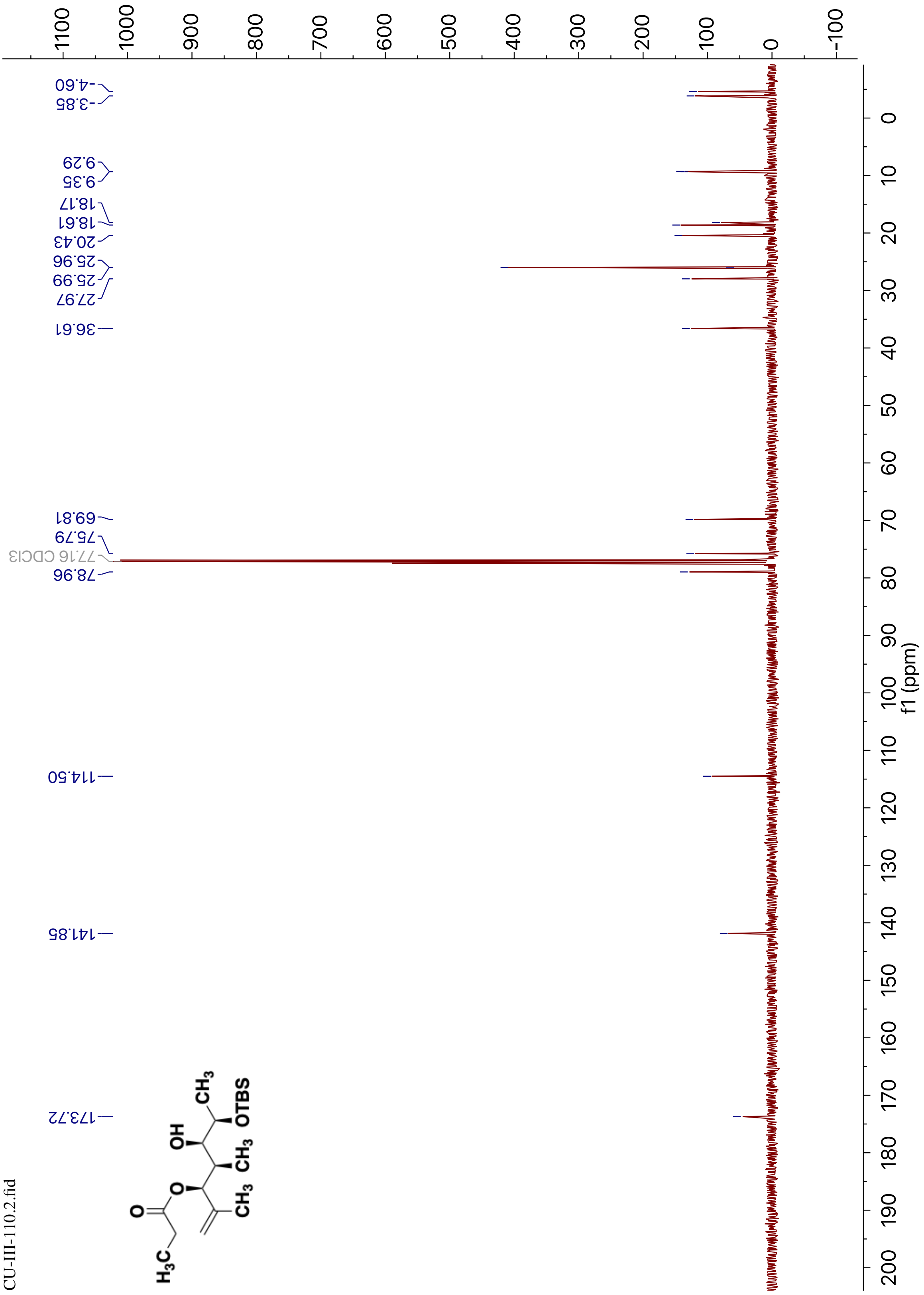
CU-III-110.2.fid
-4.60
-3.85
9.29
9.35
18.17
18.61
20.43
25.96
25.99
27.97
36.61
69.81
75.79
77.16 CDCl3
78.96
114.50
141.85
173.72
f1 (ppm)

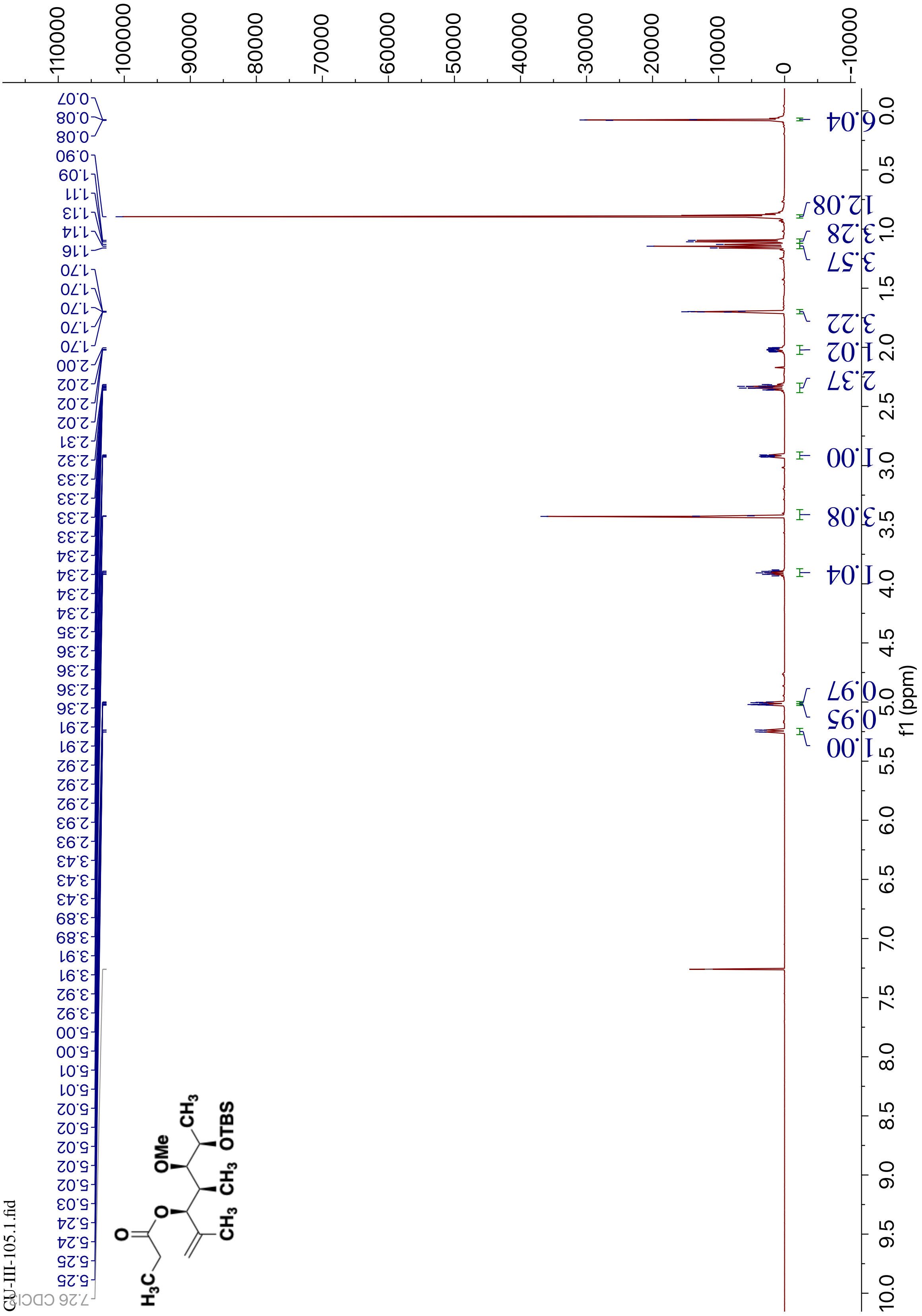
GU-III-105.1.fid
f1 (ppm)
OMe
OTBS

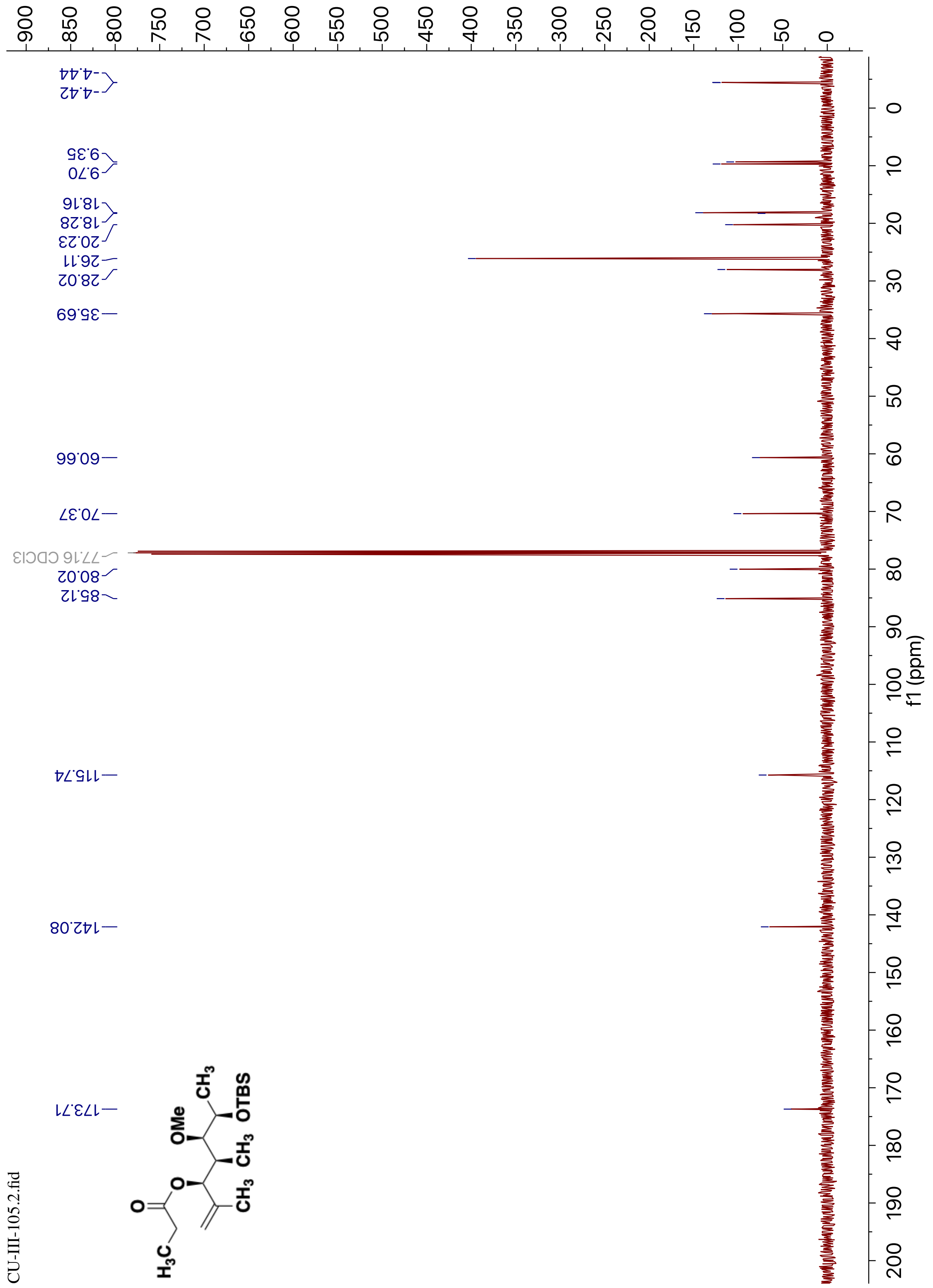
CU-III-105.2.fid
-4.44
-4.42
9.35
9.70
18.16
18.28
20.23
26.11
28.02
35.69
60.66
70.37
77.16 CDCl3
80.02
85.12
115.74
142.08
173.71
f1 (ppm)
OMe
OTBS

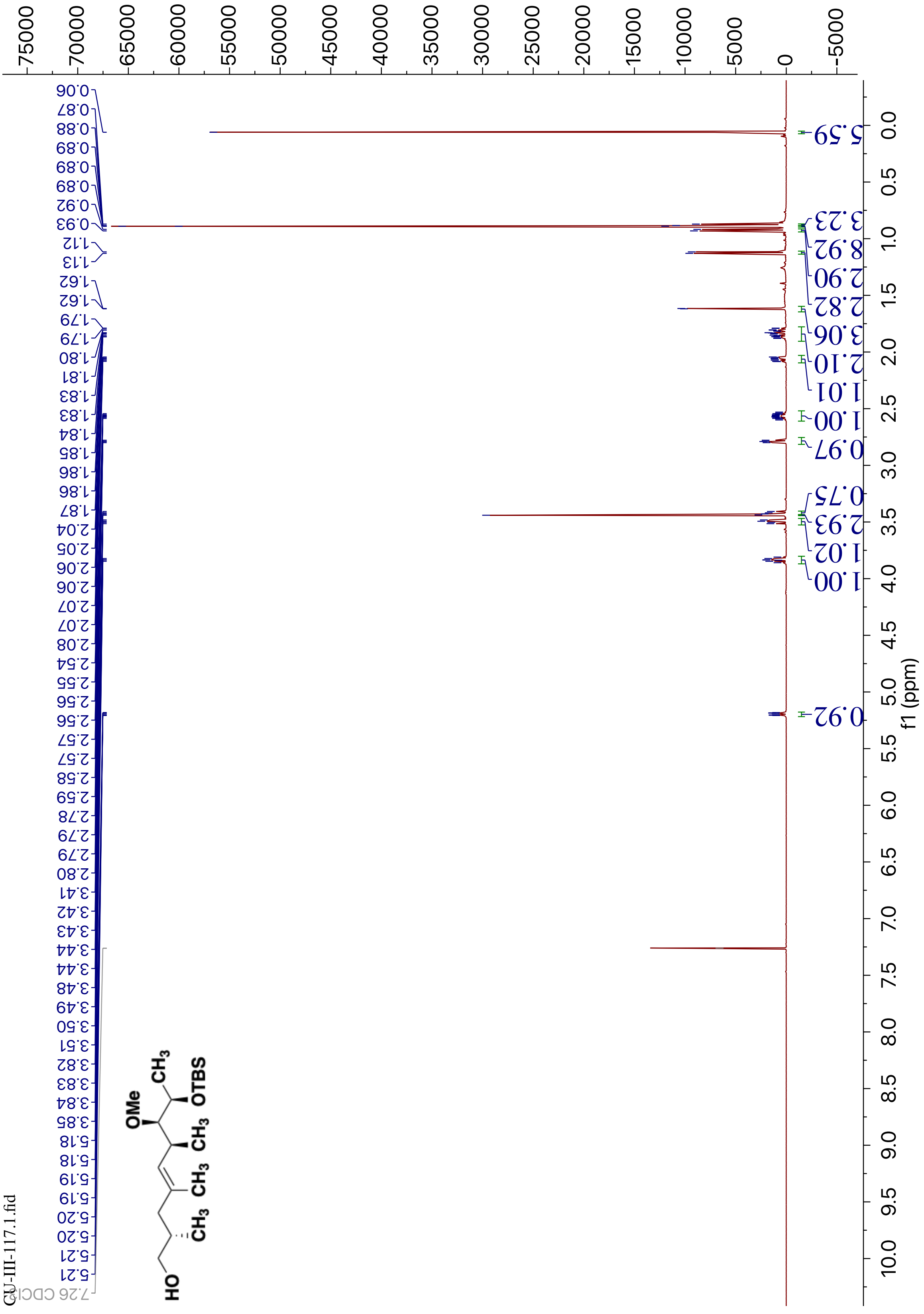
CU-III-117.1.fid
0.06
0.87
0.88
0.89
0.89
0.89
0.92
0.93
1.12
1.13
1.62
1.62
1.79
1.79
1.80
1.81
1.83
1.83
1.84
1.85
1.86
1.86
1.87
2.04
2.05
2.06
2.06
2.07
2.07
2.08
2.54
2.55
2.56
2.56
2.57
2.57
2.58
2.59
2.78
2.79
2.79
2.80
3.41
3.42
3.43
3.44
3.44
3.48
3.49
3.50
3.51
3.82
3.83
3.84
3.85
5.18
5.18
5.19
5.19
5.20
5.20
5.21
5.21
7.26 CDCl3
HO
OMe
CH3
OTBS
5.59
3.23
8.92
2.90
2.82
3.06
2.10
1.01
1.00
0.97
0.75
2.93
1.02
1.00
0.92
f1 (ppm)

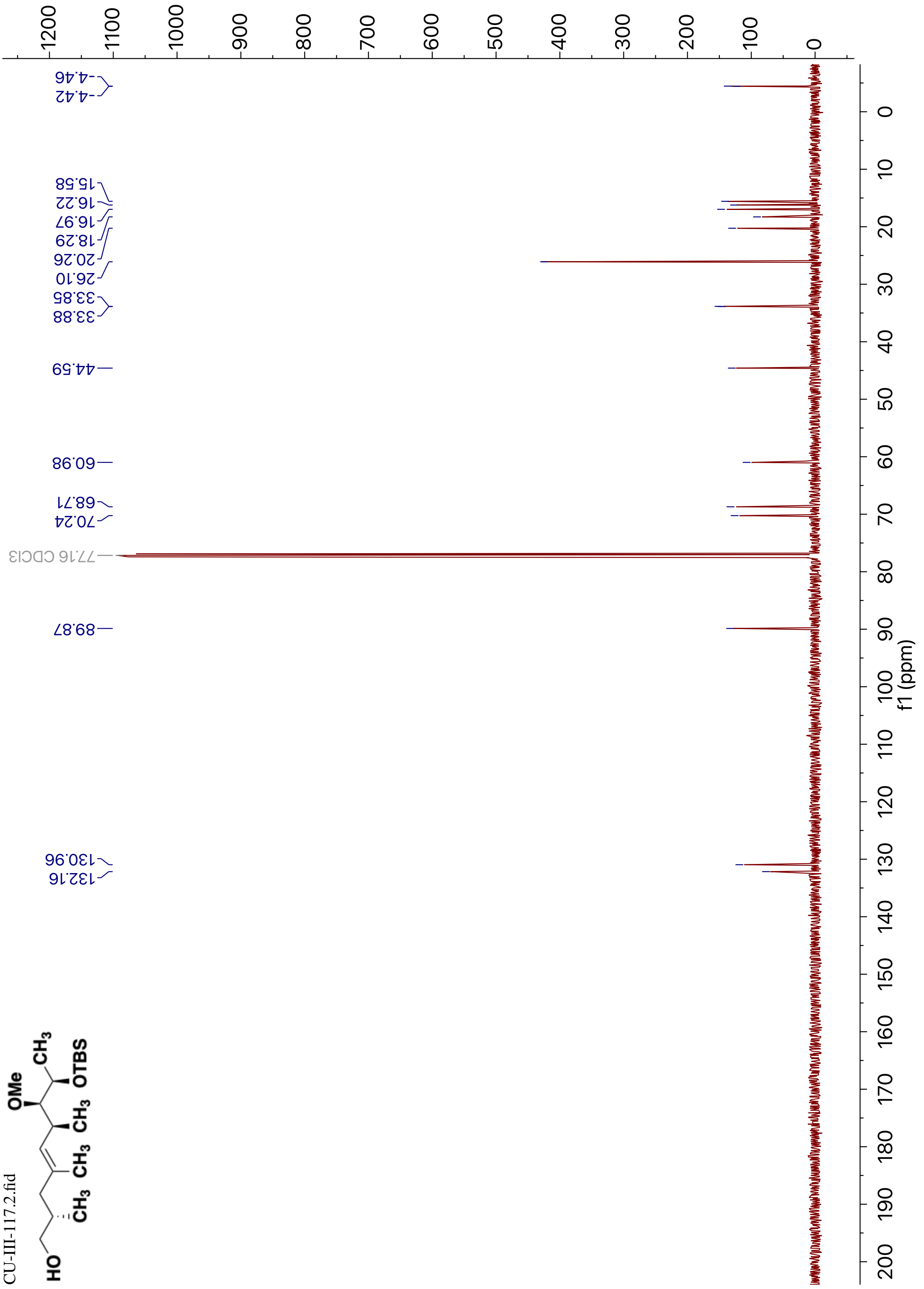
CU-III-117.2.fid
-4.46
-4.42
15.58
16.22
16.97
18.29
20.26
26.10
33.85
33.88
44.59
60.98
68.71
70.24
77.16 CDCl3
89.87
130.96
132.16
f1 (ppm)
HO
OMe
OTBS

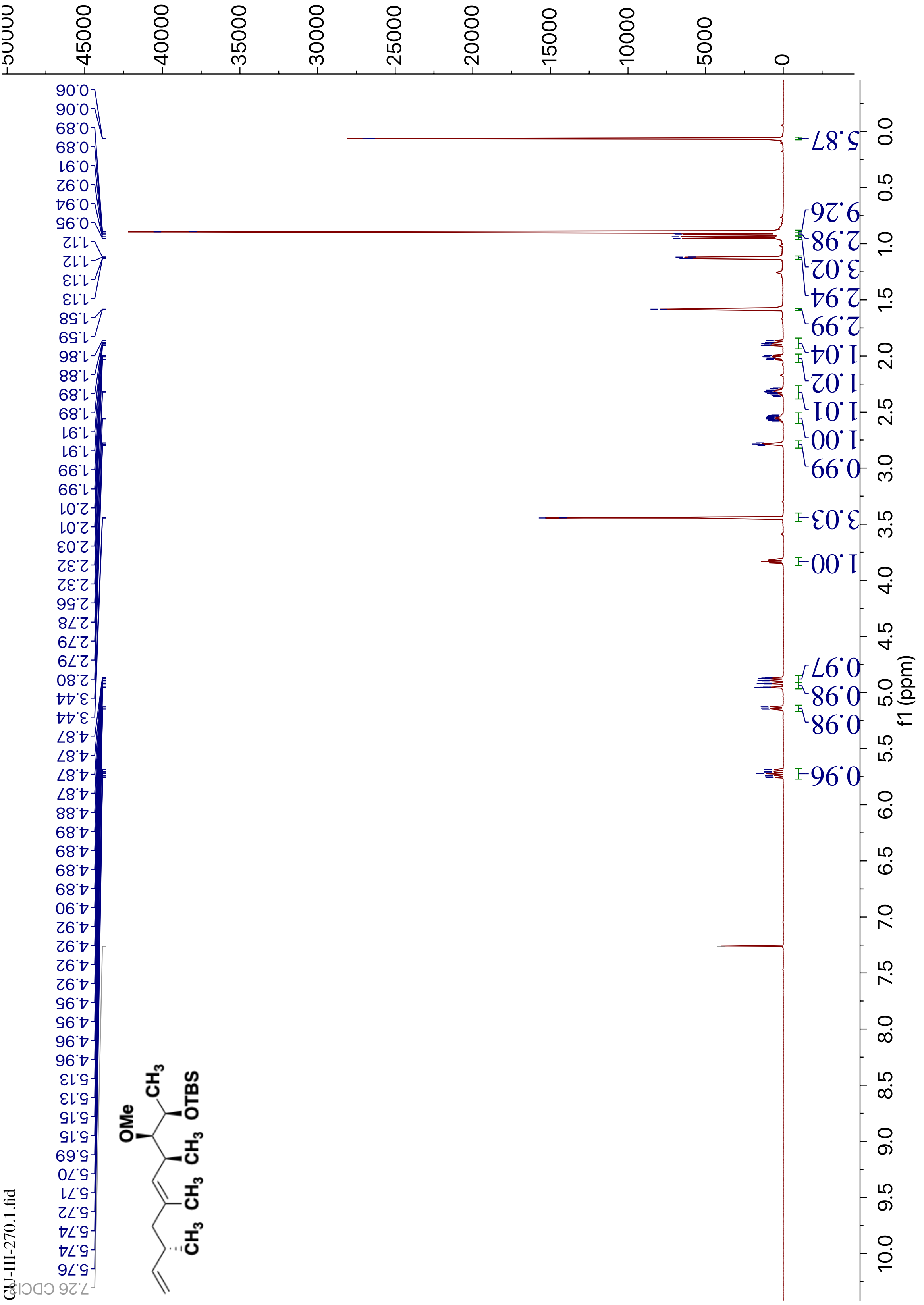
f1 (ppm)

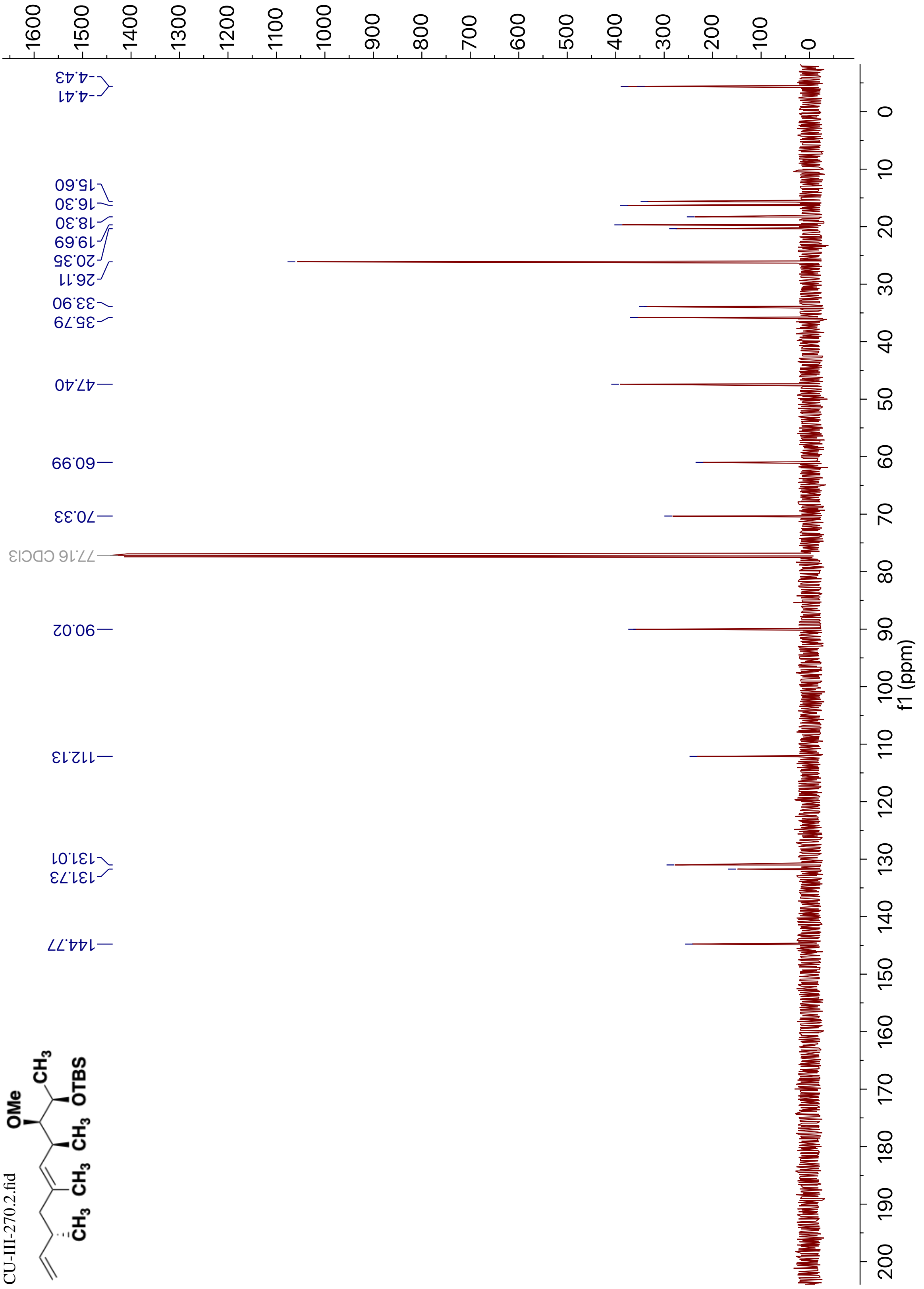
CU-III-270.2.fid
OMe
CH3
OTBS
-4.43
-4.41
15.60
16.30
18.30
19.69
20.35
26.11
33.90
35.79
47.40
60.99
70.33
77.16 CDCl3
90.02
112.13
131.01
131.73
144.77
f1 (ppm)

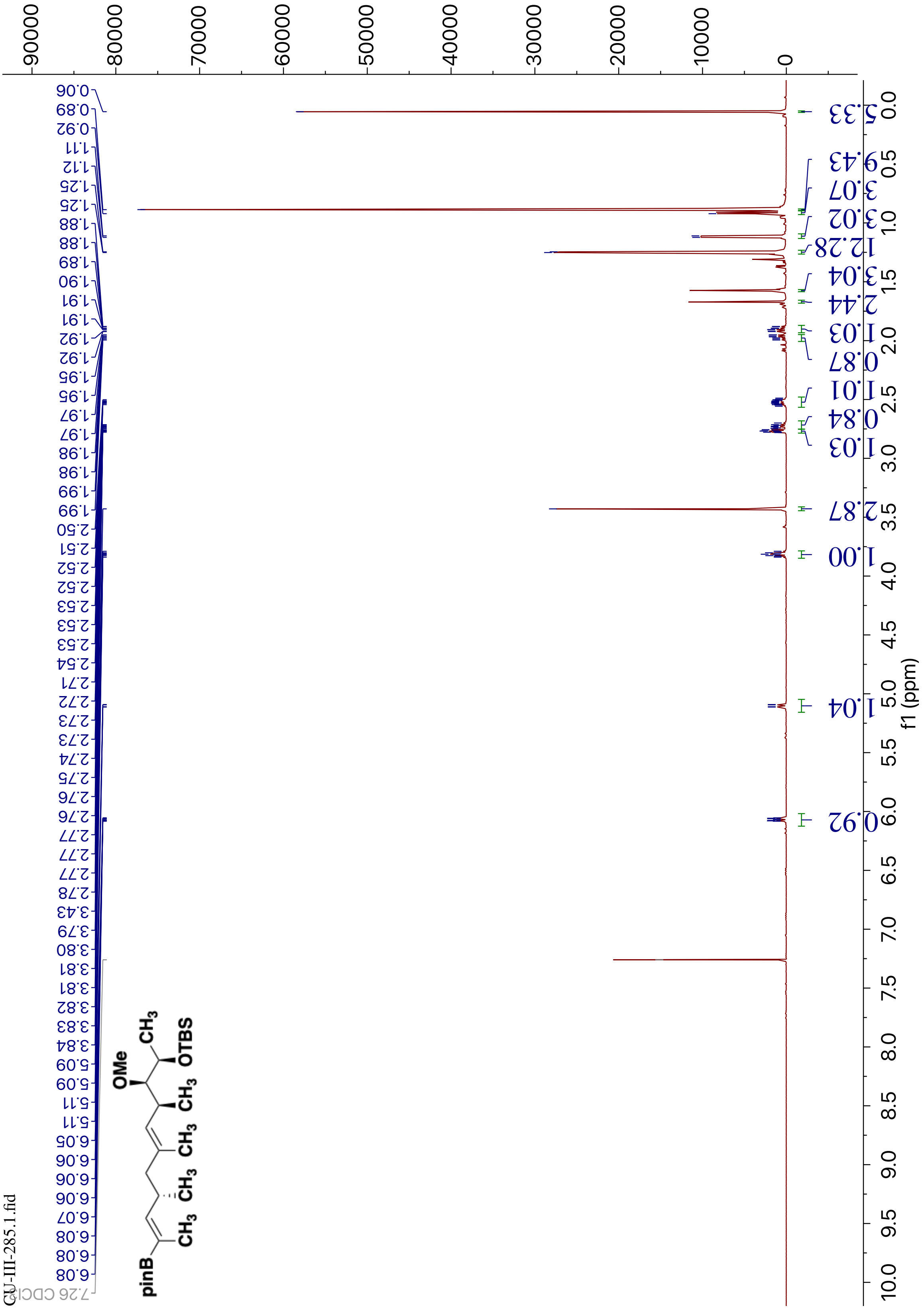
CU-III-285.1.fid
7.26 CDCl3
f1 (ppm)
pinB
OMe
OTBS

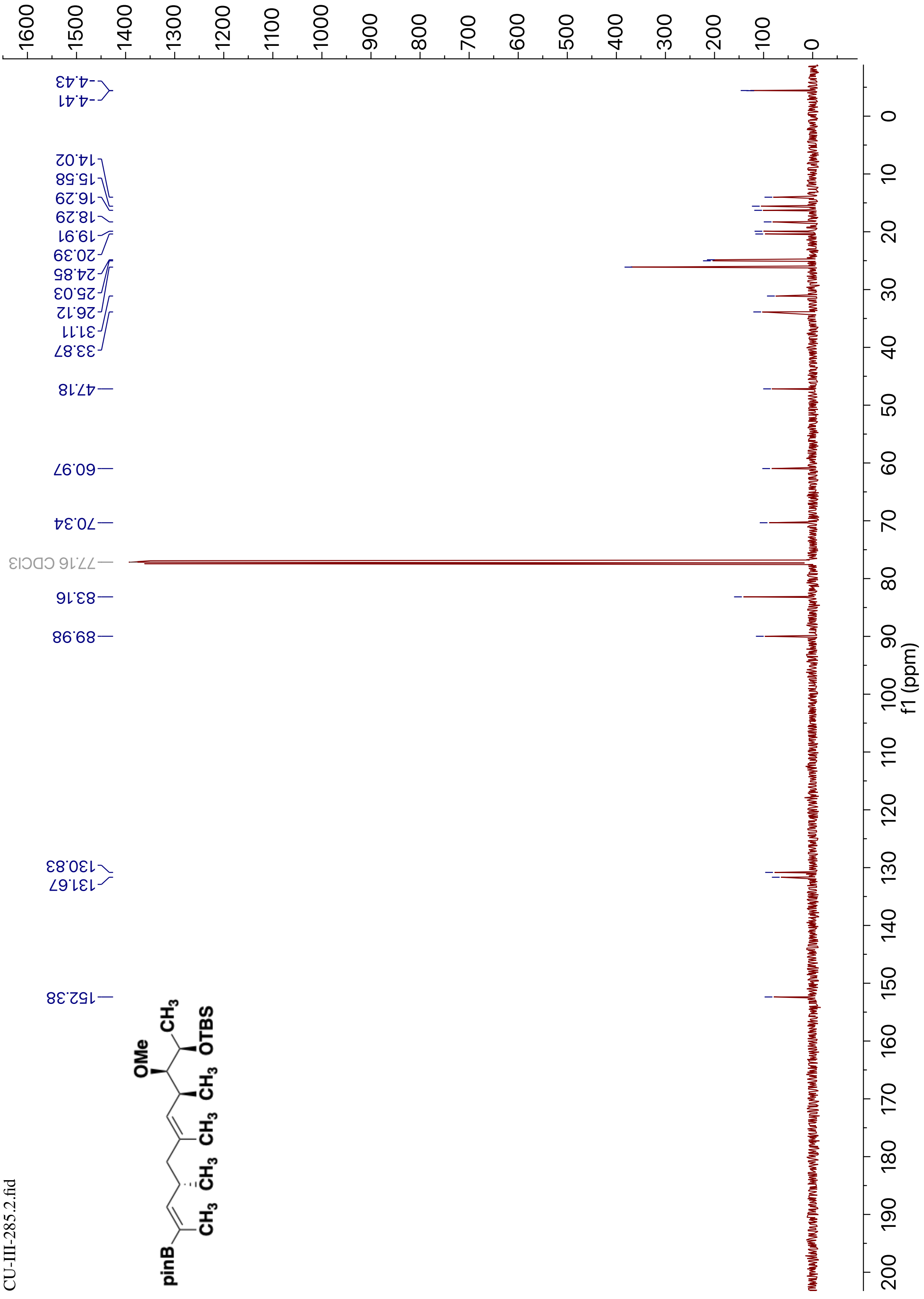
CU-III-285.2.fid
-4.43
-4.41
14.02
15.58
16.29
18.29
19.91
20.39
24.85
25.03
26.12
31.11
33.87
47.18
60.97
70.34
77.16 CDCl3
83.16
89.98
130.83
131.67
152.38
pinB
OMe
OTBS
f1 (ppm)

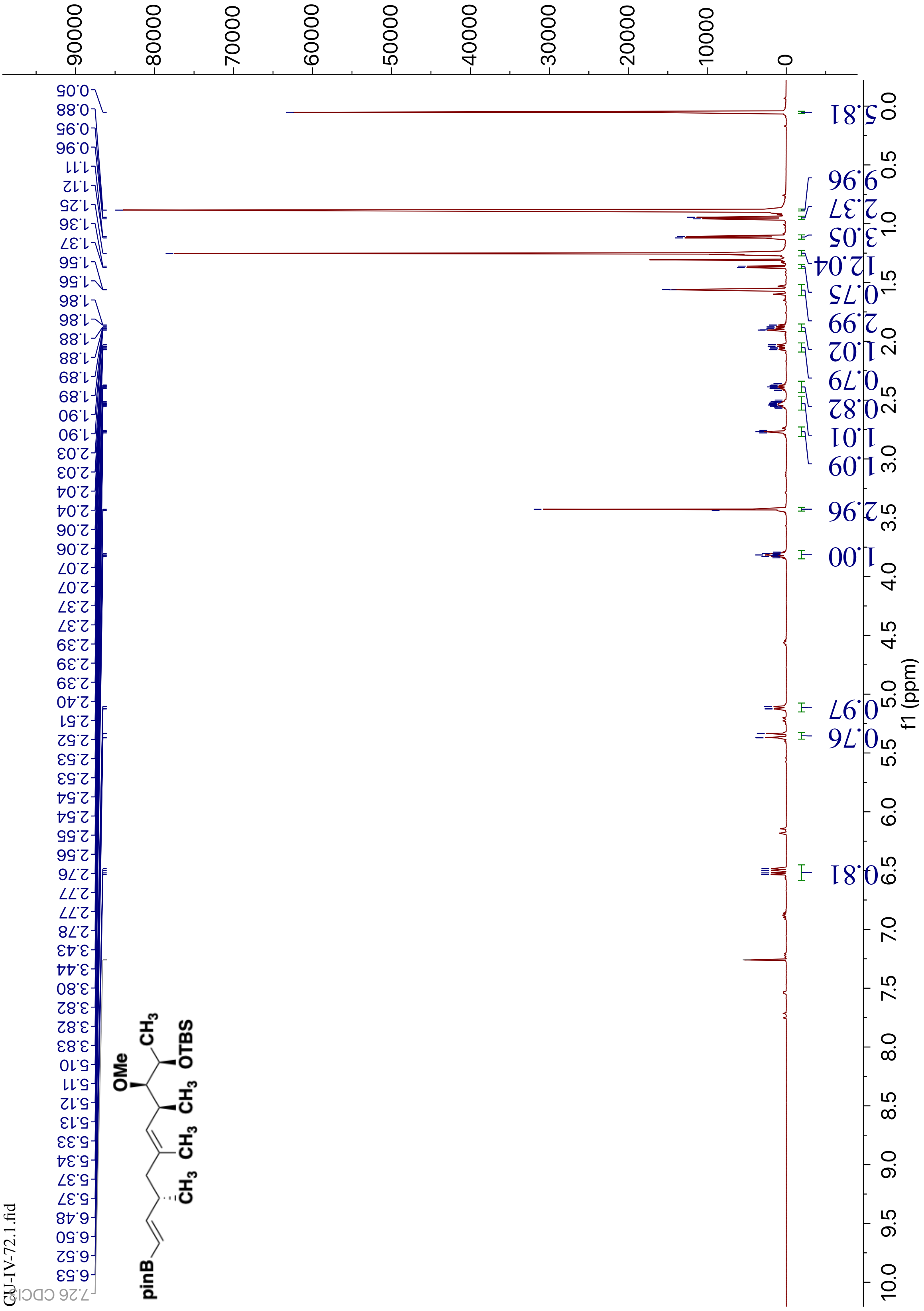
CU-IV-72.1.fid
0.05
0.88
0.95
0.96
1.11
1.12
1.25
1.36
1.37
1.56
1.56
1.86
1.86
1.88
1.88
1.89
1.89
1.90
1.90
2.03
2.03
2.04
2.04
2.06
2.06
2.07
2.07
2.37
2.37
2.39
2.39
2.39
2.40
2.51
2.52
2.53
2.53
2.54
2.54
2.55
2.56
2.76
2.77
2.77
2.78
3.43
3.44
3.80
3.82
3.82
3.83
5.10
5.11
5.12
5.13
5.33
5.34
5.37
5.37
6.48
6.50
6.52
6.53
7.26 CDCl3
pinB
CH3
CH3
CH3
OMe
CH3
OTBS
5.81
9.96
2.37
3.05
12.04
0.75
2.99
1.02
0.79
0.82
1.01
1.09
2.96
1.00
0.97
0.76
0.81
f1 (ppm)

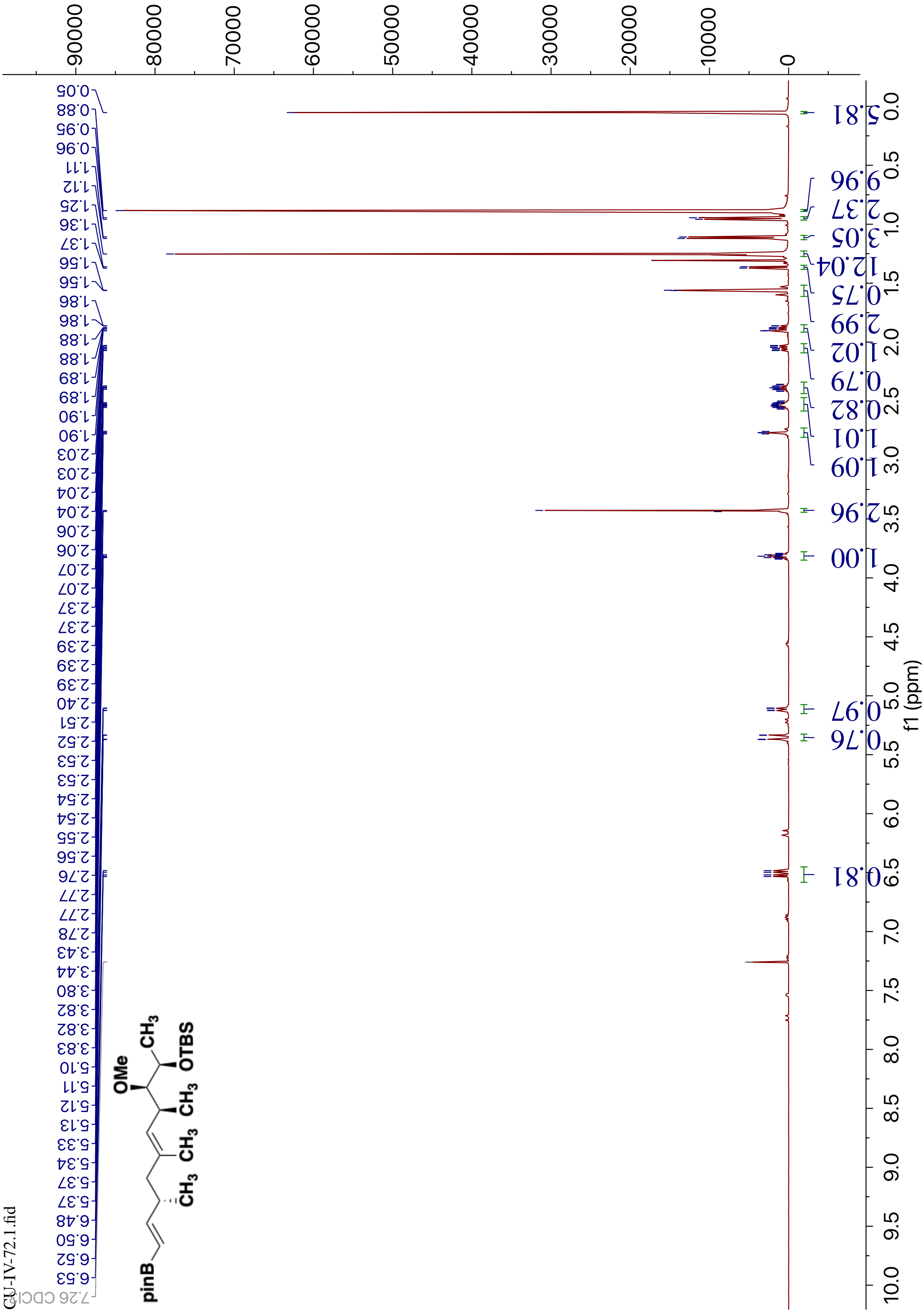
CU-IV-72.1.fid
7.26 CDCl3
f1 (ppm)
pinB
OMe
OTBS

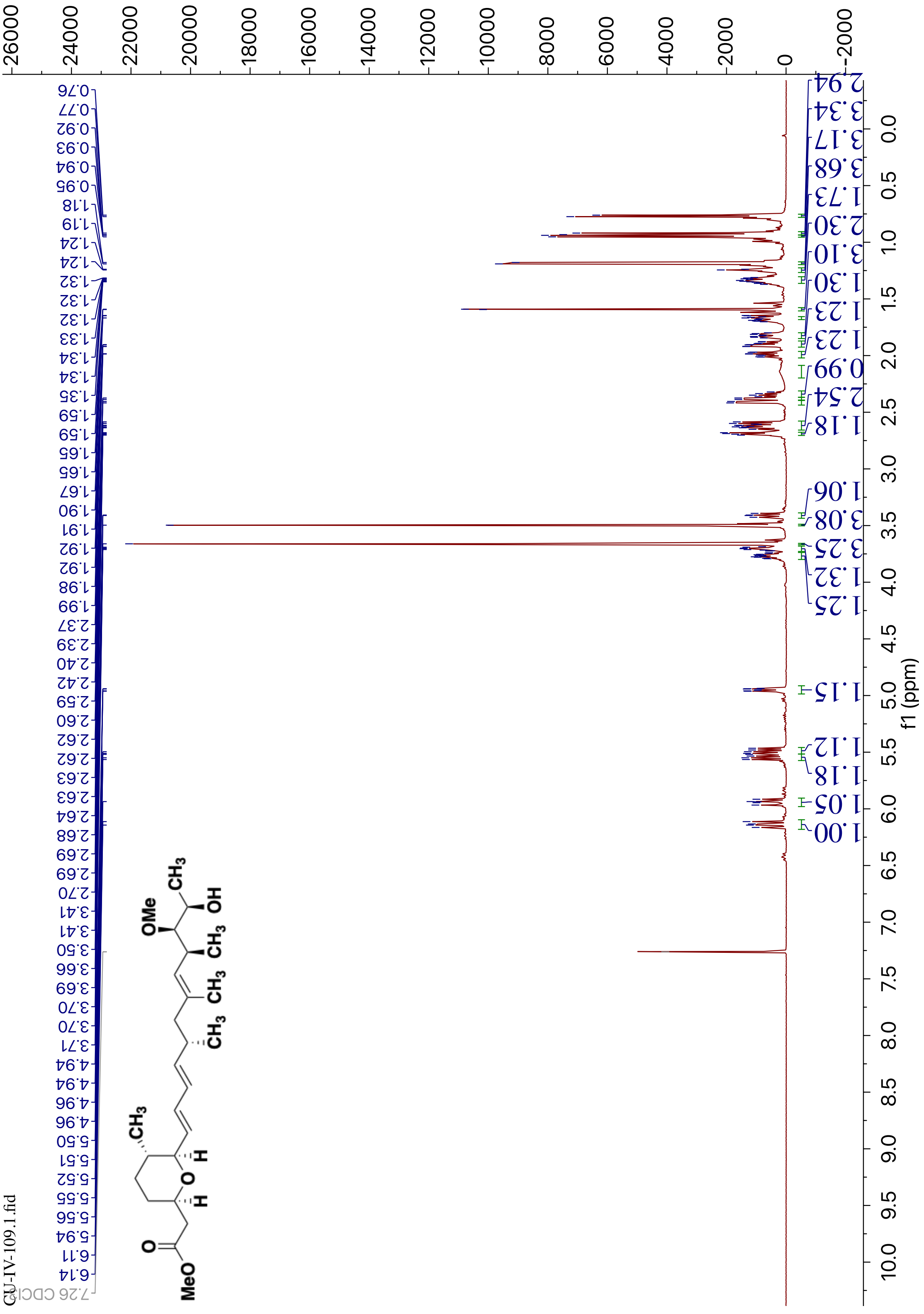
GU-IV-109.1.fid
0.76 0.77 0.92 0.93 0.94 0.95 1.18 1.19 1.24 1.24 1.32 1.32 1.32 1.33 1.34 1.34 1.35 1.59 1.59 1.65 1.65 1.67 1.90 1.91 1.92 1.92 1.98 1.99 2.37 2.39 2.40 2.42 2.59 2.60 2.62 2.62 2.63 2.63 2.64 2.68 2.69 2.69 2.70 3.41 3.41 3.50 3.66 3.69 3.70 3.70 3.71 4.94 4.94 4.96 4.96 5.50 5.51 5.52 5.55 5.56 5.94 6.11 6.14 7.26 CDCl3
2.94 3.34 3.17 3.68 1.73 2.30 3.10 1.30 1.23 1.23 0.99 2.54 1.18 1.06 3.08 3.25 1.32 1.25 1.15 1.12 1.18 1.05 1.00
f1 (ppm)
MeO
O
H
CH3
OMe
OH

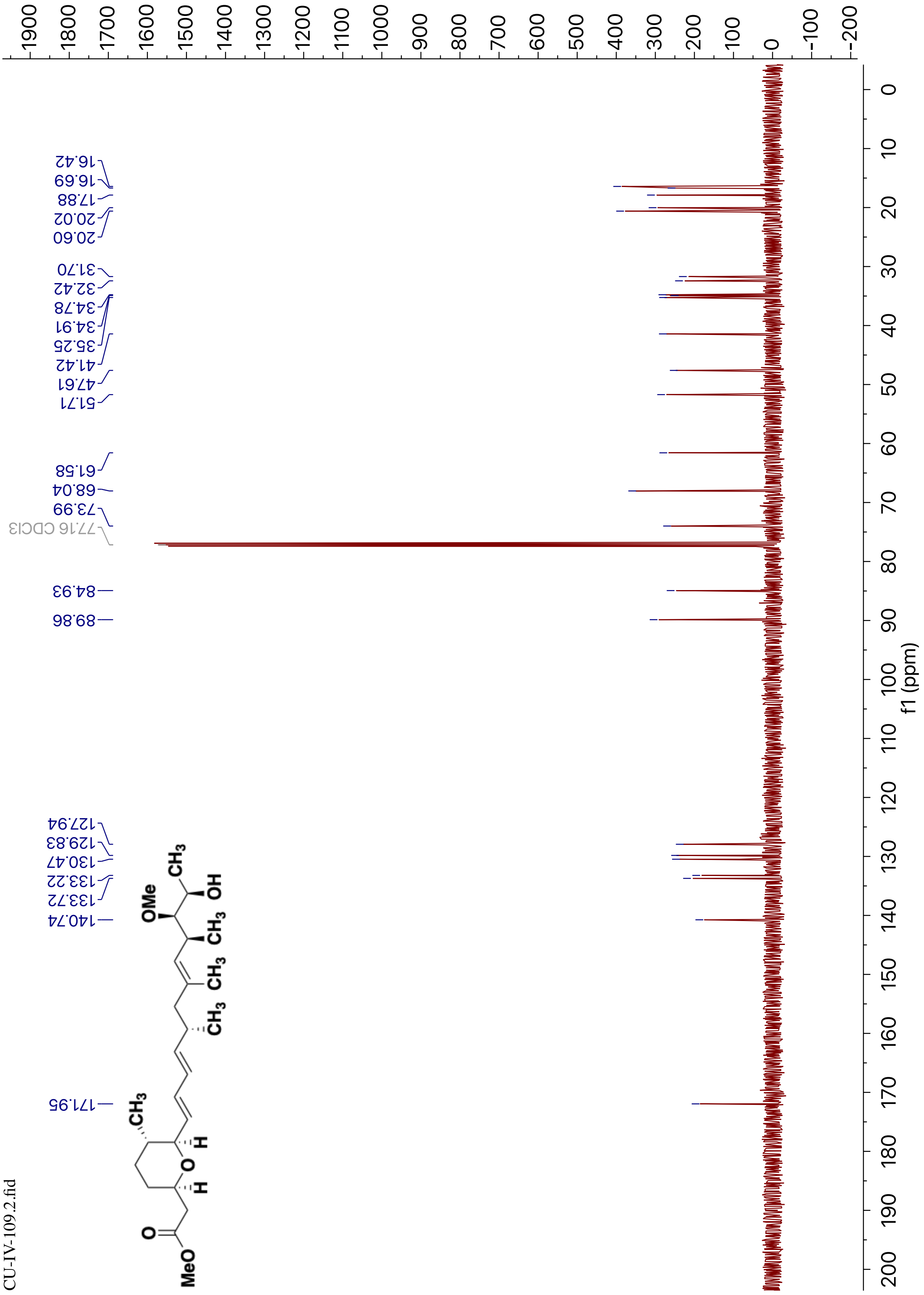
CU-IV-109.2.fid
171.95
140.74
133.72
133.22
130.47
129.83
127.94
89.86
84.93
77.16 CDCl3
73.99
68.04
61.58
51.71
47.61
41.42
35.25
34.91
34.78
32.42
31.70
20.60
20.02
17.88
16.69
16.42
f1 (ppm)

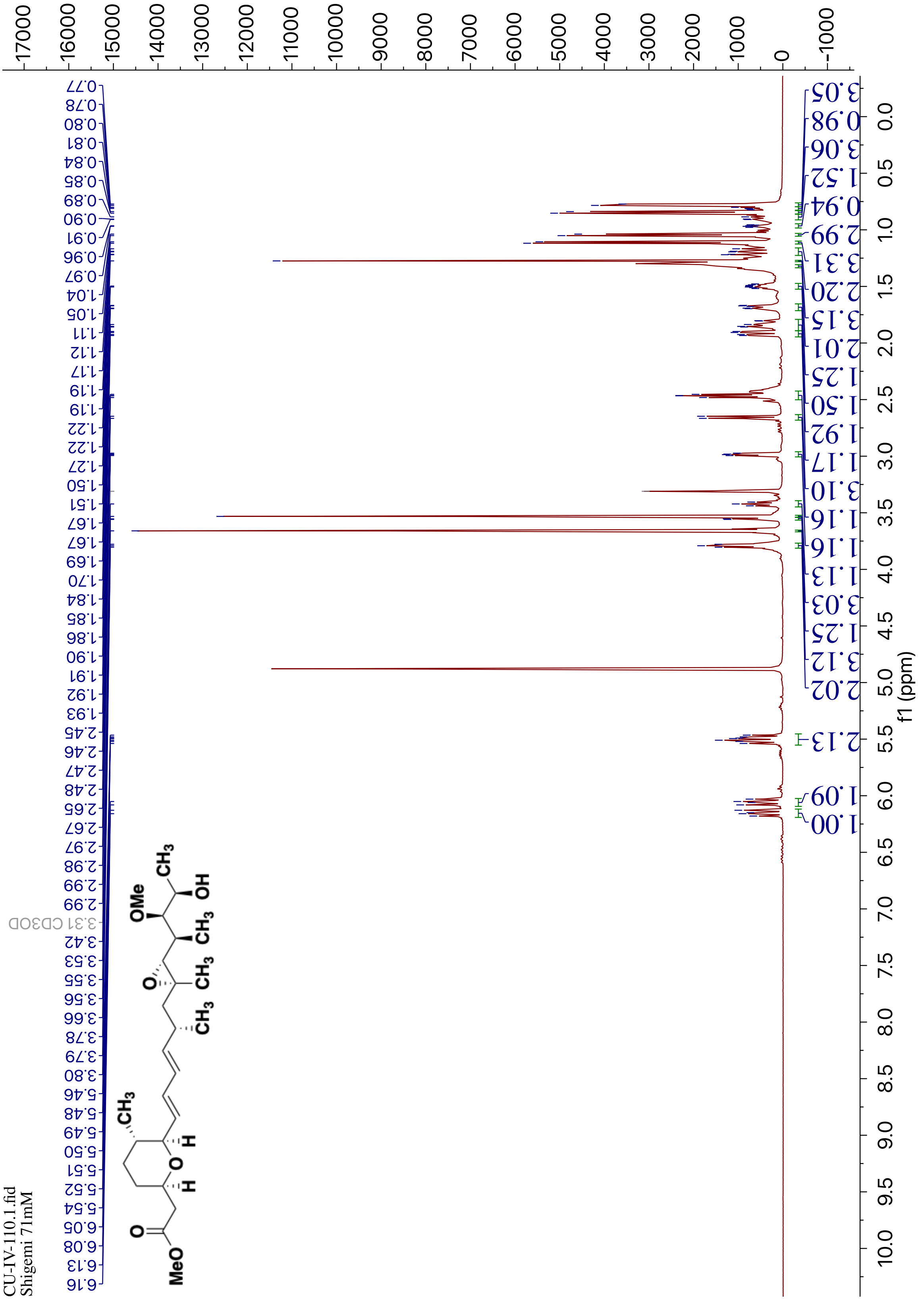
CU-IV-110.1.fid
Shigemi 71mM
3.31 CD3OD
f1 (ppm)
MeO
OMe
OH

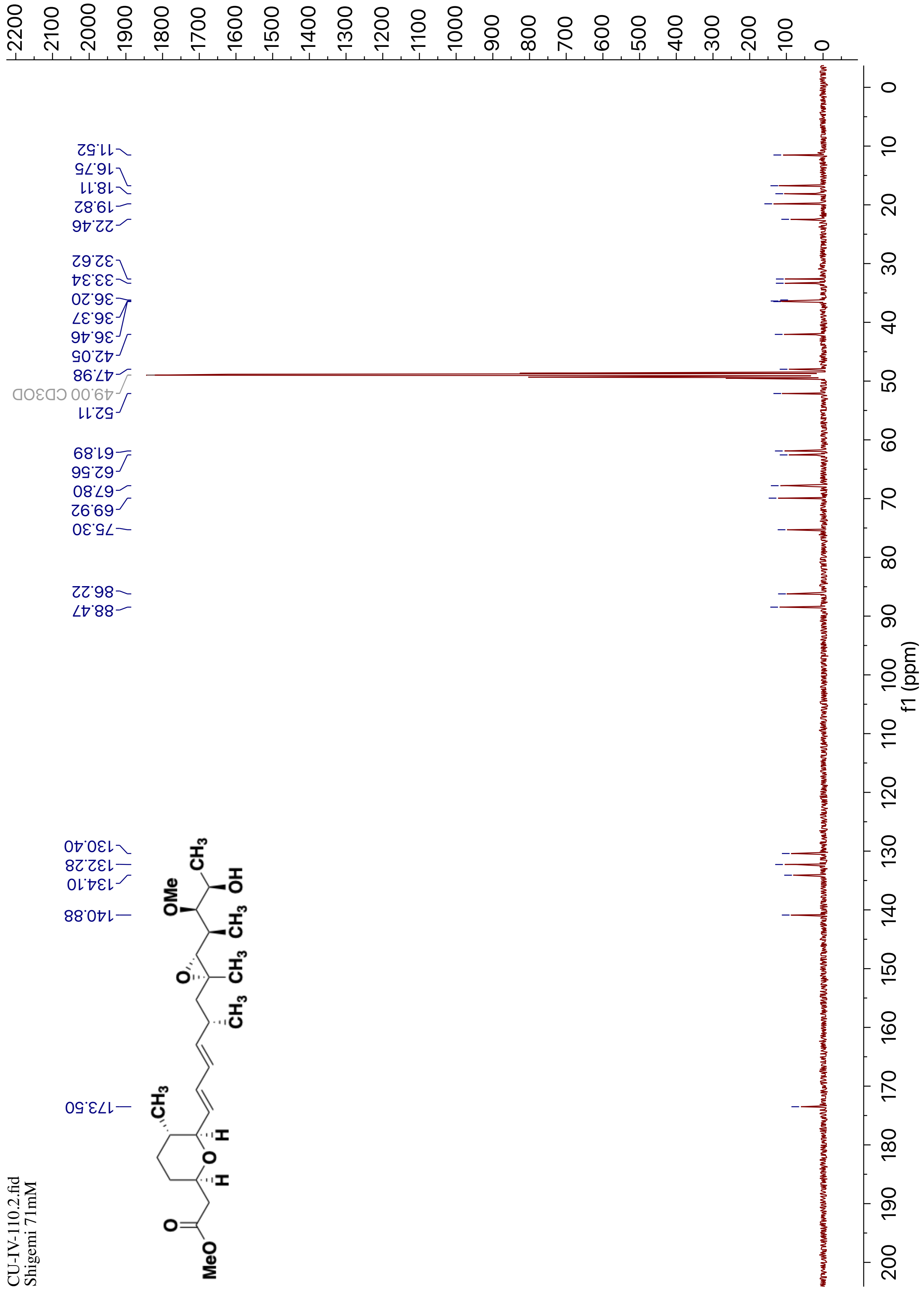
CU-IV-110.2.fid
Shigemi 71mM
11.52
16.75
18.11
19.82
22.46
32.62
33.34
36.20
36.37
36.46
42.05
47.98
49.00 CD3OD
52.11
61.89
62.56
67.80
69.92
75.30
86.22
88.47
130.40
132.28
134.10
140.88
173.50
MeO
O
H
H
CH3
OMe
CH3
OH
f1 (ppm)

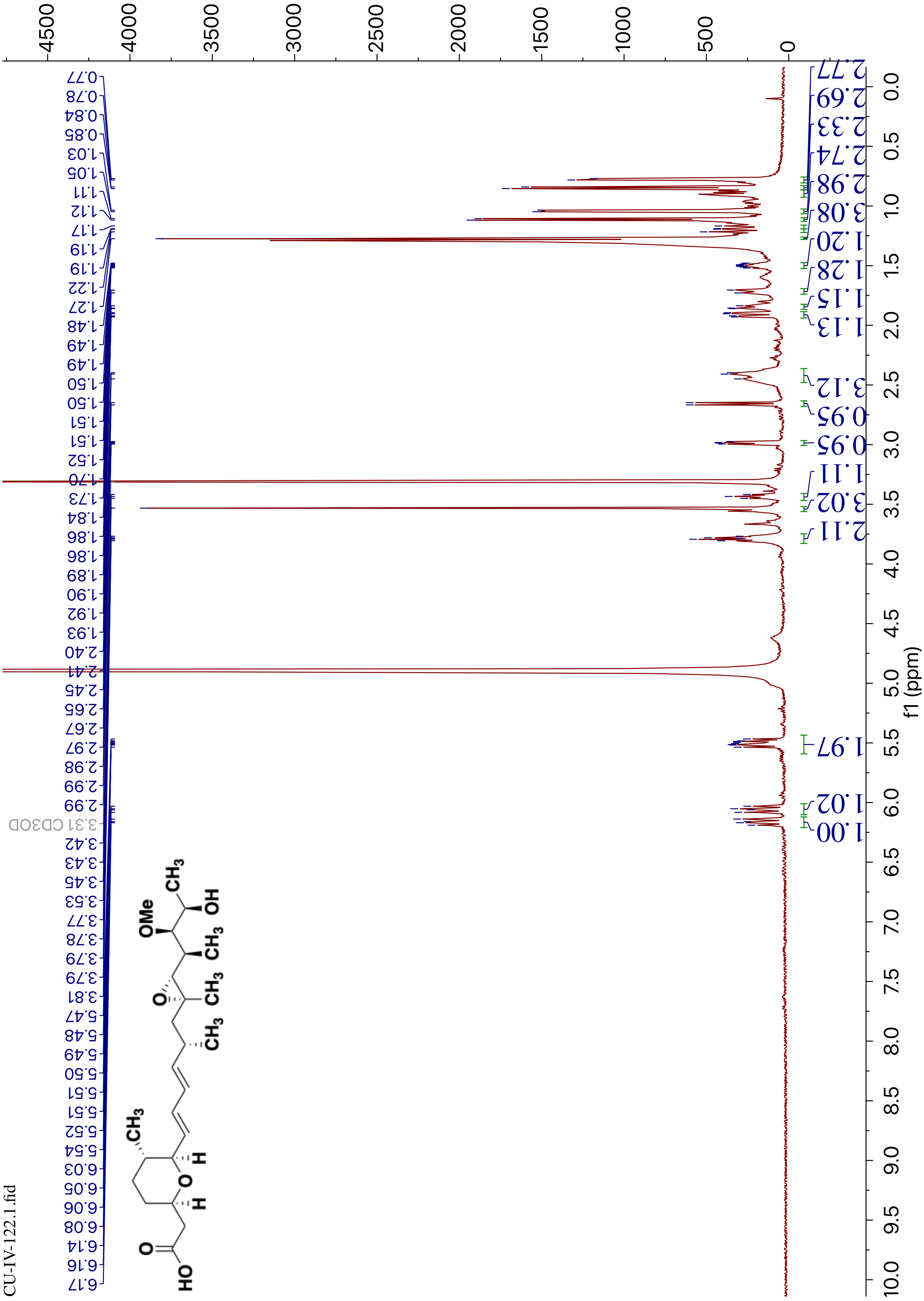
CU-IV-122.1.fid
f1 (ppm)
3.31 CD3OD

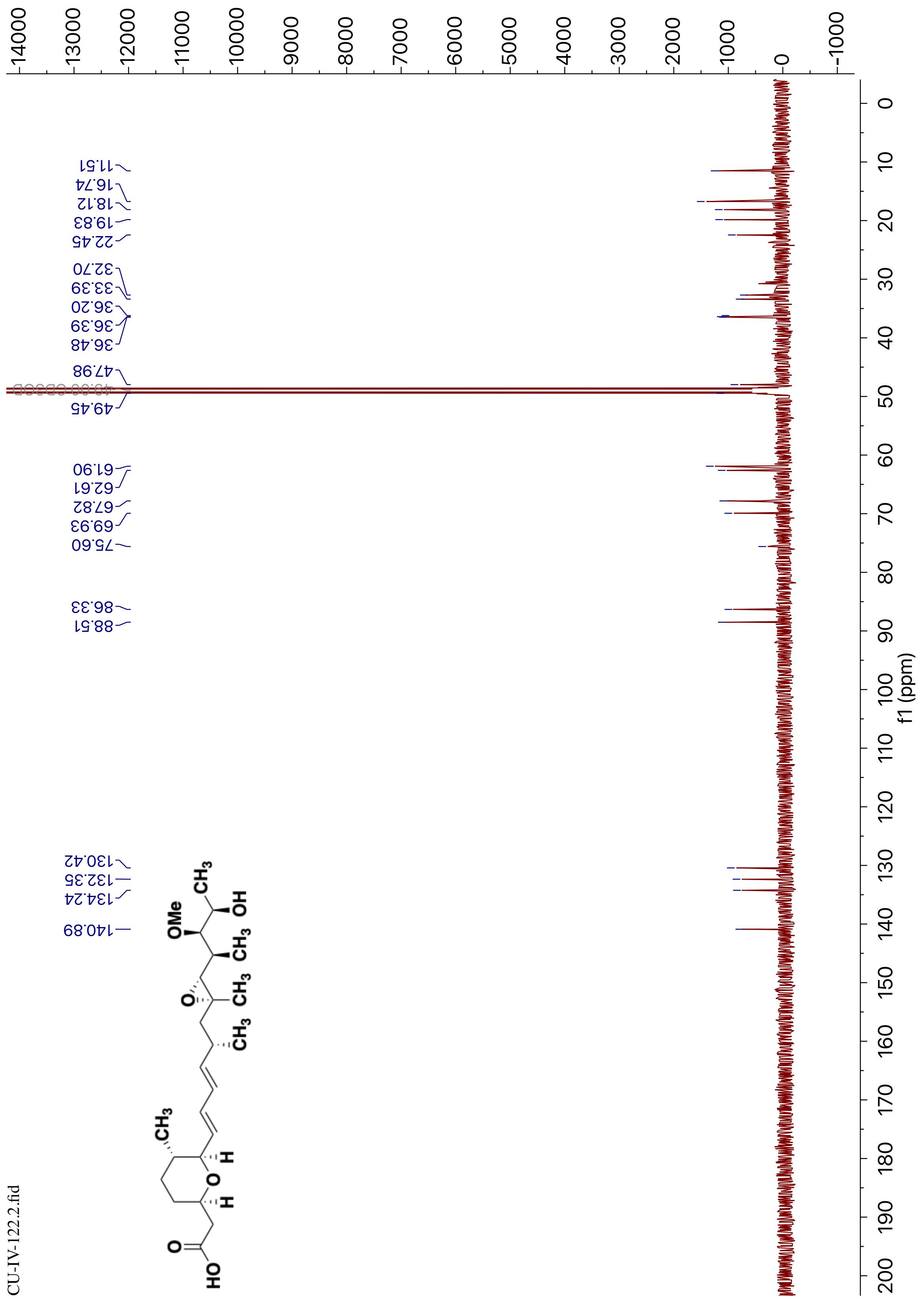
CU-IV-122.2.fid
11.51
16.74
18.12
19.83
22.45
32.70
33.39
36.20
36.39
36.48
47.98
49.45
61.90
62.61
67.82
69.93
75.60
86.33
88.51
130.42
132.35
134.24
140.89
f1 (ppm)

Mass Spectrometry & Proteomics Facility

Mass Spectrum SmartFormula Report

Analysis Info

Analysis Name	D:\Data\0516\CU-IV-122.d	Acquisition Date	5/16/2022 11:36:59 AM
Method	0511 tune low.m	Operator	BDAL@DE
Sample Name	CU-IV-122	Instrument / Ser#	micrOTOF II 8213750.10314
Comment	direct infusion		

Acquisition Parameter

Source Type	ESI	Ion Polarity	Positive	Set Nebulizer	0.4 Bar
Focus	Not active	Set Capillary	4500 V	Set Dry Heater	180 °C
Scan Begin	50 m/z	Set End Plate Offset	-500 V	Set Dry Gas	4.0 l/min
Scan End	1650 m/z	n/a	n/a	Set Divert Valve	Waste

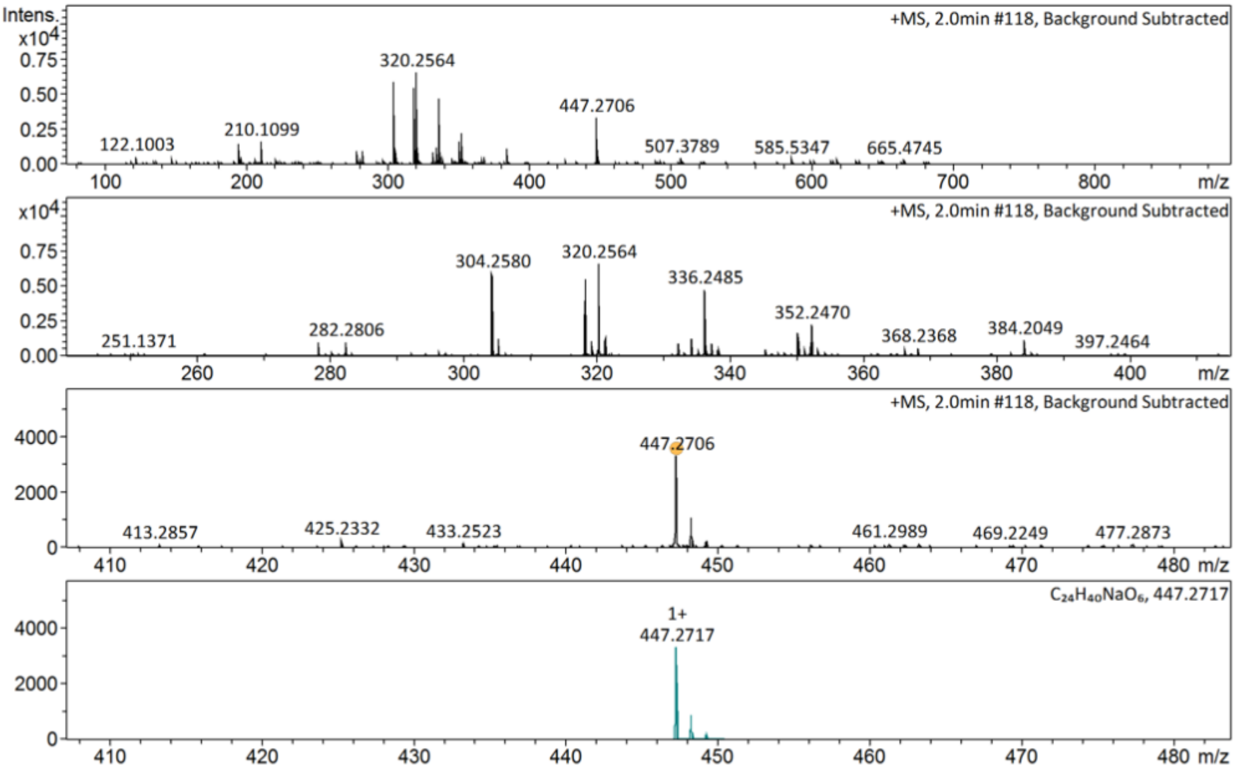

Meas. m/z	#	Ion Formula	m/z	err [ppm]	Mean err [ppm]	rdb	N-Rule	e⁻ Conf
447.270631	1	C24H40NaO6	447.271710	2.4	4.2	4.5	ok	even

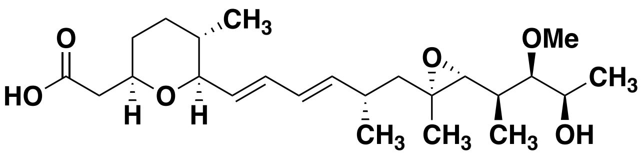

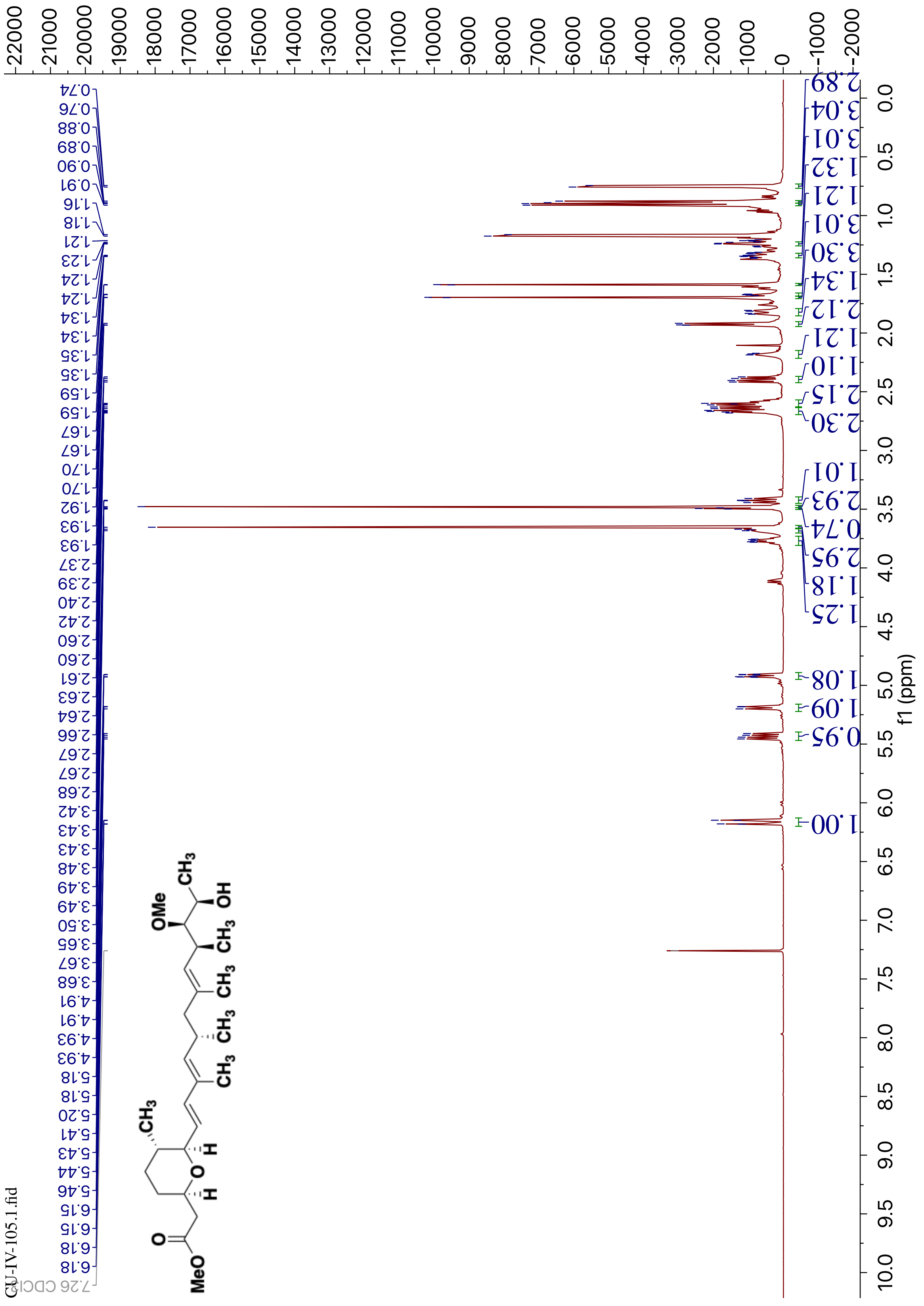
GU-IV-105.1.fid
7.26 CDCl3
f1 (ppm)
MeO
OMe
OH

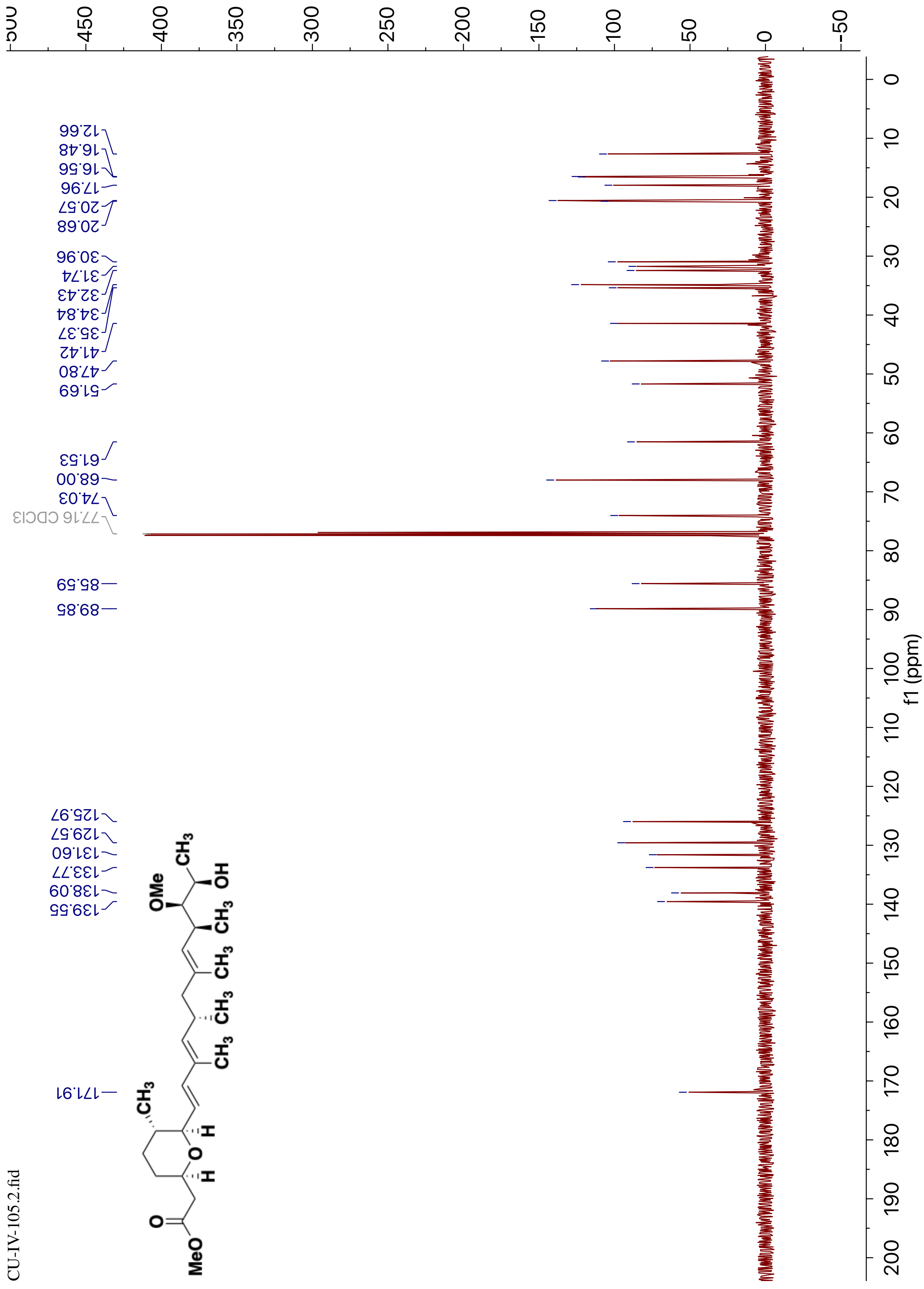
CU-IV-105.2.fid
12.66
16.48
16.56
17.96
20.57
20.68
30.96
31.74
32.43
34.84
35.37
41.42
47.80
51.69
61.53
68.00
74.03
77.16 CDCl3
85.59
89.85
125.97
129.57
131.60
133.77
138.09
139.55
171.91
f1 (ppm)
MeO
O
H
CH3
OMe
OH

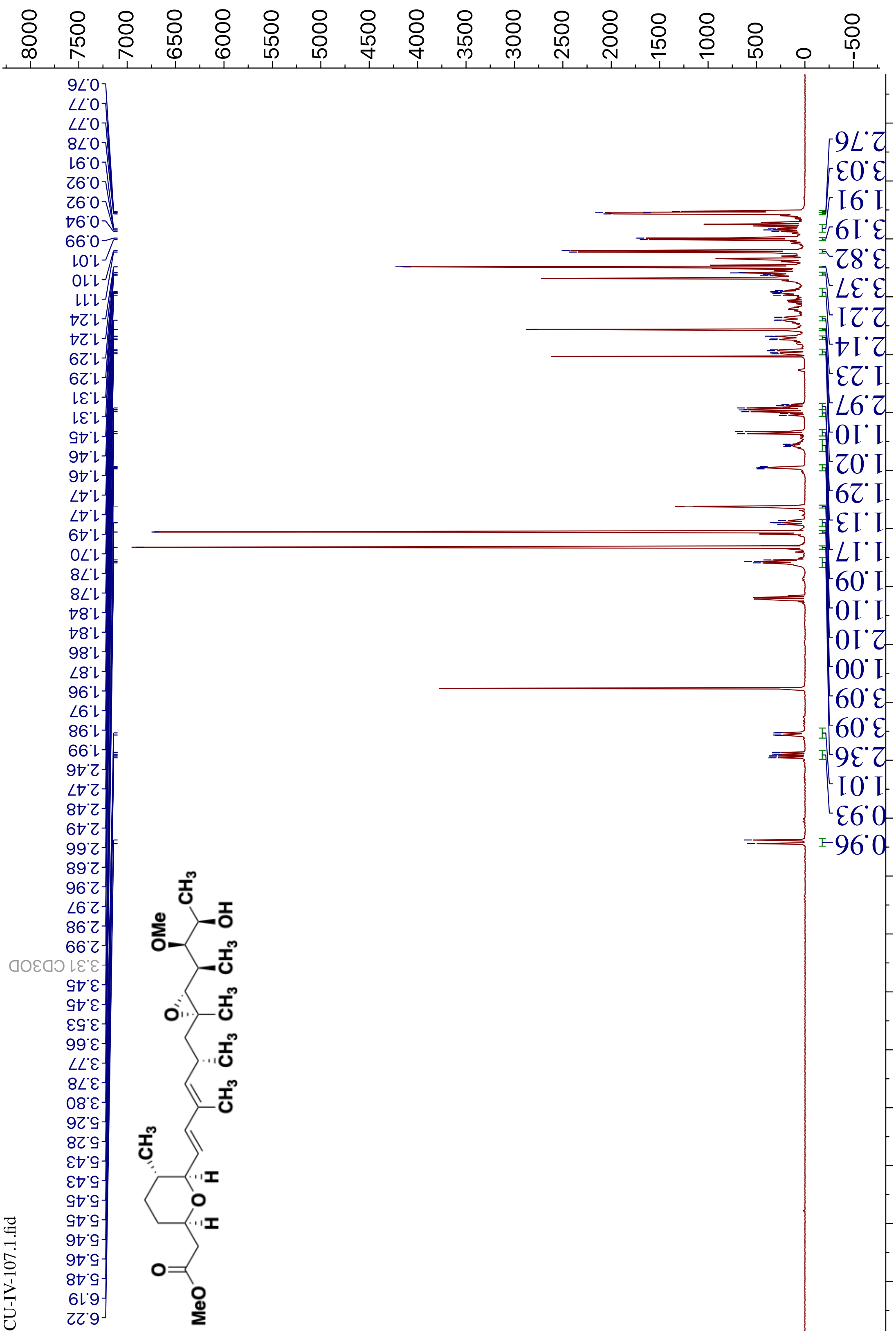
CU-IV-107.1.fid
0.76
0.77
0.77
0.78
0.91
0.92
0.92
0.94
0.99
1.01
1.10
1.11
1.24
1.24
1.29
1.29
1.31
1.31
1.45
1.46
1.46
1.47
1.47
1.49
1.70
1.78
1.78
1.84
1.84
1.86
1.87
1.96
1.97
1.98
1.99
2.46
2.47
2.48
2.49
2.66
2.68
2.96
2.97
2.98
2.99
3.31 CD3OD
3.45
3.45
3.53
3.66
3.77
3.78
3.80
5.26
5.28
5.43
5.43
5.45
5.45
5.46
5.46
5.48
6.19
6.22
MeO
O
H
O
H
CH3
CH3
CH3
O
CH3
CH3
OMe
OH
CH3
2.76
3.03
1.91
3.19
3.82
3.37
2.21
2.14
1.23
2.97
1.10
1.02
1.29
1.13
1.17
1.09
1.10
2.10
1.00
3.09
3.09
2.36
1.01
0.93
0.96
f1 (ppm)

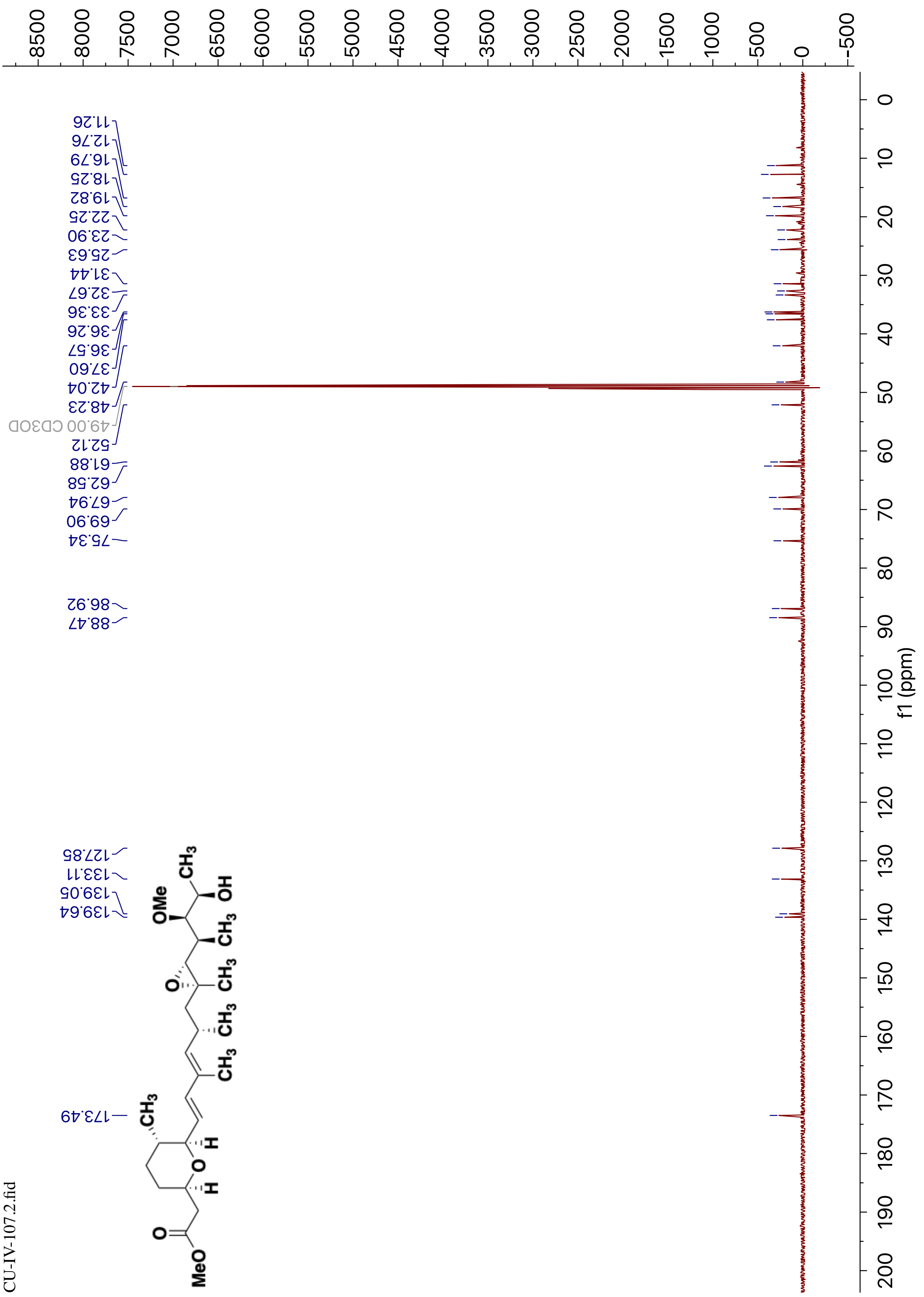
CU-IV-107.2.fid
11.26
12.76
16.79
18.25
19.82
22.25
23.90
25.63
31.44
32.67
33.36
36.26
36.57
37.60
42.04
48.23
49.00 CD3OD
52.12
61.88
62.58
67.94
69.90
75.34
86.92
88.47
127.85
133.11
139.05
139.64
173.49
MeO
O
H
CH3
OMe
OH
f1 (ppm)

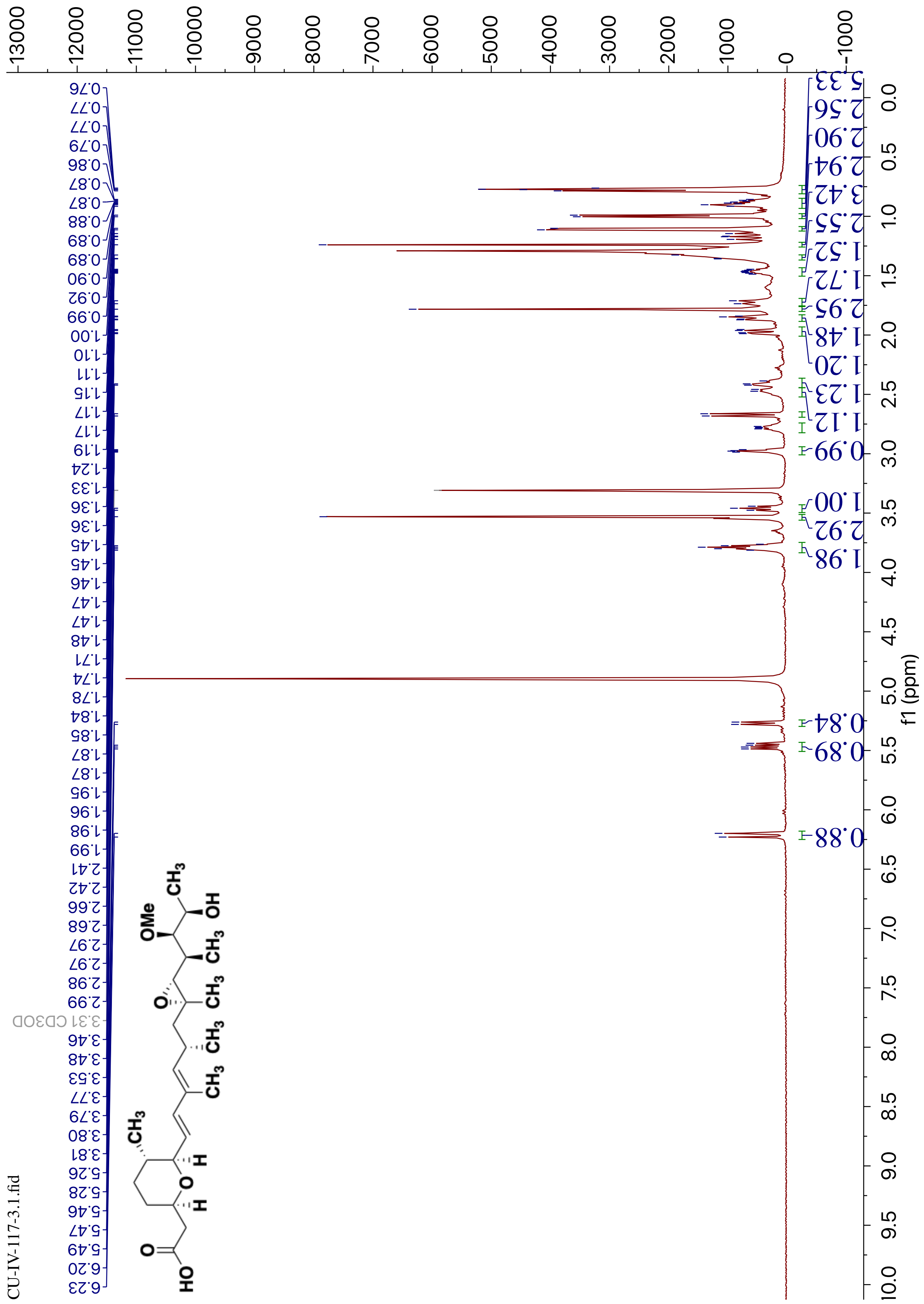
CU-IV-117-3.1.fid
3.31 CD3OD
f1 (ppm)

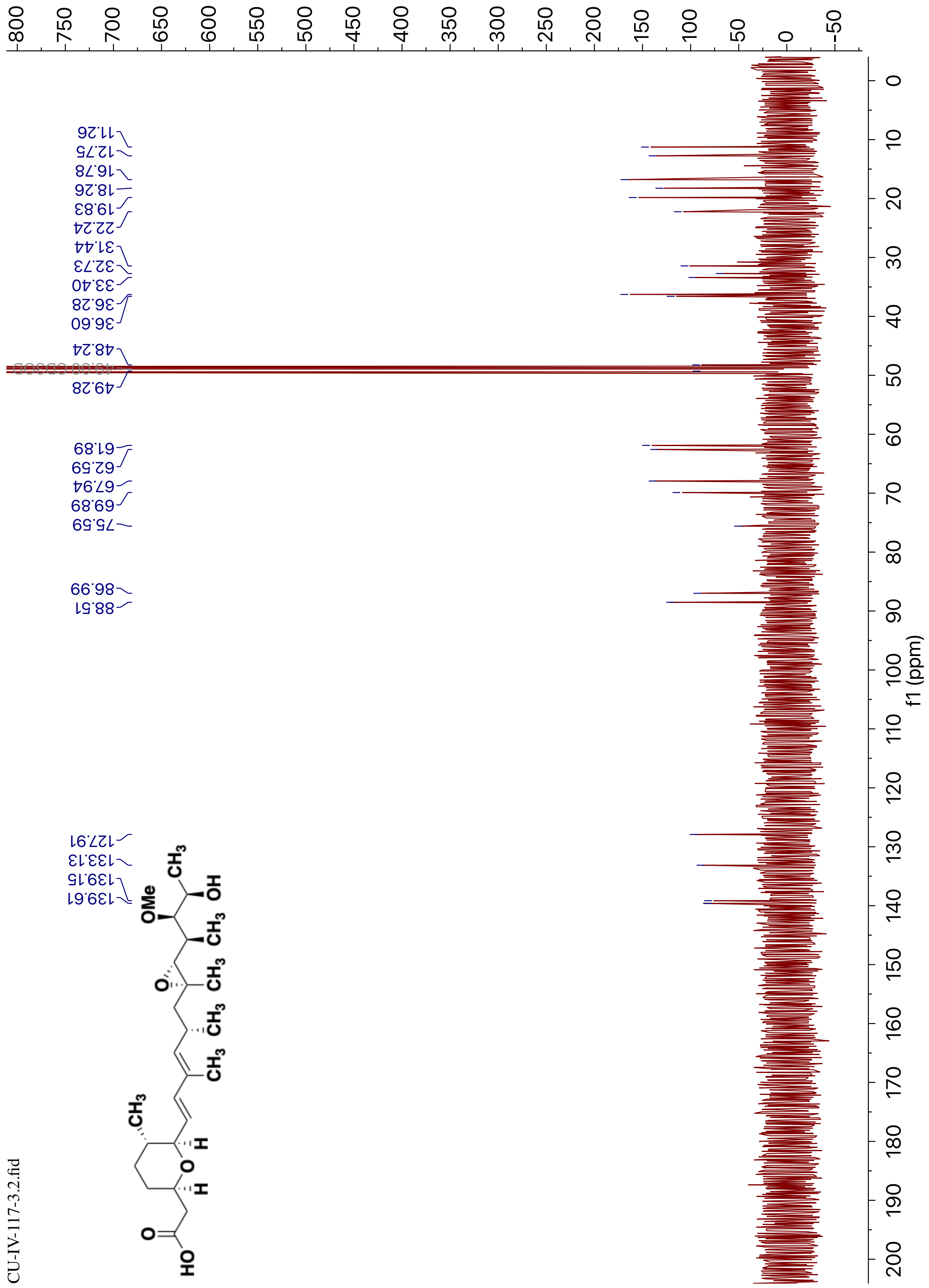
CU-IV-117-3.2.fid
11.26
12.75
16.78
18.26
19.83
22.24
31.44
32.73
33.40
36.28
36.60
48.24
49.28
61.89
62.59
67.94
69.89
75.59
86.99
88.51
127.91
133.13
139.15
139.61
f1 (ppm)

Mass Spectrometry & Proteomics Facility

Mass Spectrum SmartFormula Report

Analysis Info

Analysis Name	D:\Data\0426\CU-IV-117.d	Acquisition Date	4/26/2022 2:47:49 PM	
Method	0408 tune low.m	Operator	BDAL@DE	
Sample Name	CU-IV-117	Instrument / Ser#	micrOTOF II	8213750.10314
Comment	direct infusion			

Acquisition Parameter

Source Type	ESI	Ion Polarity	Positive	Set Nebulizer	0.4 Bar
Focus	Not active	Set Capillary	4500 V	Set Dry Heater	180 °C
Scan Begin	50 m/z	Set End Plate Offset	-500 V	Set Dry Gas	4.0 l/min
Scan End	1650 m/z	n/a	n/a	Set Divert Valve	Waste

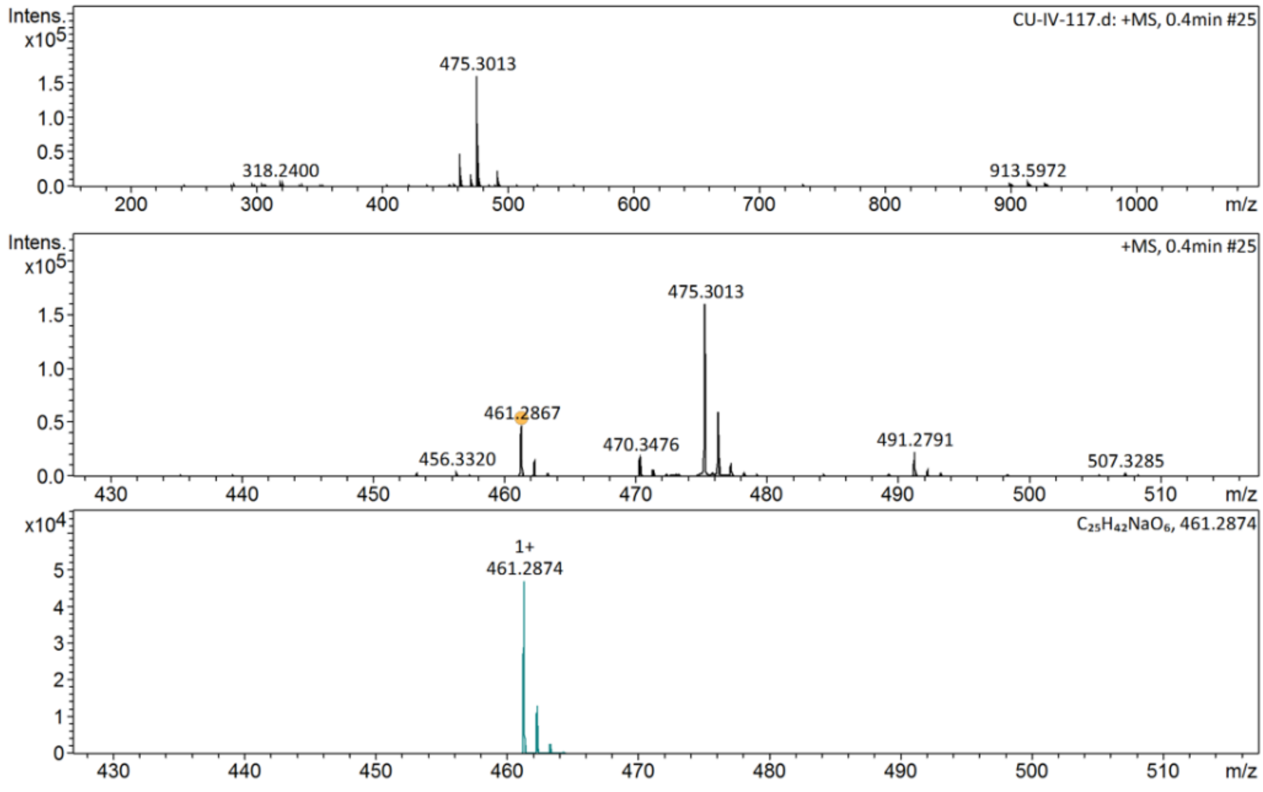

Meas. m/z	#	Ion Formula	m/z	err [ppm]	Mean err [ppm]	rdb	N-Rule	e⁻ Conf
461.286723	1	C25H42NaO6	461.287360	1.4	-0.3	4.5	ok	even

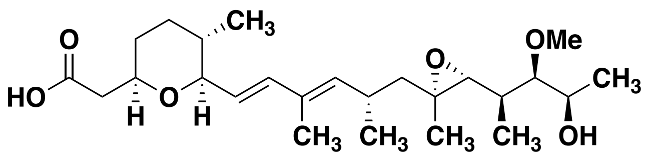

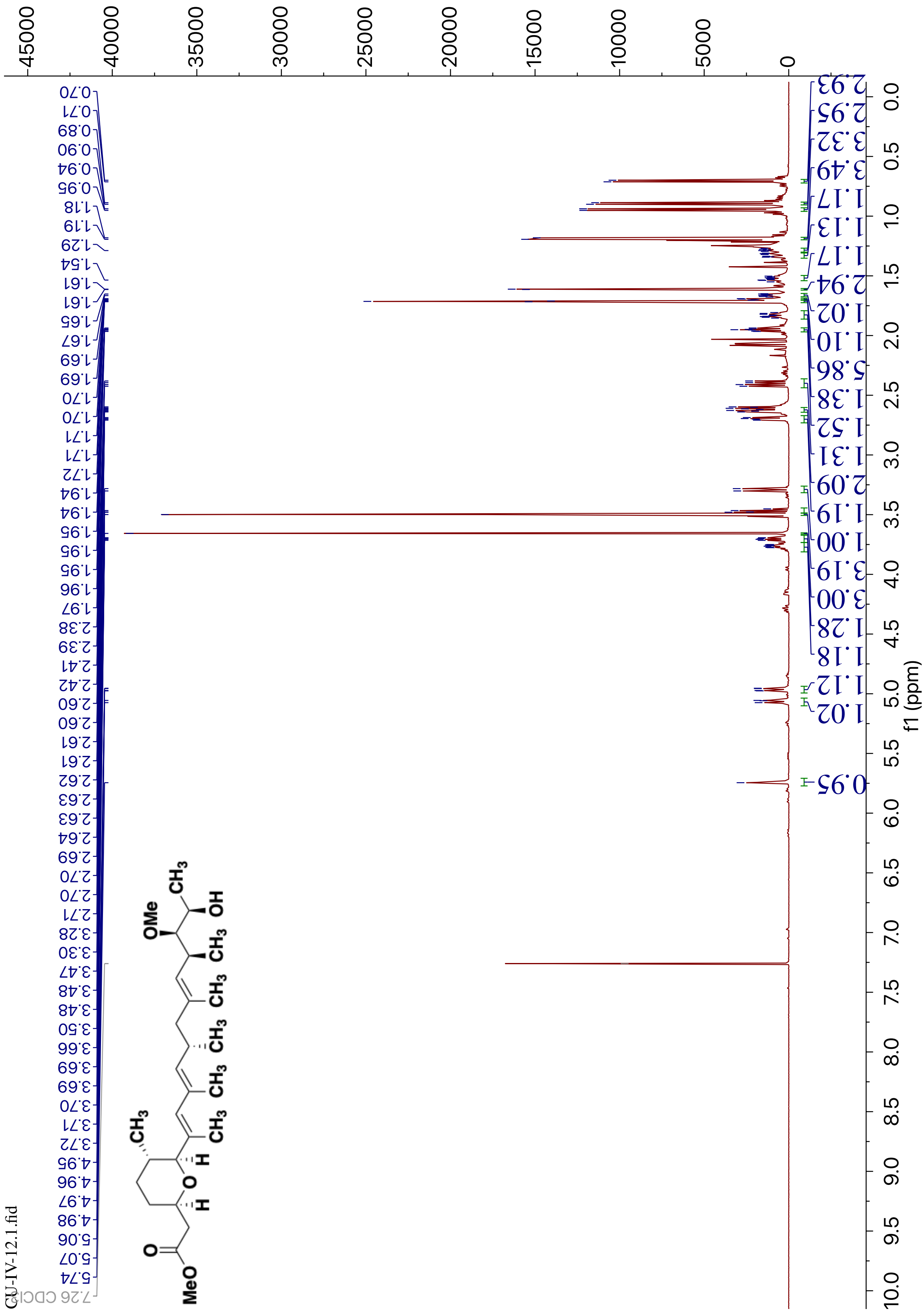
GU-IV-12.1.fid
f1 (ppm)
7.26 CDCl3

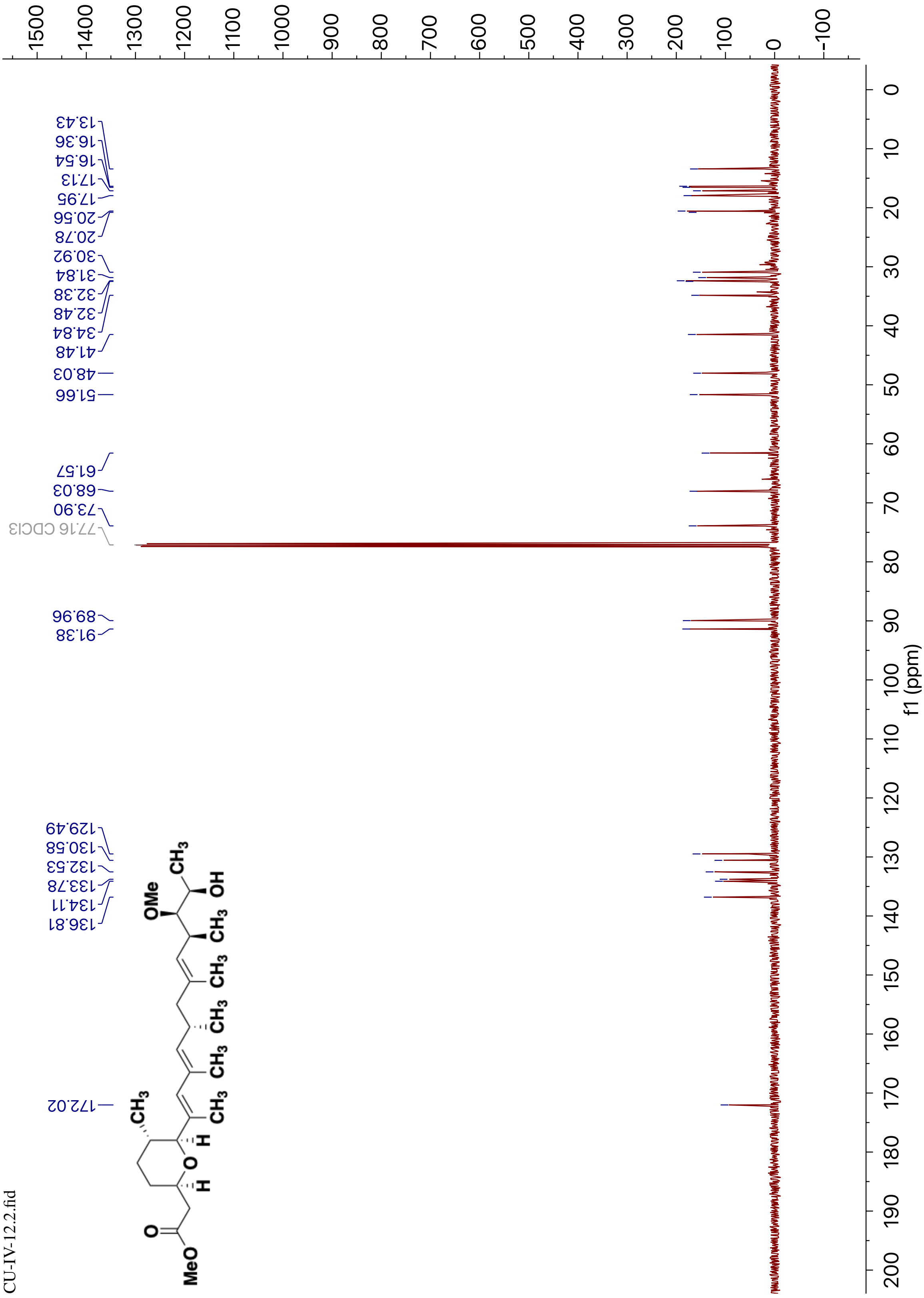
CU-IV-12.2.fid
13.43
16.36
16.54
17.13
17.95
20.56
20.78
30.92
31.84
32.38
32.48
34.84
41.48
48.03
51.66
61.57
68.03
73.90
77.16 CDCl3
89.96
91.38
129.49
130.58
132.53
133.78
134.11
136.81
172.02
f1 (ppm)

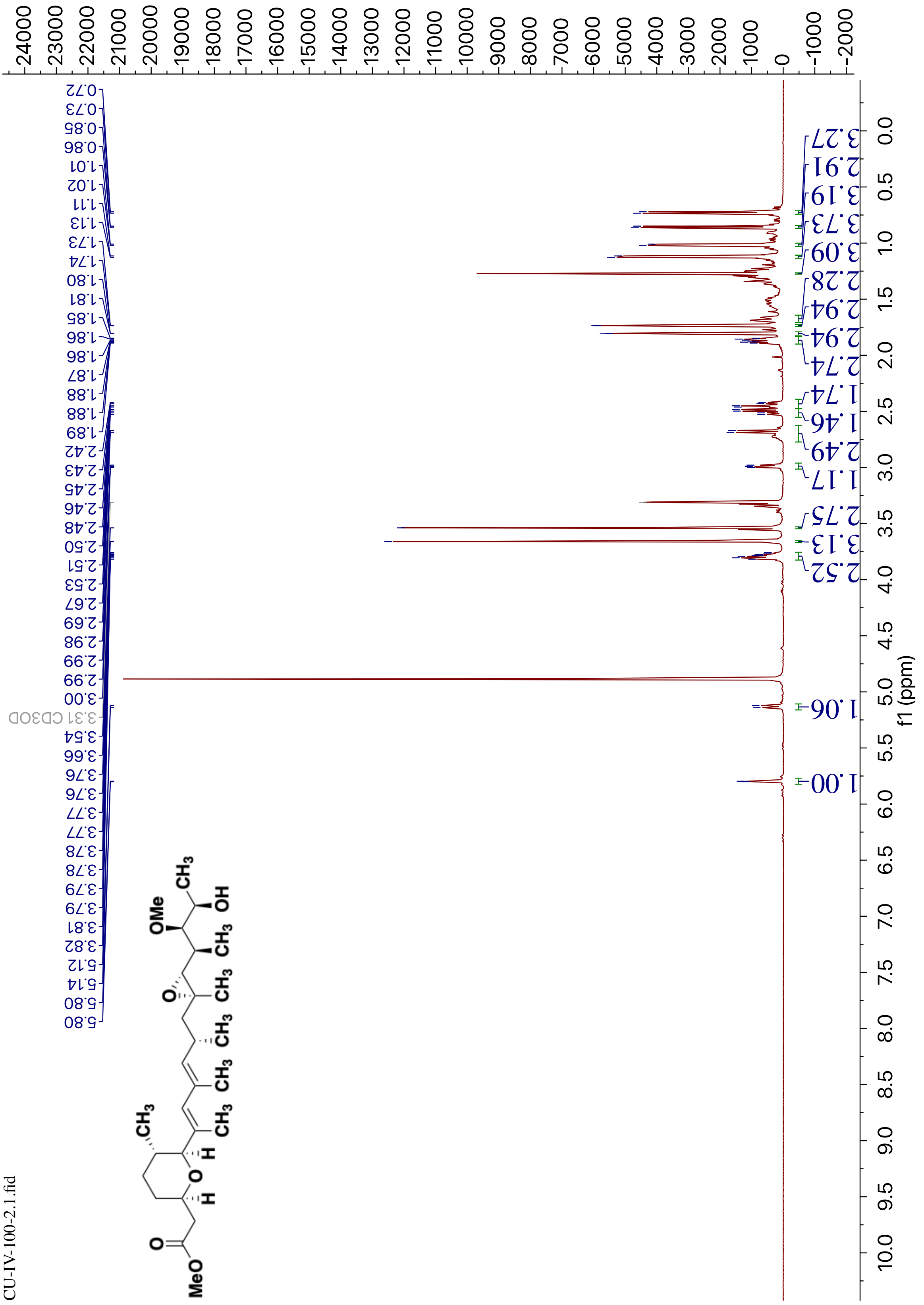
CU-IV-100-2.1.fid
3.31 CD3OD
f1 (ppm)

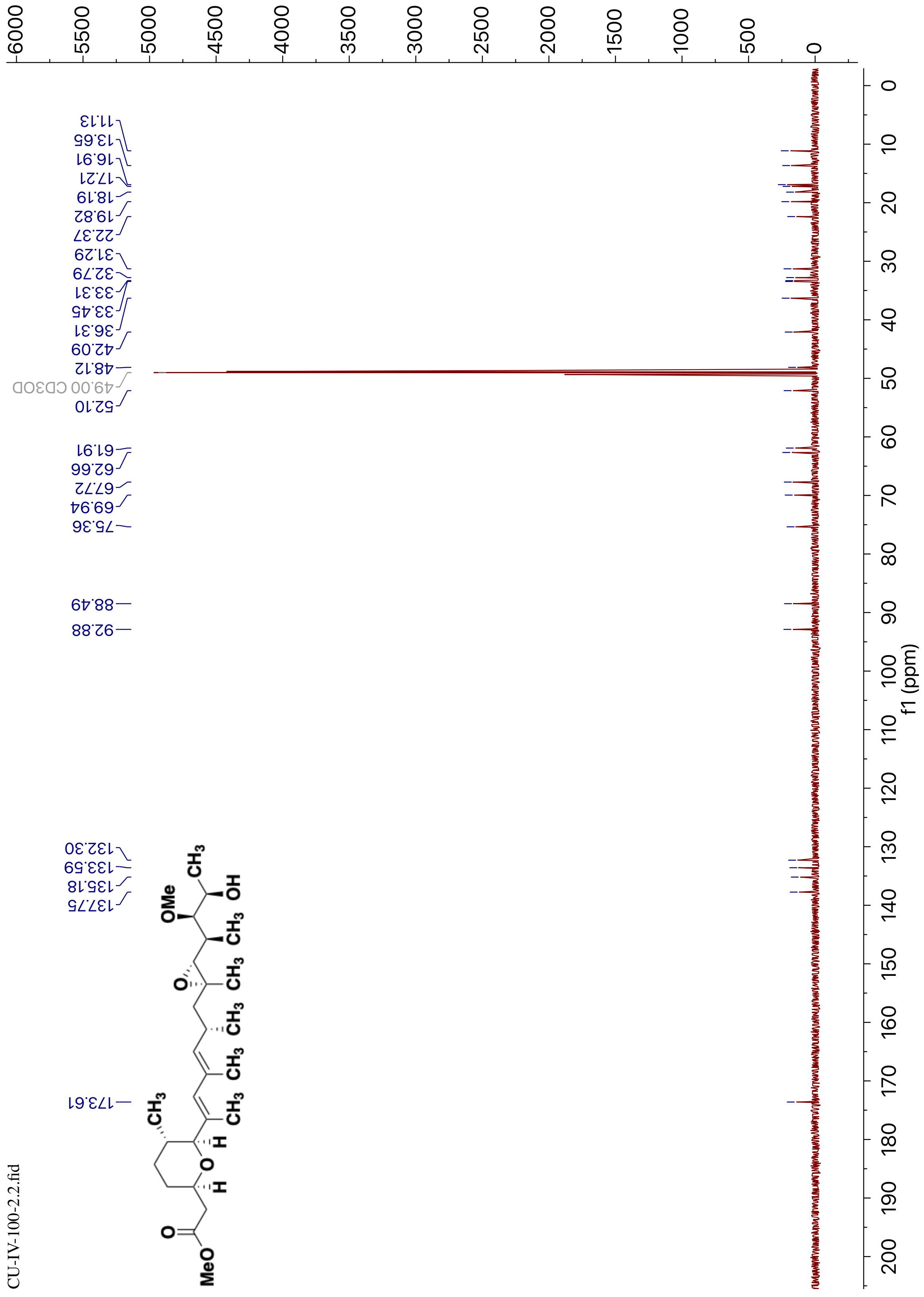
CU-IV-100-2.2.fid
11.13
13.65
16.91
17.21
18.19
19.82
22.37
31.29
32.79
33.31
33.45
36.31
42.09
48.12
49.00 CD3OD
52.10
61.91
62.66
67.72
69.94
75.36
88.49
92.88
132.30
133.59
135.18
137.75
173.61
f1 (ppm)

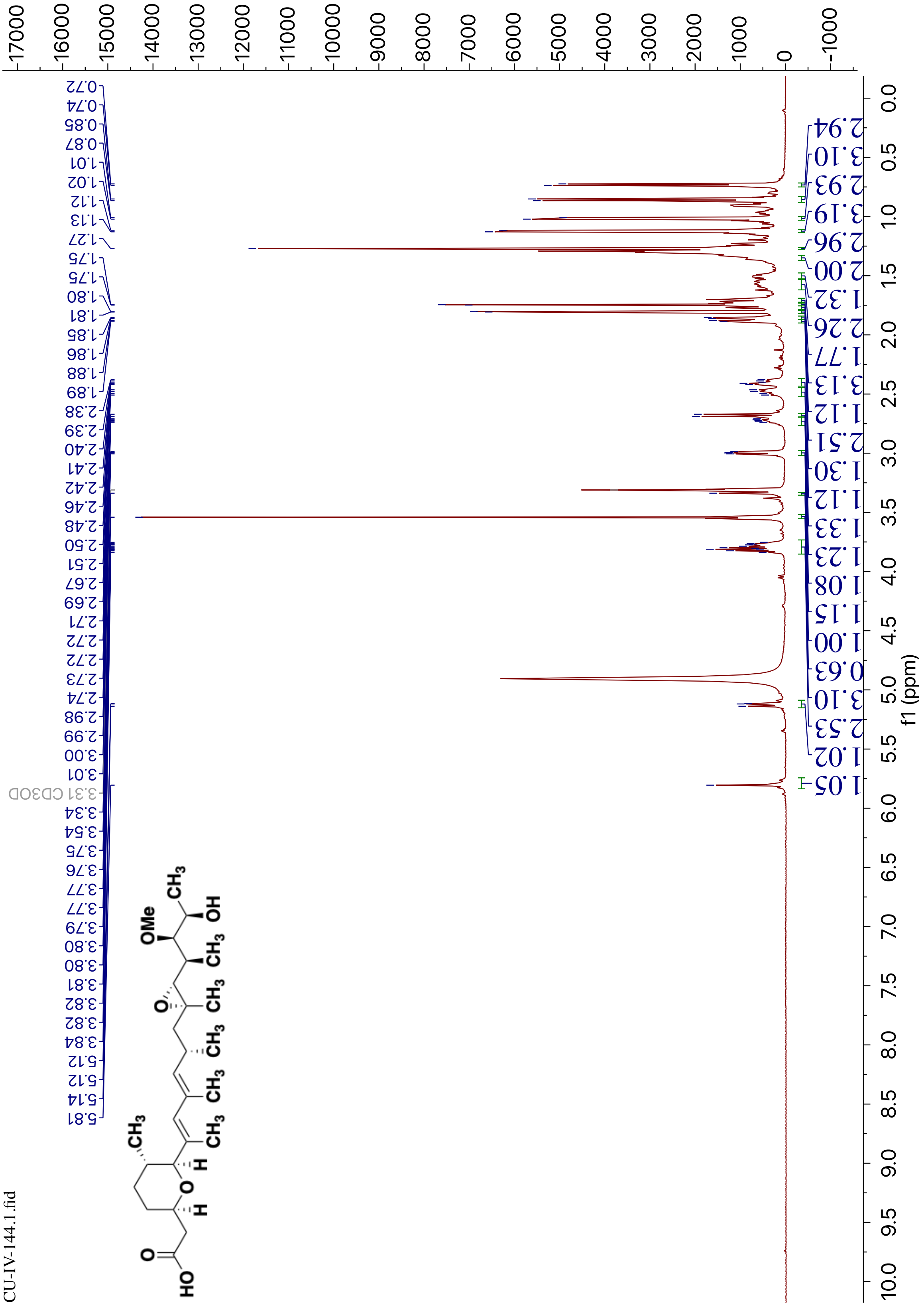
CU-IV-144.1.fid
0.72
0.74
0.85
0.87
1.01
1.02
1.12
1.13
1.27
1.75
1.75
1.80
1.81
1.85
1.86
1.88
1.89
2.38
2.39
2.40
2.41
2.42
2.46
2.48
2.50
2.51
2.67
2.69
2.71
2.72
2.72
2.73
2.74
2.98
2.99
3.00
3.01
3.31 CD3OD
3.34
3.54
3.75
3.76
3.77
3.77
3.79
3.80
3.80
3.81
3.82
3.82
3.84
5.12
5.12
5.14
5.81
2.94
3.10
2.93
3.19
2.96
2.00
1.32
2.26
1.77
3.13
1.12
2.51
1.30
1.12
1.33
1.23
1.08
1.15
1.00
0.63
3.10
2.53
1.02
1.05
f1 (ppm)
OMe
OH
HO
O
H

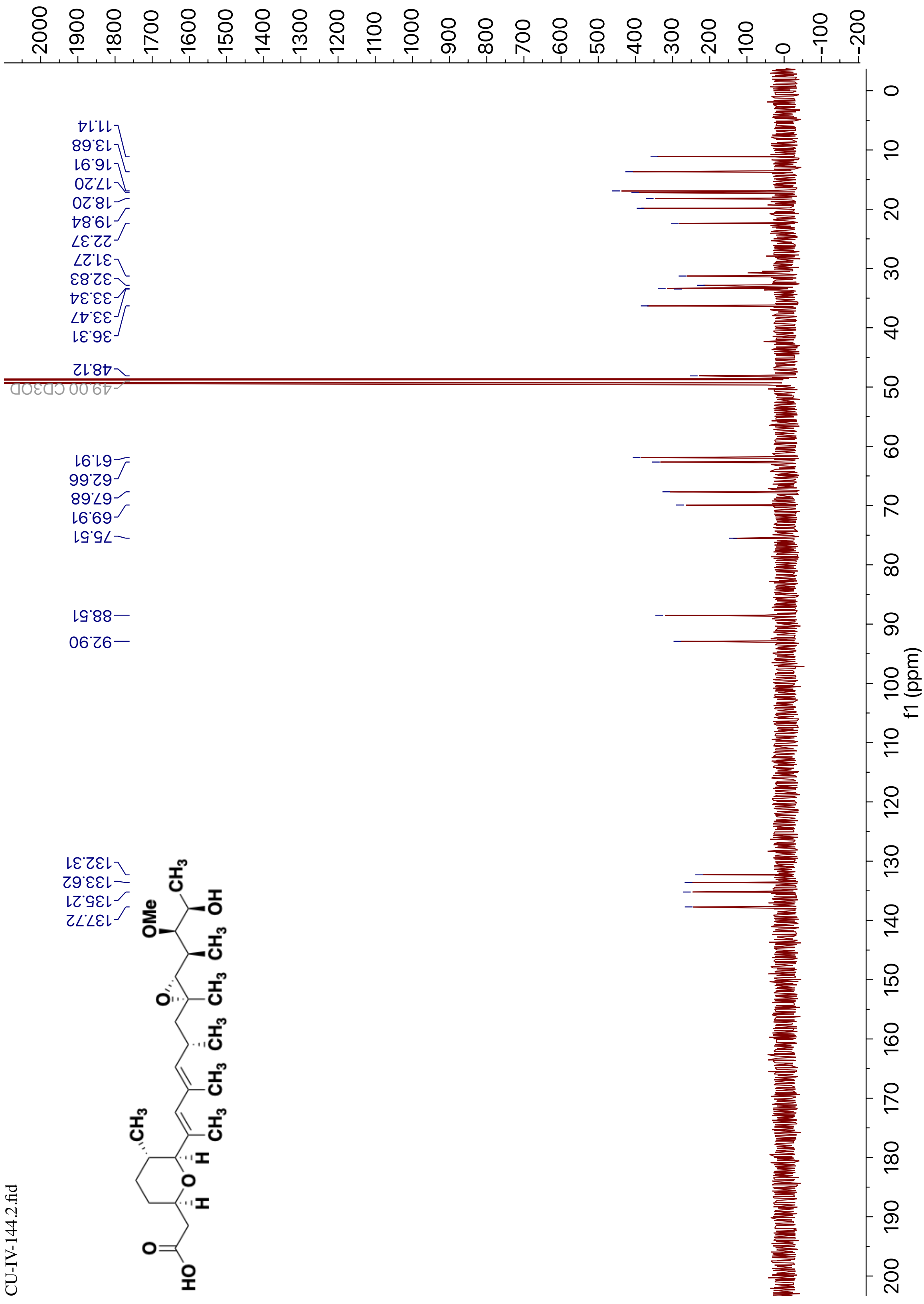
CU-IV-144.2.fid
11.14
13.68
16.91
17.20
18.20
19.84
22.37
31.27
32.83
33.34
33.47
36.31
48.12
49.00 CD3OD
61.91
62.66
67.68
69.91
75.51
88.51
92.90
132.31
133.62
135.21
137.72
f1 (ppm)

Mass Spectrometry & Proteomics Facility

Mass Spectrum SmartFormula Report

Analysis Info

Analysis Name	D:\Data\0524\CU-IV-144.d	Acquisition Date	5/24/2022 2:27:33 PM	
Method	0524 tune low.m	Operator	BDAL@DE	
Sample Name	CU-IV-144	Instrument / Ser#	micrOTOF II	8213750.1 0314
Comment	direct infusion			

Acquisition Parameter

Source Type	ESI	Ion Polarity	Positive	Set Nebulizer	0.4 Bar
Focus	Not active	Set Capillary	4500 V	Set Dry Heater	180 °C
Scan Begin	50 m/z	Set End Plate Offset	-500 V	Set Dry Gas	4.0 l/min
Scan End	1650 m/z	n/a	n/a	Set Divert Valve	Waste

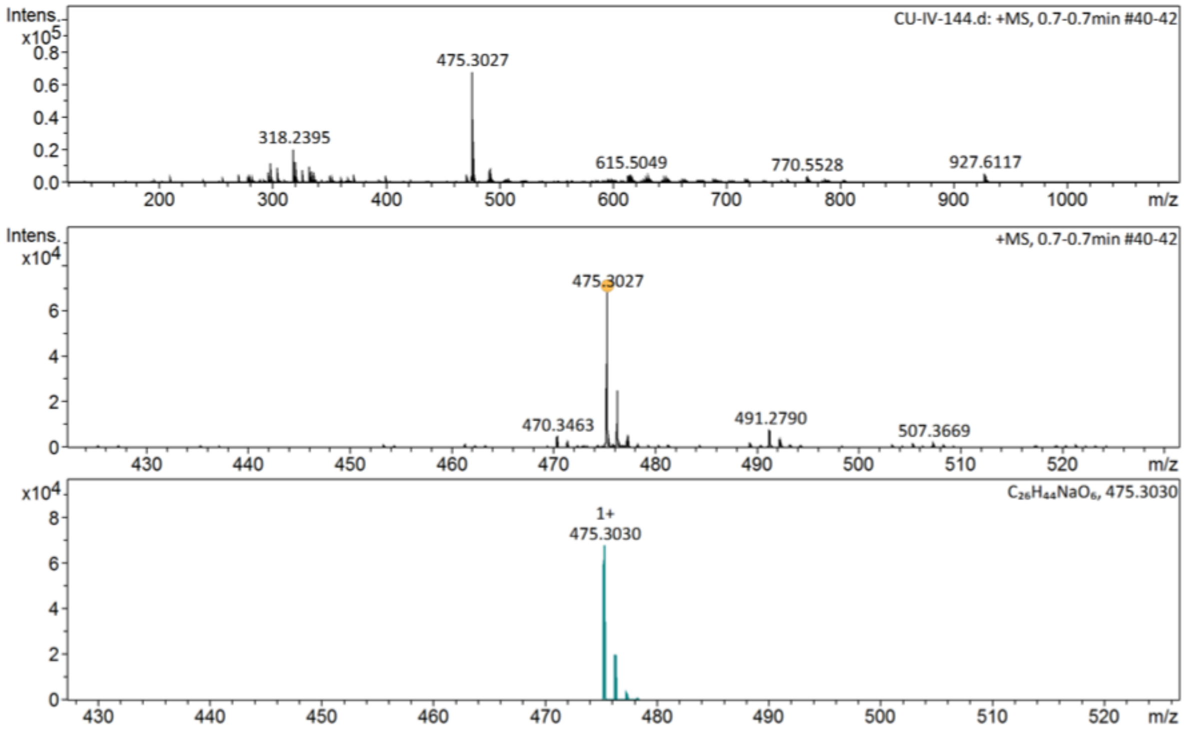

Meas. m/z	#	Ion Formula	m/z	err [ppm]	Mean err [ppm]	rdb	N-Rule	e⁻ Conf
475.302692	1	C26H44NaO6	475.303010	0.7	-1.2	4.5	ok	even

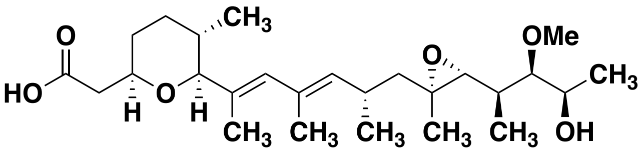

Supplement: Supplementary file 1 — Supplementary Material [file CMDC-21-e70309-s001.pdf]
